# From crystal structure to 3D printing to virtual reality in the classroom

**DOI:** 10.1107/S2056989025007868

**Published:** 2025-09-09

**Authors:** Dilek K. Dogutan, Meghan G. Sullivan, Christina Wolfskill

**Affiliations:** a Department of Chemistry and Chemical Biology Harvard University Cambridge MassachusettsUSA; Harvard University, USA

**Keywords:** crystal structure, 3D printing, virtual reality, active learning

## Abstract

We introduce a pedagogical approach to teaching structural chemistry in which students at all academic levels obtain .cif files from readily accessible crystal structure databases, print the corresponding structures using a 3D printer, and visualize the molecules within a virtual platform to acquire a full 360° view of the molecules. This engaging, active learning in-class activity enhances students’ understanding of key topics in a chemistry curriculum, such as coordination chemistry, symmetry elements, aromaticity, conjugation, crystallography, and many other topics that are often challenging to grasp using traditional one-dimensional tools (*e.g.*, chemical formulas in lecture notes) or two-dimensional representations (*e.g.*, textbook diagrams, PowerPoint presentations, or whiteboard drawings).

## Abstract 2

A pedagogical approach to teaching structural chemistry is introduced. This new methodology involves obtaining crystal structures from crystal structure databases, 3D printing models of molecules, and visualizing the molecules in a virtual classroom in a 360° view.

## Introduction

As science constantly evolves for the betterment of human experience, the way we teach science, technology, engineering, and mathematics (STEM) advances concurrently. From big lecture halls to small classrooms to laboratories, there are new ways of teaching and consequently learning STEM. For example, affordable and readily available 3D printing modules have been successfully incorporated to explain structures at the molecular level (Kitson *et al.*, 2014 ▸; Chen *et al.*, 2014 ▸; Brown *et al.* 2018 ▸, 2019 ▸). Such approaches have inspired chemistry educators to adopt 3D printing in subjects that can be somewhat challenging to the first-time undergraduate STEM students including symmetry, molecular structure, and coordination chemistry. Another contributing factor to the increasing popularity of 3D printing in chemistry and biochemistry teaching (Scalfani & Vaid, 2014 ▸; Kitson, *et al.*, 2014 ▸; Rodenbough *et al.*, 2015 ▸; Fourches & Feducia, 2018 ▸; Brannon, *et al.*, 2020 ▸; Savchenkov, 2020 ▸; Aristov *et al.*, 2022 ▸; Jurgenson, 2022 ▸, Eng & Valdez, 2025 ▸) is that it offers an alternative to model kits based on a variety of construction items including Styrofoam balls (Kenney, 1958 ▸; Birk & Foster, 1989 ▸), screw-on bottle caps (Siodłak, 2013 ▸), plastic bottles (Bindel, 2022 ▸), and paper-based materials (Goodsell *et al.*, 2024 ▸). These model kits are not only time-consuming, but also cumbersome when it comes to accurately representing the structures of chemical compounds. In contrast, 3D printing can enhance STEM teaching when combined with crystal structure depositories and extended databases such as the Cambridge Structural Database (CSD) [https://www.ccdc.cam.ac.uk (accessed 2020); Groom *et al.*, 2016 ▸] together with software like *Mercury* (Macrae *et al.*, 2020 ▸) to describe topics such as aromaticity, conjugation and chirality. Other software packages such as *CrystalMaker* for simulating minerals in geology (Palmer, 2015 ▸), and *VESTA* offer an alternative platform for 3D observation of volumetric and morphologic properties of crystals (Momma & Izumi 2011 ▸).

Besides 3D printing, utilizing Virtual Reality (VR) headsets has been another breakthrough in STEM pedagogy, across in-person classrooms, virtual organic, and biochemistry laboratories (Casas & Estop, 2015 ▸; Ferrell *et al.*, 2019 ▸; Dai *et al.*, 2020 ▸; Qin *et al.*, 2021 ▸; Williams *et al.*, 2022 ▸; Abbasi *et al.*, 2023 ▸; Arman *et al.*, 2023 ▸; Stella *et al.*, 2023 ▸; Bejjarapu *et al.*, 2024 ▸; Mojsoska *et al.*, 2024 ▸; Sohail *et al.*, 2025 ▸), and other online platforms (Quishpe-Armas *et al.*, 2015 ▸; Liou *et. al*, 2016 ▸; Caro *et al.*, 2018 ▸; Qin *et al.*, 2021 ▸), and engineering (Rodenbough, *et al.*, 2015 ▸). VR sets have been shown to facilitate real-time, interactive experiences, thereby enhancing student engagement and participation in classroom teaching (Ferrell *et al.*, 2019 ▸). Additionally, VR technology has offered pregnant and deployed soldiers the opportunity to undertake organic chemistry laboratory experiments in a virtual laboratory setting (Williams *et al.*, 2022 ▸). In addition to its use in teaching STEM classes, VR technology has already been incorporated in various disciplines including medical education (Ahlberg *et al.*, 2007 ▸; Khan *et al.*, 2019 ▸; Baniasadi *et al.*, 2020 ▸; Portelli *et al.*, 2020 ▸), drug design (Norrby *et al.*, 2015 ▸), and chemical safety (Srinivasan *et al.*, 2022 ▸).

**Figure 1:**
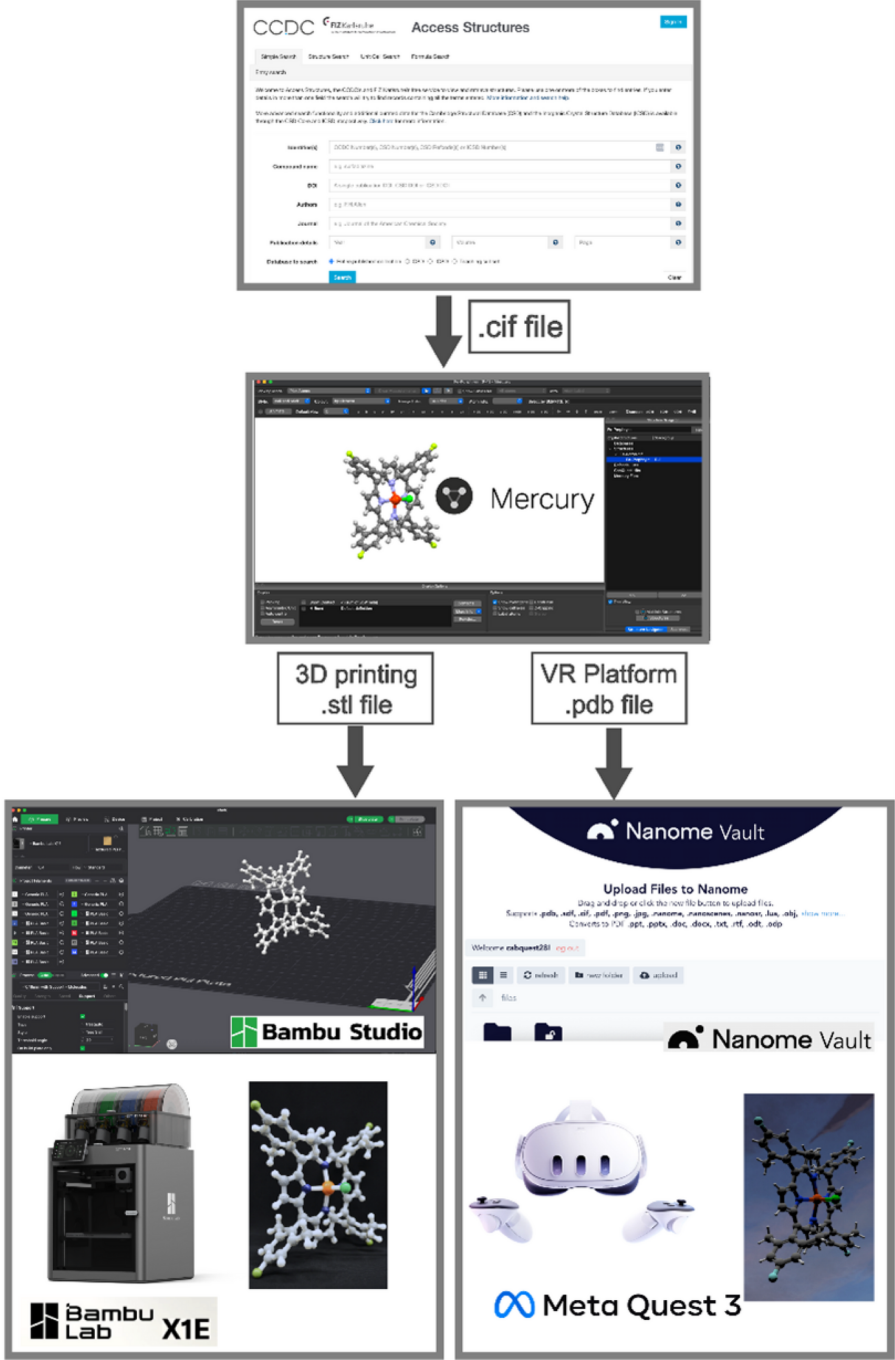
Flowchart showing the process used to obtain crystallographic data and convert it into a 3D printed structure that can be manipulated by students in the real world, as well as a VR structure that can be manipulated in virtual reality.

In this study, we introduce a new pedagogical approach to structural chemistry teaching at all undergraduate levels – freshmen, sophomore, juniors, and seniors (or equivalent designations outside north America) – with the following learning objectives: (1) accessing and utilizing crystallographic information files (.cif) deposited in the CSD database; (2) employing the CCDC’s free software package *Mercury* to visualize the properties of small chemical compounds; (3) converting .cif files to .stl files compatible with Bambu 3D printers to obtain the physical models; (4) colouring atoms to facilitate understanding of the molecular structures and their properties; (5) using the interface program *Nanome* (2020 ▸) to visualize the structures in a VR environment for a 360° view of the molecules; (6) measuring bond lengths, bond angles, and torsional (dihedral) angles between atoms; and (7) observing crystal packing, hydrogen bonding, and other intermolecular interactions among molecules (Fig. 1 ▸). The final component of this engaging active learning exercise involves acquiring feedback from the participants to highlight the benefits of combining 3D printing with VR classrooms, in comparison to their more traditional learning experiences – 1D (*e.g.*, chemical formulas), and 2D (*e.g.*, images in textbooks, and power point slides, or drawings on a blackboard). The survey results indicate that 75% of participants reported that using 3D printing in combination with VR sets somewhat or significantly enhanced their learning. Our study demonstrates that integrating 3D printing with VR sets improved students’ understanding of topics, such as aromaticity, conjugation, symmetry elements, point groups, chirality, and the measurement of bond lengths and bond angles. To the best of our knowledge, this is the first active learning exercise that assesses the value of combining 3D printing of small molecules with a VR platform allowing students to directly compare these approaches with traditional learning methods.

## Procedure

The backbone of this module relies on having the .cif file of the molecule of interest. Given the various crystallographic databases, such as the CSD, the Inorganic Crystal Structure Database (ICSD), the Protein Data Bank (PDB), and Crystallographic Open Database (COD), there are limitless opportunities for students to (i) obtain crystal structures of small organic or inorganic molecules or large biomolecules like proteins, (ii) print them using economical 3D printers, and (iii) visualize them using VR headsets with a full 360° view. One such workflow, which was used by undergraduate students to learn about porphyrin molecules, is shown in Fig. 1 ▸.

**Figure 2:**
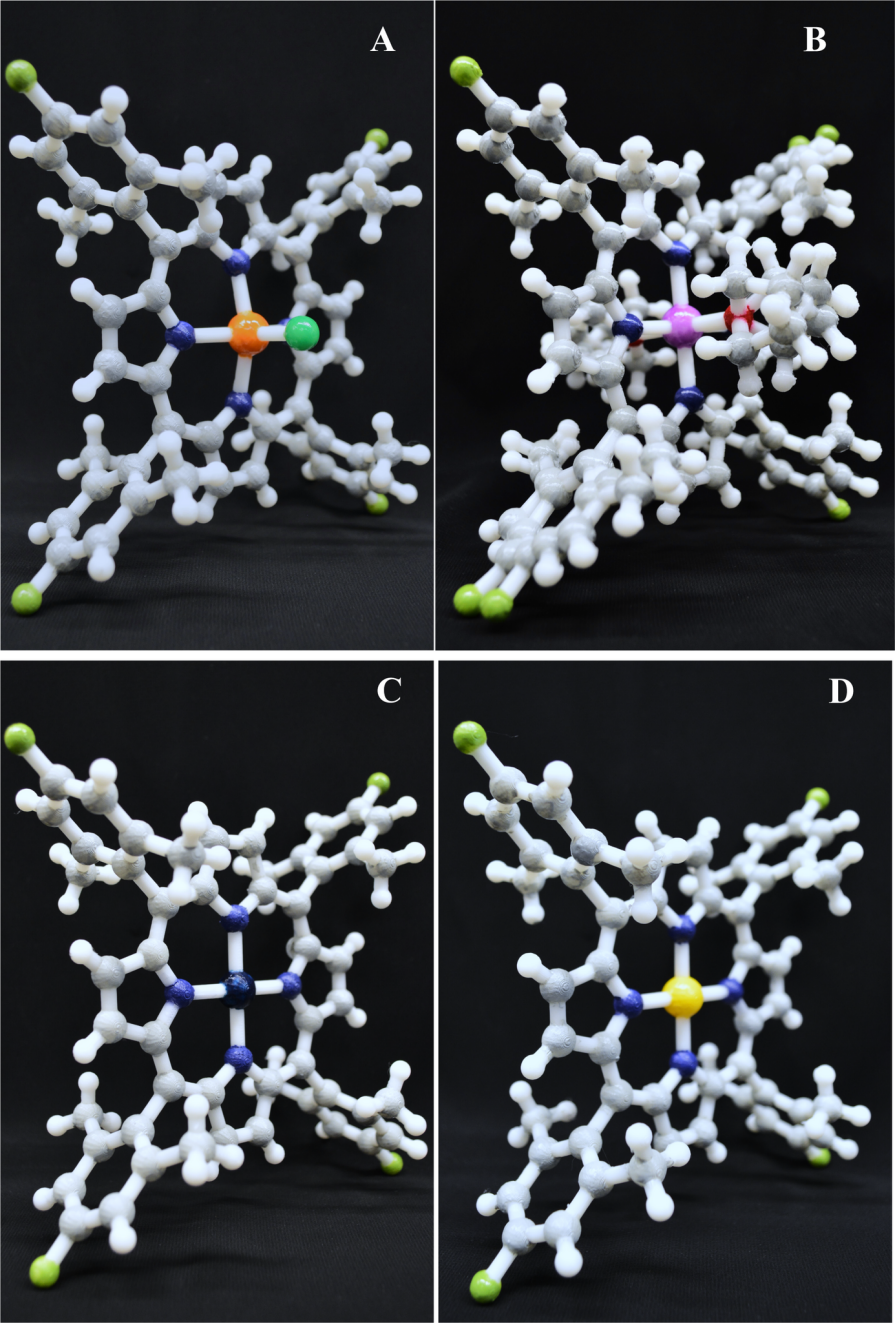
3D printed models of Fe porphyrin (A), Mg porphyrin (B), Pt porphyrin (C), and Pd porphyrin (D) using white filament, then coloured with nail polish.

The module requires students to have a laptop (Windows or Mac) with CCDC free *Mercury* version 2024.3.1 and Bambu Studio version 2.0.1.50 as well as a Meta Quest 3 VR headset with *Nanome* version 1.24 and a Nanome account. The goal of the exercise is to help students understand the chemical structure, stereochemistry, and crystal packing of various small molecules, such as porphyrins using three different modalities: in 2D using *Mercury* software (Step 1), in 3D using 3D printed models of the molecules (Step 2), and in virtual 3D using the VR headsets and *Nanome* software (Step 3). The effectiveness of each approach was then assessed utilizing a survey among the participants of this study. Each student was assigned one of the following four CSD identifiers (CSD Refcodes) for the selected porphyrin molecules used throughout the module: JUGYIX (Fe porphyrin; Brown *et al.*, 2023 ▸), JUGYET (Mg porphyrin; Brown *et al.*, 2023 ▸), GOPZEU (Pt porphyrin; Dash *et al.*, 2024 ▸), and CEPHEO (Pd porphyrin; Crisp, *et al.*, 2022 ▸). In preparation for the class, the teaching staff prepared 3D printed models of each of the four porphyrins using white filament (Fig. 2 ▸). In order to successfully 3D print the molecules, the default settings in Bambu Studio were modified to add slim tree supports to reduce the print speed and layer height. A complete list of the modified print settings along with the print time and approximate filament quantity and cost for each molecule is provided in the supporting information (SI) (Appendix B, Tables S2–S3). The photos of a metal-free porphyrin showing the supports obtained during 3D printing are provided in the supporting information (Fig. SF11). These supports were subsequently removed to present the porphyrin structures in a clear form. In Step 1, the students downloaded the .cif files for their given molecule from the CSD database and were introduced to the process that had already been followed to 3D print the compounds using *Mercury* and *Bambu Studio software* (SI, Figs. SF1–SF11).

In Step 2, to save time the participants of the study were provided with the pre-printed models of the porphyrins shown in Fig. 2 ▸, as 3D printing of these molecules can take anywhere from 4 to 8 h depending on the geometry of the porphyrin and its substituents. The participants were then asked to identify the locations of the various atoms on the 3D printed models. The atoms were then painted using nail polish according to Corey, Pauling, and Koltun (CPK) colour conventions (SI, Table S1). Upon completing the 3D printing portion of the module, the students moved on to Step 3, the VR component of the activity where they first used *Mercury* to export a .pdb file of their porphyrin, which could then be loaded into *Nanome* platform, an application preinstalled on the VR headsets. The students were then (i) provided with the VR headsets and the hand controllers, (ii) logged into the Nanome account that they setup to upload the .pdf file of their porphyrin, and (iii) joined in a shared room where the teaching staff was also (virtually) present. With all the students present, the instructor demonstrated how to load the porphyrins, and how to measure bond lengths, bond angles, and torsional angles using the built-in tools in *Nanome*. Each student was then given a turn to take over the presenter role and tried out the applications using the *Nanome* tools. The students were also shown how to get similar information about bonds from the *Mercury* software on their laptops. After the joint VR session, each student was given an opportunity to start their own room in *Nanome*, load their molecule, and take measurements of it. They then took a survey in which they answered several questions about the geometry of their specific molecule as well as their opinions on the utility and engagement of the various modalities in the module. A sample student handout with detailed instructions for Steps 1–3 is provided in the Supporting information (SI Sections S1–S3, Figs. SF12, SF31). Although this exercise was initially carried out as an engaging activity for Harvard undergraduate students who volunteered to participate in the activity and had completed a year of general chemistry, it can be easily incorporated into the classroom teaching. The module (excluding the 3D printing step) can be completed in approximately 20 minutes in groups of three.

## Feedback from the participants

After completion of the described module, students were asked to complete a brief survey to assess their experience (SI, Appendix A). The survey comprised four sections: (i) demographics (SI, Section S6.1), (ii) usability (SI, Section S6.2), (iii) learning (SI, Section S6.3), and (iv) engagement (SI, Section S6.4). The survey took students a median time of 5 minutes to complete. The exact questions used, and comprehensive survey results can be found within the supporting information (SI, Demographics: Figs. SF32–SF40, Usability: Figs. SF41–SF48, Learning: Figs. SF49–SF56, Engagement: Figs. SF57–SF62).

**Figure 3:**
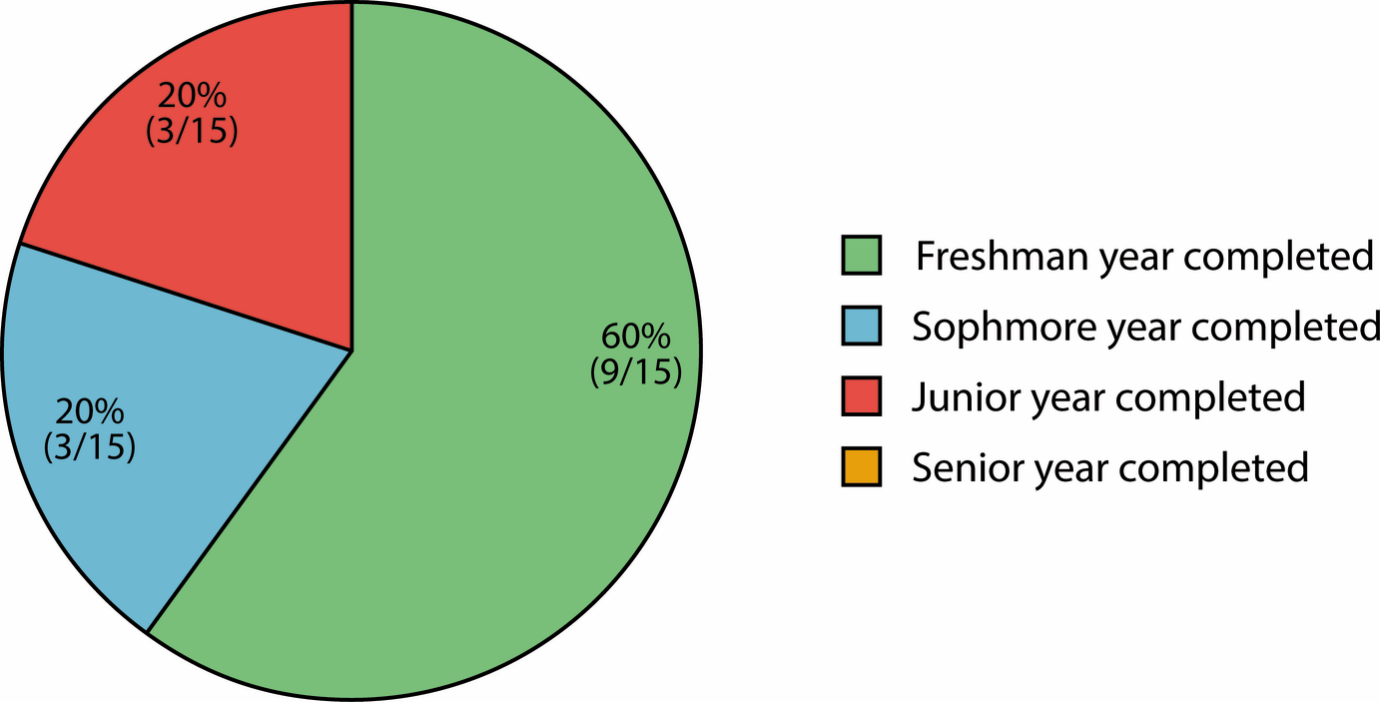
Survey results displaying the demographic breakdown of undergraduate respondents as a function of year-of-study completed at Harvard College.

In total, 15 undergraduate students completed the module and the survey, the majority (60%) of whom had just concluded their freshman year of coursework (Fig. 3 ▸). This was reflected in their responses regarding pre-module familiarity with topics including the CSD, manipulating crystal structures, 3D printing, and VR platforms. Most students reported having little-to-no experience with any of these topics, so our module provided a first-ever look at molecular structures in three-dimensions for the majority of participants. All students were studying STEM topics, with the majority (67%) being chemistry concentrators (*i.e.*, majors; SI Fig. SF34).

Most students found CSD and 3D printing related tasks within the module to be ‘not difficult’, indicating the ease of usability regarding these aspects of the module (SI, Figs. SF41–SF44). The respondents found that visualizing the 3D structure of the molecule within the VR platform was the most challenging, with 27% finding it ‘somewhat difficult’ and another 27% finding it ‘difficult’ (SI, Fig. FS45). This difficulty was further supported by comments received when asked about the most challenging aspect of the module. Notably, when asked about the difficulty of subsequently identifying bond lengths and angles within VR, 87% of students claimed that identifying bond lengths was either not difficult or only somewhat difficult, (SI, Fig. FS46). These results suggest that students had the most difficulty with the initial use of the VR headsets, and hand controllers, including orienting themselves in the virtual environment, using the trigger, grab, and joystick buttons, and navigating the different tools within *Nanome*. However, as one student claimed, and as the usability results regarding measuring bond lengths and angles in VR corrobo­rate (SI, Figs. SF46–SF48), with some practice the exercise became much easier. For our study, students were allotted approximately 15 minutes to complete the virtual reality section. Therefore, we encourage future users of this or similar modules to ensure students have ample time (30 minuntes to 1 hour) to acquaint themselves with the VR headsets and controllers, especially if they are first-time VR users. Overall, survey results highlight the accessibility usability of this module as a whole, especially given the demographic of majority first-time users.

**Figure 4:**
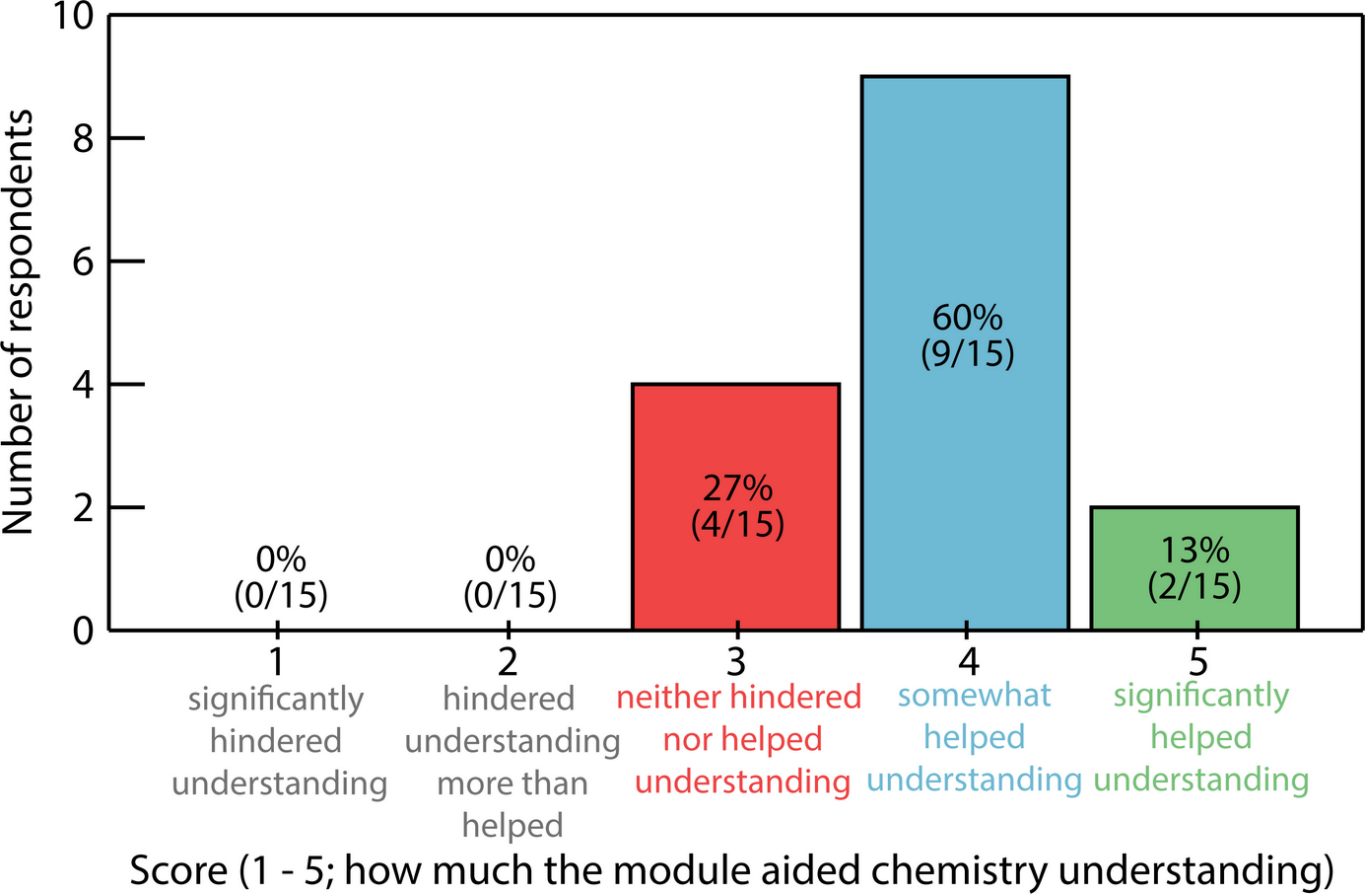
Survey results showing how the respondents ranked the helpfulness of this module in their understanding of chemistry concepts.

Of the participants, 93% found this module useful in their overall learning of chemistry (SI Figs. SF49–SF52). More specifically, nearly all students reported that 3D printing (100%; SI Fig. SF49), and virtual reality (93%; SI Fig. SF50) helped them understand molecular properties such as geometry, bonding, bond lengths and angles, and coordination environments. When asked to rank how the exercise impacted their understanding of chemistry on a scale of 1 to 5 (1 = this significantly hindered my understanding of chemistry, 2 = this hindered my understanding more than it helped my understanding of chemistry, 3 = this neither hindered nor helped my understanding of chemistry, 4 = this helped me understand chemistry concepts somewhat, 5 = this really helped me understand certain chemistry concepts), the median respondent answered a 4 (Fig. 4 ▸). Especially impressive were the results when students were asked to identify; (i) coordination geometry around the metal centre of their assigned structure, and (ii) all chemical elements present in their molecule. Despite a majority of the students having no prior familiarity with most topics covered in this module, 87% were able to accurately identify coordination geometry (square planar/square pyramidal/octahedral; SI Fig. SF53), and 60% were able to correctly assign all chemical elements present (SI, Fig. SF55).

**Figure 5:**
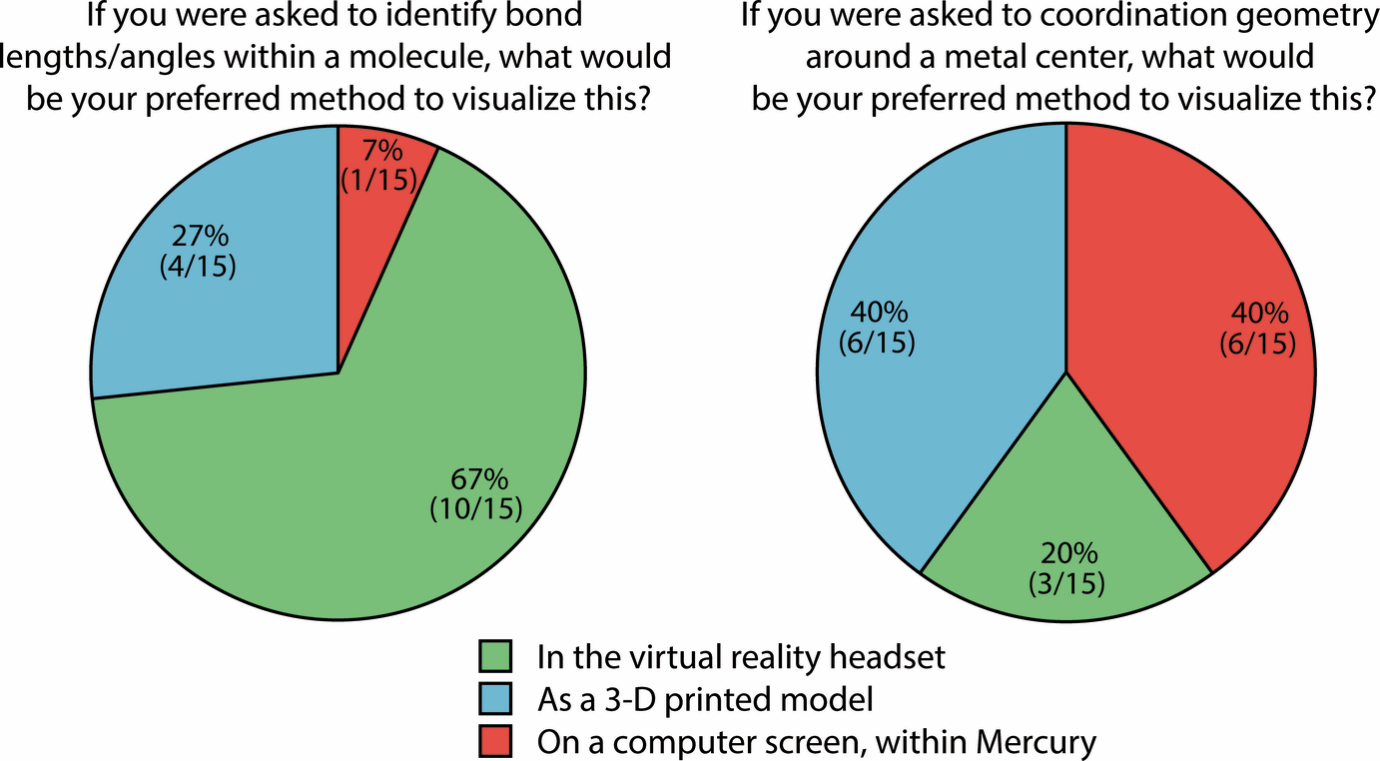
Survey results for queries (top) on visualization preferences for identifying bond lengths/angles (left) *versus* coordination geometry (right) across choices including virtual reality (green), 3D printed models (blue), or on a computer screen within *Mercury* (red).

Given the early exposure this module provided to undergraduate students who were in preliminary stages of their STEM careers, we were especially eager to understand how engaged our participants felt with the module; namely, their opinions on the three different ways of viewing 3D molecular structure (on a computer within *Mercury*, as a 3D printed model, or in virtual reality). The majority of students (80%) found that visualizing the crystal structure in virtual reality was the most engaging (SI, Fig. SF57) while the remaining students (20%) preferred engagement via the 3D printed model. None of the students (0%) found visualization on a computer screen within *Mercury* to be the most engaging. Interestingly, when students were asked what method was the most *intuitive*, or natural to understand, the majority (67%) opted for the 3D printed model, with the remainder selecting virtual reality and *Mercury* (27% and 7%, respectively, SI Fig. SF58). Furthermore, the respondents preferred virtual reality (67%) for assessment of bond lengths and angles but preferred a 3D printed model or *Mercury* (40% and 40%, respectively) for assessment of coordination geometry around a metal centre (Fig. 5 ▸). These results highlight the synergism that students can benefit from when a combination of visualization methods are used and substantiate the use of 3D printing and virtual reality as useful forms of engagement in the classroom.

## Conclusions

We developed and implemented a pedagogical initiative to introduce structural chemical knowledge to undergraduate students at all academic levels through the incorporation of the Cambridge Crystallographic Data Centre (CCDC) single-crystal X-ray database (Cambridge Structural Database) with 3D printing and virtual reality classroom. This immersive 3D full view learning exercise helped students with their understanding of various chemistry concepts, which may be challenging to first-time learners. The survey conducted on the undergraduate population shows that this active learning exercise offers many advantages over traditional 1D and 2D learning tools, especially with complicated structures.

**Conflicts of interest** The Authors declare that they have no conflict of interest.

The data supporting the results reported in this article can be accessed within the article, and through the supporting information.

## Supplementary Material

The supporting information including all the supporting data. DOI: 10.1107/S2056989025007868/oi2021sup2.docx
