# Supplementary material for: From crystal structure to 3D printing to virtual reality in the classroom
Source: Acta Crystallogr E Crystallogr Commun. 2025 Sep 9;81(Pt 10):889–94. doi: 10.1107/S2056989025007868 (PMC12498038; doi:10.1107/S2056989025007868)

Supporting information

| **Figure SF1** CCDC website. | S4 |
| --- | --- |
| **Figure SF2** How to download deposited .cif file | S4 |
| **Figure SF3** How to download and manipulate the .cif file of a compound in Mercury. | S5 |
| **Figure SF4** How to save the file as a .pdb file in Mercury. | S5 |
| **Figure SF5** How to upload a .pdb file into Nanome. | S5 |
| **Figure SF6** How to upload a .pdb file into Nanome Vault online platform. | S6 |
| **Figure SF7** How to covert .cif file to .stl file in Mercury. | S7 |
| **Figure SF8** How to upload .stl file to Bambu Studio software. | S7 |
| **Figure SF9** How to prepare .stl file to printing in Bambu Studio software. | S8 |
| **Figure SF10** How to prepare, slice, and print .stl file to printing in Bambu Studio software. | S9 |
| **Figure SF11** The built model of the molecule with the support. | S9 |
| **Table S1** Corey, Pauling, and Koltun (CPK) colour convention. | S10 |
| **Figure SF12** The buttons on the controllers. | S11 |
| **Figure SF13** How to choose Nanome application from the Library settings using VR headsets | S11 |
| **Figure SF14** How to enter the Nanome username and password. | S12 |
| **Figure SF15** How to download .pdf file using Entry List →Vault→Open→Shared→.pdb | S12 |
| **Figure SF16** How to manipulate the molecule in 3D VR platform. | S13 |
| **Figure SF17** How to manipulate shrink, enlarge, move backwards, and forwards the molecule in 3D VR platform. | S13 |
| **Figure SF18** How to turn on or off the external video view in 3D Virtual Reality room by using the menu available on the left form arm of the viewer. | S14 |
| **Figure SF19** How to turn on or off the external video view in 3D Virtual Reality room by using the menu available on the left form arm of the viewer | S14 |
| **Figure SF20** How to obtain **Structure Information** about the molecule in Mercury using the .cif file. | S15 |
| **Figure SF21** How to obtain the bonds lengths of the molecule in Mercury using the .cif file. | S15 |
| **Figure SF22** How to measure bond lengths between the atoms on the molecule using VR sets in the 3D platform. | S16 |
| **Figure SF23** How to obtain **All Angles** of the molecule in Mercury using the .cif file. | S17 |
| **Figure SF24** How to measure bond angles on the molecule using VR sets in the 3D platform. | S17 |
| **Figure SF25** How to obtain **All Torsions** of the molecule in Mercury using the .cif file. | S18 |
| **Figure SF26** How to measure the torsional/dihedral angle on the molecule using VR sets in the 3D platform. | S18 |
| **Figure SF27** How to pack molecules in Mercury. | S19 |
| **Figure SF28** Crystal packing view and molecular measurements displayed in the VR platform. | S19 |
| **Figure SF29** How to delete measurements in the 3D platform using the joystick button. | S20 |
| **Figure SF30** How to take screenshots in the 3D platform using the trigger button. | S20 |
| **Figure SF31** How to email screenshots of the molecule in the 3D platform using the trigger button. | S21 |
| **Table S2** The list of CCDC CSD Reference codes | S22 |
| **S6.1** Demographics | S23 |
| **S6.2** Usability | S24 |
| **S6.3** Learning | S26 |
| **S6.4** Engagement | S27 |
| **S7.1** Demographics Figures | S28 |
| **Figure SF32** Pie chart showing results of Demographics Question 1 – “*Are you over 18 years of age?.”* | S28 |
| **Figure SF33** Pie chart showing results of Demographics Question 2 – *“What year of study have you completed at Harvard College?”* | S28 |
| **Figure SF34** Pie chart showing results of Demographics Question 3 – *“How would you categorize your concentration?”* | S28 |
| **Figure SF35** Pie chart showing results of Demographics Question 4 – “*What is your concentration*?” | S28 |
| **Figure SF36** Pie chart showing results of Demographics Question 5 – *“What type of computer system are you using?”* | S29 |
| **Figure SF37** Bar graph showing results of Demographics Question 6a. | S29 |
| **Figure SF38** Bar graph showing results of Demographics Question 6b. | S29 |
| **Figure SF39** Bar graph showing results of Demographics Question 6c. | S30 |
| **Figure SF40** Bar graph showing results of Demographics Question 6d. | S30 |
| **S7.2** Usability Figures | S30 |
| **Figure SF41** Pie chart showing results of Usability Prompt 1 – “*Finding the crystal structure from CCDC was…”* | S30 |
| **Figure SF42** Pie chart showing results of Usability Prompt 2 – *“Converting the file to a 3D-printable format was…”* | S31 |
| **Figure SF43** Pie chart showing results of Usability Prompt 3 – *“Printing a 3D structure of the molecule was…”* | S31 |
| **Figure SF44** Pie chart showing results of Usability Prompt 4 – *“Identifying the identity of atoms on the printed structures was…”.* | S31 |
| **Figure SF45** Pie chart showing results of Usability Prompt 5 – “*Visualizing the 3D structure of the molecule in the VR platform was…”* | S31 |
| **Figure SF46** Pie chart showing results of Usability Prompt 6 – *“Identifying the bond lengths on the molecule using VR set was…”* | S32 |
| **Figure SF47** Pie chart showing results of Usability Prompt 7 – *“Identifying the angles of the bonds on the molecule using VR set was…”* | S32 |
| **Figure SF48** Pie chart showing results of Usability Question 10 – *“Did you experience any dizziness or disorientation when using the VR headset?”* | S32 |
| **S7.3** Learning Figures | S32 |
| **Figure SF49** Pie chart showing results of Learning Prompt 1 – “*Printing the 3D structure of the molecule helped me understand the properties.”* | S33 |
| **Figure SF50** Pie chart showing results of Learning Prompt 2 – *“Using the VR set helped me understand the properties (geometry, bonding, angles, coordination environment, packing) of the molecule.”* | S33 |
| **Figure SF51** Pie chart showing results of Learning Question 3 – *“Did you find this exercise helpful in learning chemistry?”* | S33 |
| **Figure SF52** Bar graph showing results of Learning Question 4 – *“How would you rank this exercise in your chemistry learning/understanding, on a scale of 1 to 5?* | S33 |
| **Figure SF53** Pie chart showing breakdown of CSD reference code assignments, indicated by Learning Question 5 – *“What was the CSD Reference Code of the structure you were assigned?*” | S34 |
| **Figure SF54** Pie chart showing breakdown of correct/incorrect coordination geometry assignments, indicated by the results of Learning Question 6. | S34 |
| **Figure SF55** Pie chart showing breakdown of correct/incorrect non-metal element assignments, indicated by the results of Learning Question 7. | S34 |
| **Figure SF56** Pie chart showing breakdown of correct/incorrect metal element assignments, indicated by the results of Learning Question 8. | S35 |
| **S7.4.** Engagement Figures | S35 |
| **Figure SF57** Pie chart showing results of Engagement Question 1 – *“Which method of looking at the crystal structure did you find most engaging?* | S35 |
| **Figure SF58** Pie chart showing results of Engagement Question 2 – *“Which method of looking at the crystal structure did you find most intuitive (natural to understand)?”* | S35 |
| **Figure SF59** Pie chart showing results of Engagement Question 3 – *“If you were asked to identify how molecules pack within a crystal structure, which would be your preferred method to visualize this?* | S36 |
| **Figure SF60** Pie chart showing results of Engagement Question 4 – *“If you were asked to identify bond angles within a molecule (e.g. which angles are close to 90°), which would be your preferred method to visualize this?”* | S36 |
| **Figure SF61** Pie chart showing results of Engagement Question 5 – *“If you were asked to identify bond lengths within a molecule, which would be your preferred method to visualize this.?*” | S36 |
| **Figure SF62** Pie chart showing results of Engagement Question 6 – “7. *If you were asked to identify coordination geometry around a metal within a molecule (e.g. square planar, square pyramidal, octahedral), which would be your preferred method to visualize this?”* | S37 |
| **Appendix A** The survey questions | S37 |
| **Appendix B** Bambu Studio Settings and Results | S44 |
| **Table S3** Bambu Studio Settings and Results for 3D Print | S45 |
| **Table S4** Summary of 3D Print Times and Cost | S45 |
| **References** | S46 |

**S1. 3D Printing and Visualizing Molecules in VR**

**S1.1. Requirements**

- Computer with access to the CCDC website and CCDC Free Mercury software.
- Meta Quest 3 VR Headset with Nanome software and a Nanome username and password

**S1.1.1. Part I – Getting the Structural Data and Converting It for 3D Printing and VR Viewing**

**S1. Step 1: Download Files**

Sign in to the CCDC website (<https://www.ccdc.cam.ac.uk>) and download the structural data file (.cif) for the molecule you will be visualizing from the CSD Database Identifier. You can provide your contact information in the fields at the bottom of the download form or opt out by clicking the link.


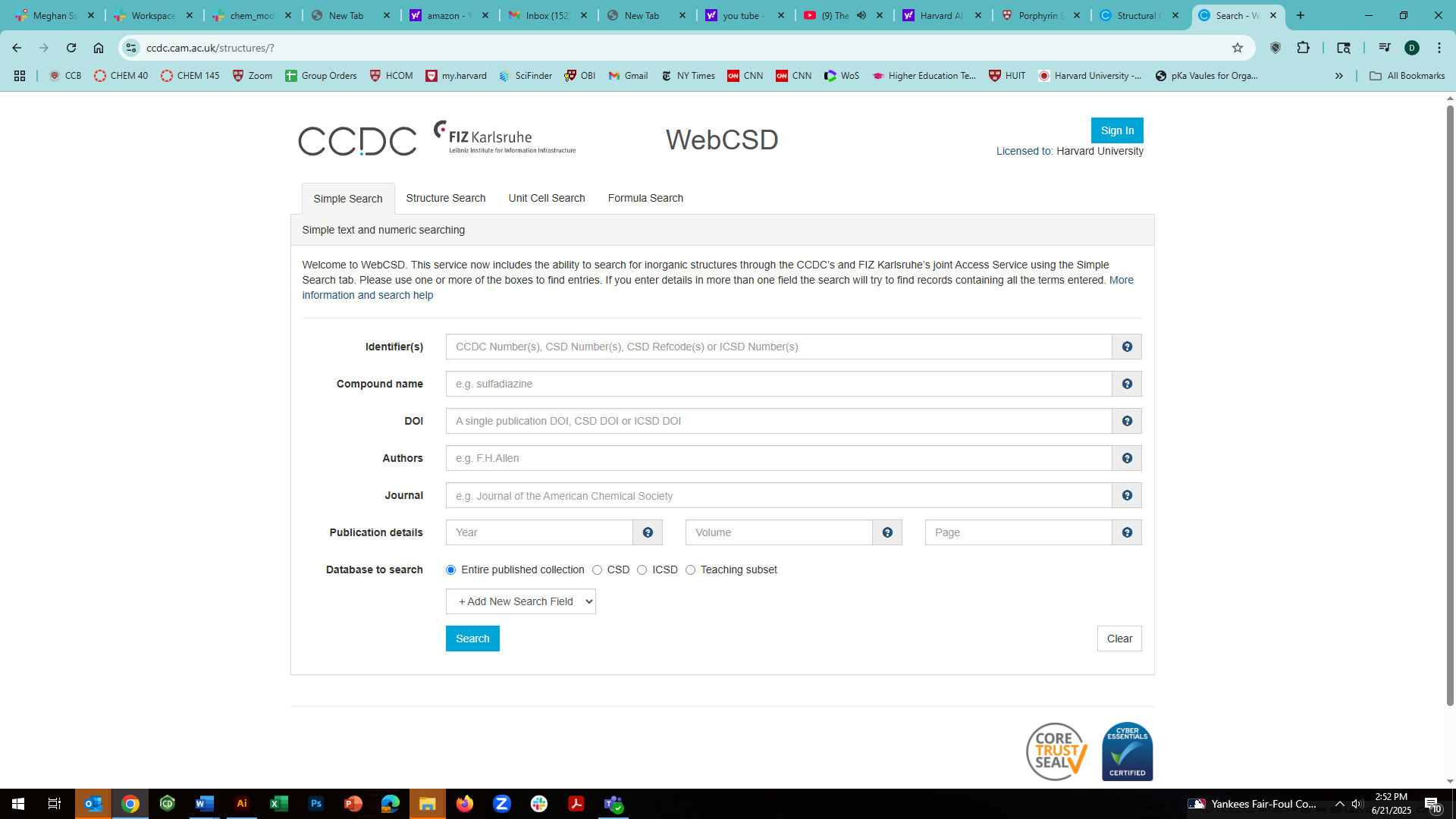


1. **Figure SF1** CCDC website.


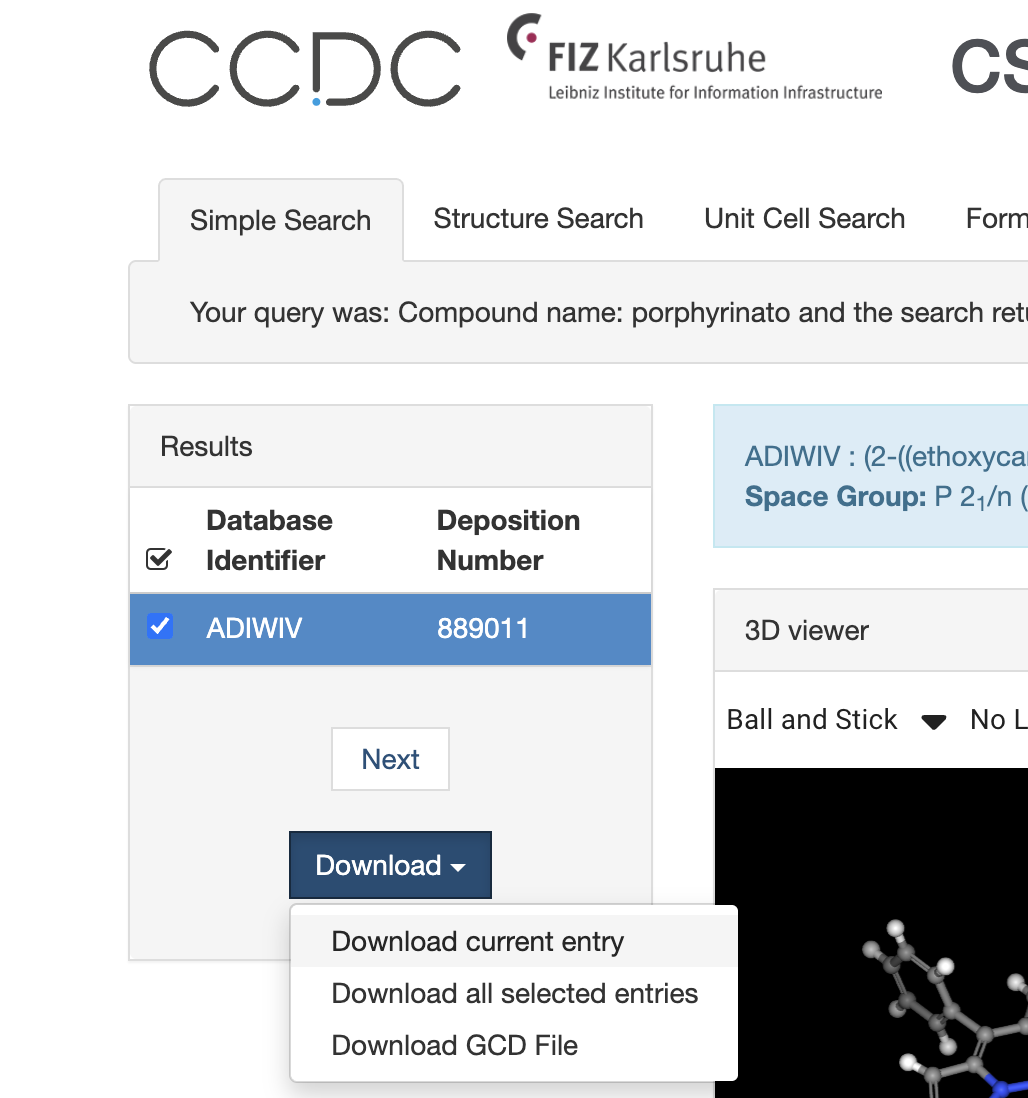

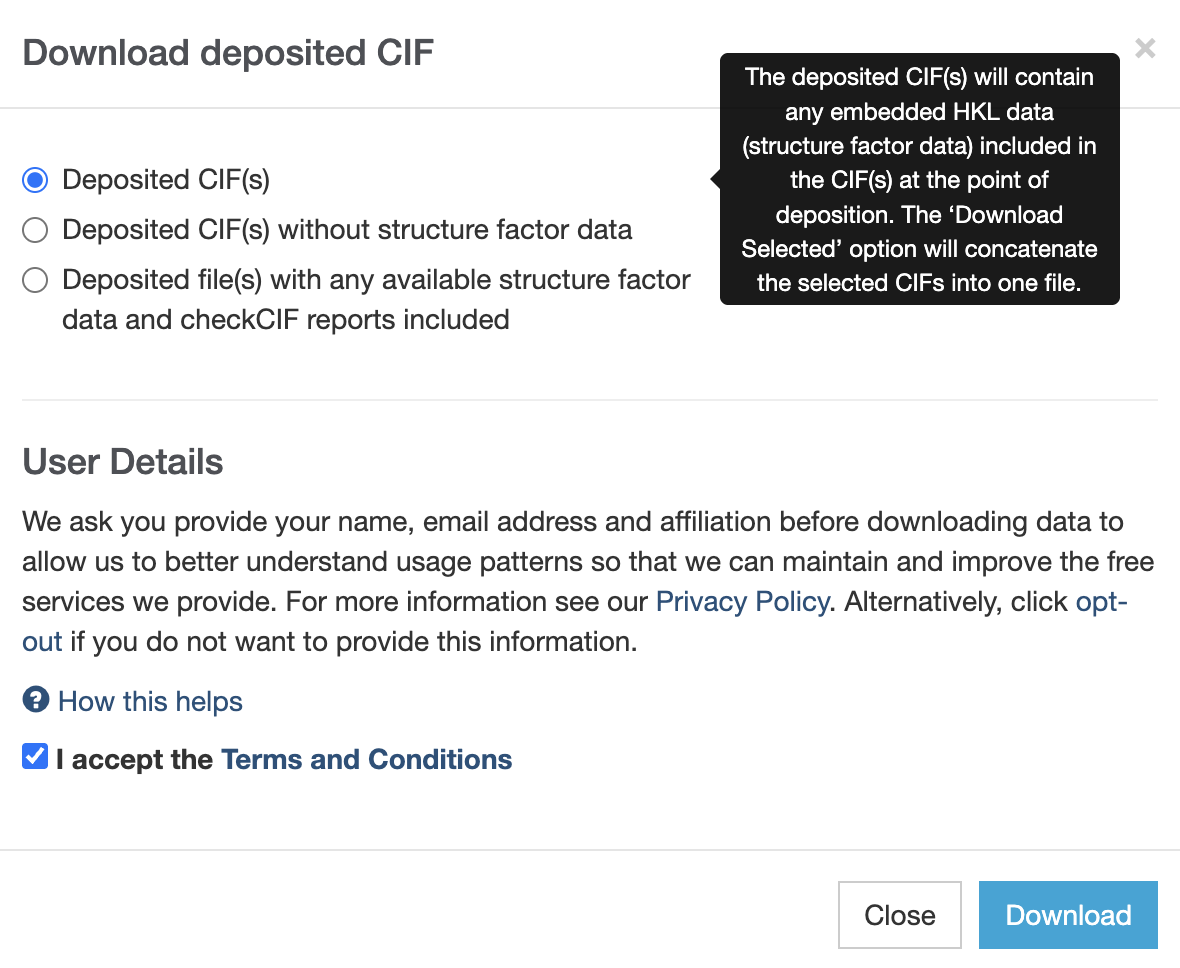


**Figure SF2** How to download deposited .cif file.

**S1. Step 2: Open in Mercury**Start the CCDC Mercury software, and select **File > Open**, then navigate to where you saved the downloaded .cif file and click **Open**. This will load the molecule and allow you to manupulate it.


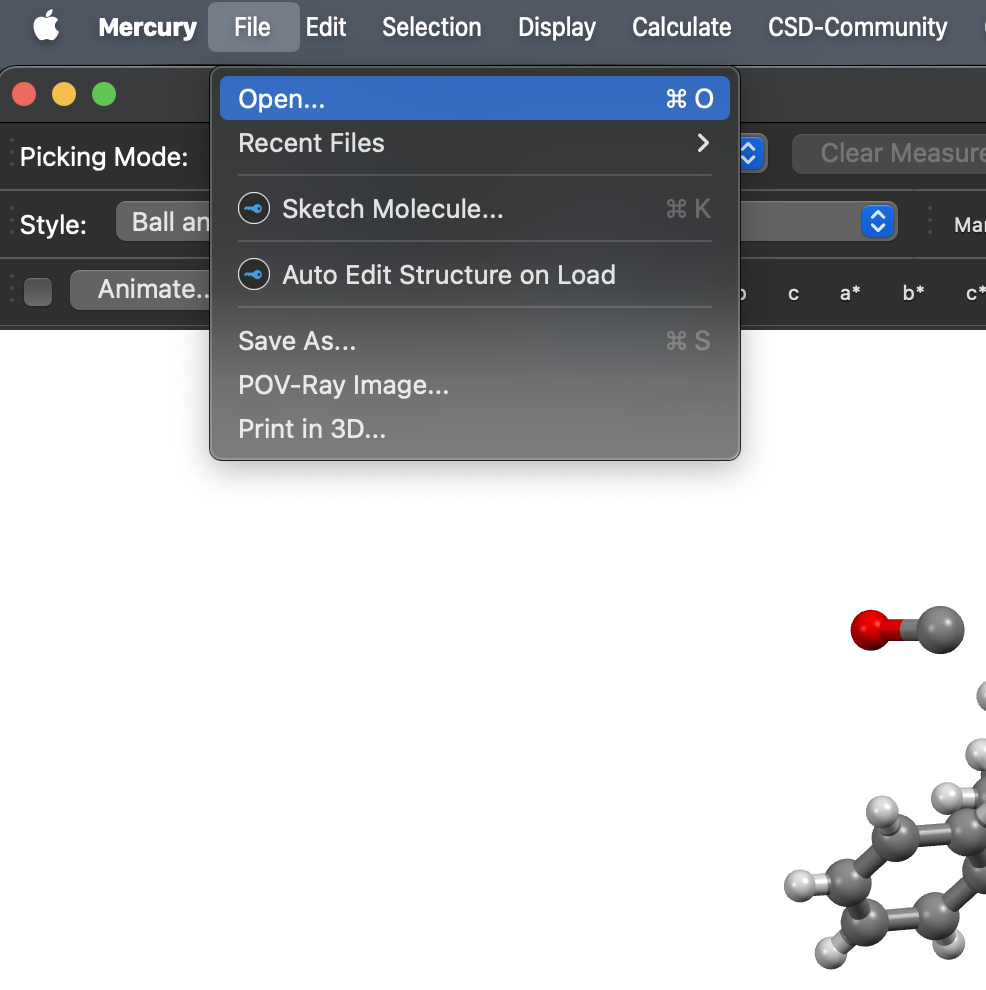


**Figure SF3** How to download and manipulate the .cif file of a compound in Mercury.

**S1. Step 3**: **Save as PDB and Upload to Nanome for VR Viewing**
In Mercury, select **File > Save As…** and save your molecule as a .pdb file.


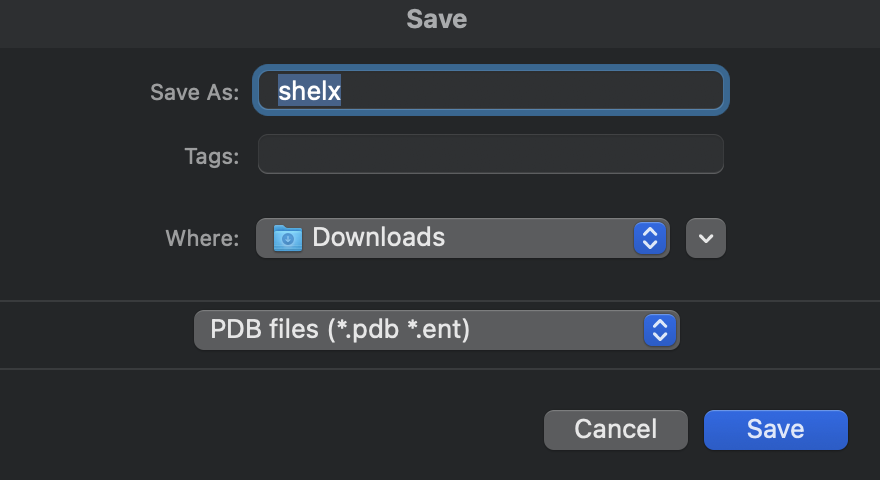


**Figure SF4** How to save the file as a .pdb file in Mercury.

In a web browser, navigate to the Nanome webpage (https://home.nanome.ai/) and log in with your Nanome username and password. At the top of the page, you will see a **Quick Drop** section, where you can upload files so you can view them in the Nanome application on your VR headset. Click **Upload Files > From Computer** and select the .pdb file of your molecule that you saved in Mercury. The filename will appear inside the Quick Drop box, and is now ready to be viewed in Nanome using the VR headset. You can also upload the pdf file with the VR instructions, which will allow you to see the instructions with your headset on.


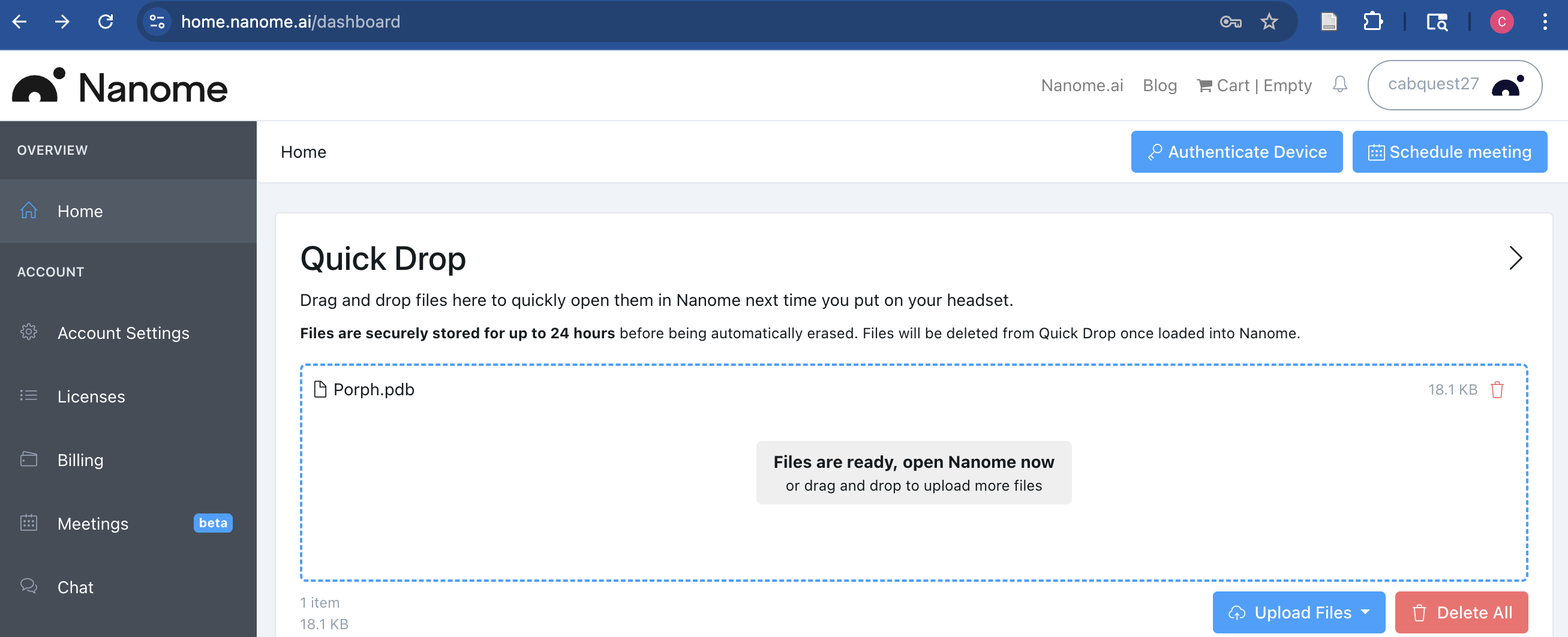


**Figure SF5** How to upload a .pdb file into Nanome.

Alternatively, Nanome provides a Vault online where files can be uploaded and then accessed within the application on the VR headset. To store files in the Vault, navigate to the Nanome Vault webpage (https://vault.nanome.ai/) and logging in with your Nanome username and password. Open the shared/PDB folder, and upload the .pdb file that you saved from Mercury by dragging and dropping the file from your computer or clicking the upload button. Files loaded via the Quick Drop option are temporary and will only load onto the headset a single time, and will then be deleted, but files saved to the Vault will be stored for access indefinitely.


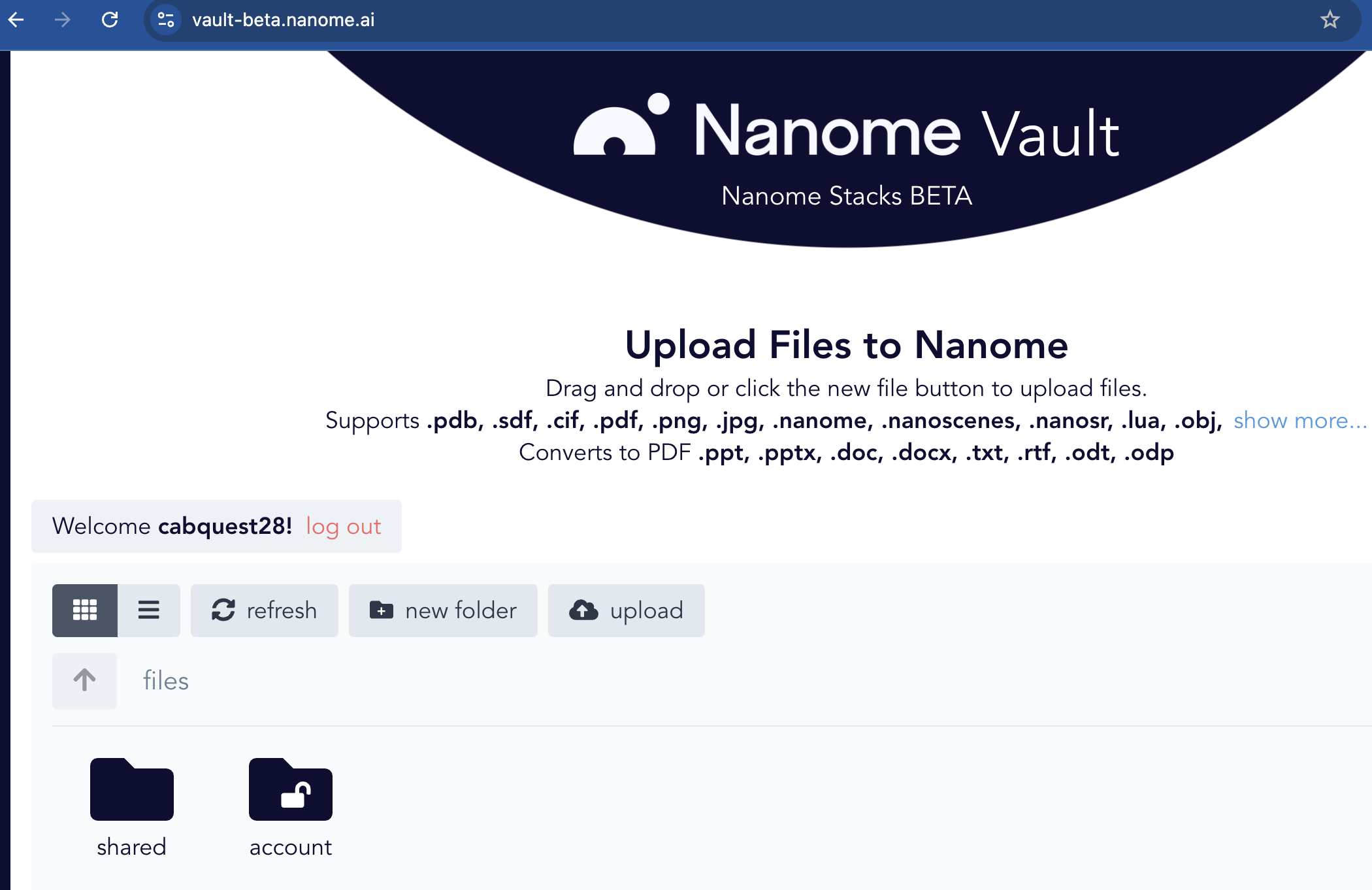


**Figure SF6** How to upload a .pdb file into Nanome Vault online platform.

**S1. Step 4: Save .cif file as .stl for 3D Printing**

In Mercury, you may want to remove any extra molecules such as solvents, by right-clicking on the molecule and selecting **Delete this Molecule**. Optionally, if your molecule has disordered sections where it is showing multiple copies of the same atom, you can simplify the molecule for 3D printing by selecting a specific assembly (A or B) in the **Disorder** section of the top Toolbar. This will show only the one set of atoms. Once you have removed any extra items that you do not wish to include in the 3D printed molecule, in the toolbar, from the **Style** dropdown, select **Ball and Stick**, and from the **Manage styles…** dropdown, select **3D Print**, then select **File > Print in 3D…**. In the dialog box, select **STL (monochrome)** from the **File Format** dropdown, and click **Generate**. This will create a .stl file, and display the file name and location. It will also display the size of the model. Check to make sure that this is the size that you want to print and that it is not too large to be printed (the Bambu X1E can print objects up to 254 x 254 x 254 mm). If you need to adjust the size, change the **Scale** value and click **Generate** again. Once you are satisfied with the output, note the final name and location of the file, and click **Close**.


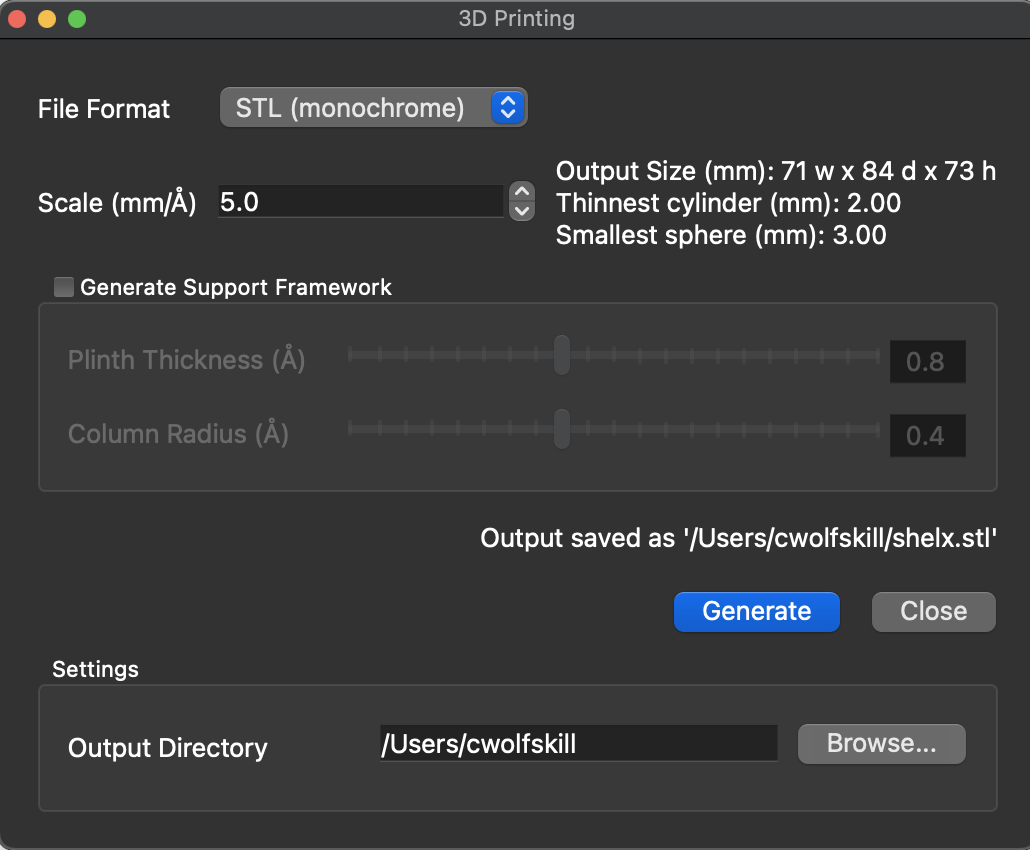


**Figure SF7** How to covert .cif file to .stl file in Mercury.

**S2. Part II: 3D Printing**

**S2.1. Preparation of the .stl file for printing**

Printing the structures starts with opening the Bambu Studio software, and ensuring that it is logged in to the account that is associated with the Bambu 3D printer that you will be using. Go to **File > Import > Import 3MF/STL/STEP/SVG/OBJ/AMF…** and select the STL file that you exported from Mercury.

**S2. Step 2.1: Prep the File for Printing**Open the Bambu Studio software, and ensure that it is logged in to the account that is associated with the Bambu 3D printer that you will be using. Go to **File > Import > Import 3MF/STL/STEP/SVG/OBJ/AMF…** and select the STL file that you exported from Mercury.


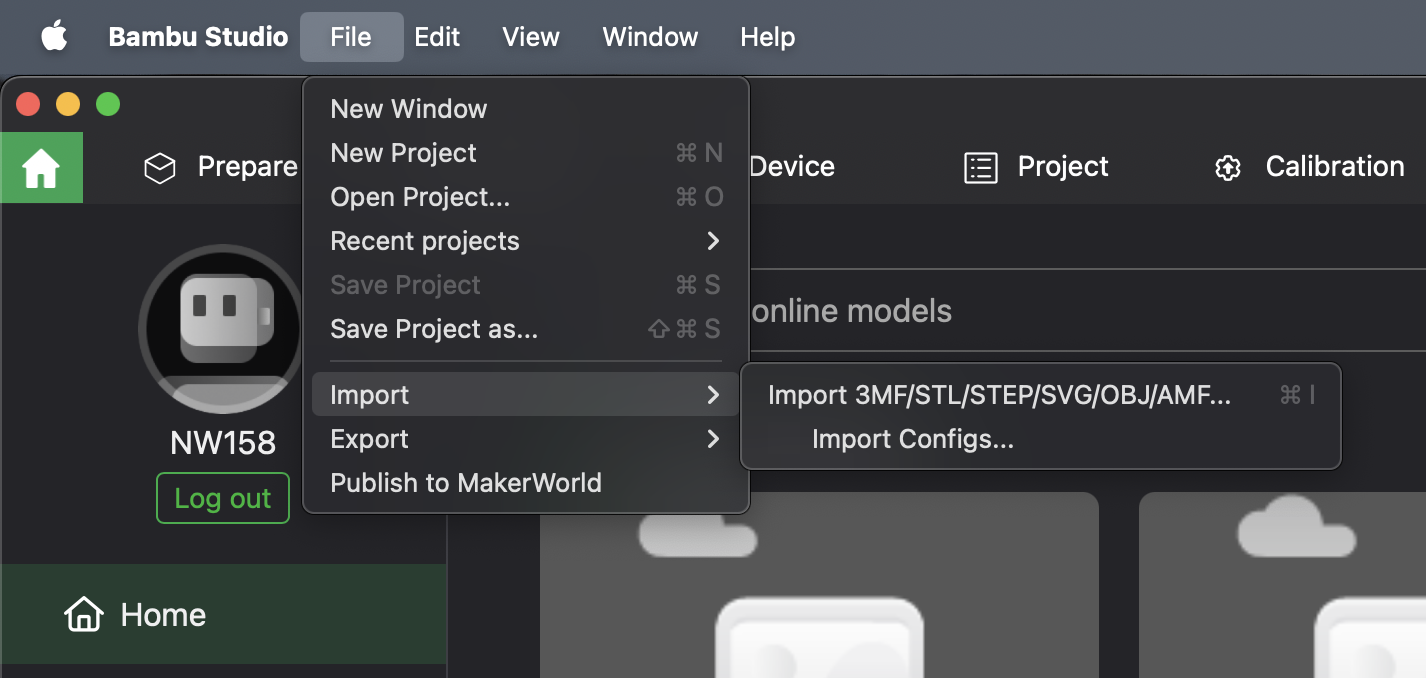


**Figure SF8** How to upload .stl file to Bambu Studio software.

This will switch to the **Prepare** tab with your molecule imported onto the build plate. From the toolbar, click on the Auto Orient icon to allow Bambu to choose how to lay the molecule for printing. Optionally, you can select the **Lay on Face** icon to manually choose how to orient the molecule (it will show the various faces in the molecule and you can click on which one you would like to face down on the build plate).

**S2. Step 2.2: Slice the Model**

The model will need to be sliced in order to compute the trajectory that the 3D printer will use to print. In the first dropdown under **Project Filaments**, ensure that **Generic PLA** is selected (this is the type of filament we will be using to print the model). There are many other settings at the bottom of the left pane that can be adjusted to finetune the printing process. To print these molecules, we will need to configure the printer to create support. Since there are many sections of the model that are floating without anything underneath them use a slightly smaller layer height and slower speed for printing. A preset that already has all the adjusted settings has been saved. In the dropdown in the left pane under **Process**, select the User Preset called **0.16mm with Support – Molecules** (Figure S9) to apply all these settings. Then click **Slice Plate** in the upper right (Figure S10). It will automatically jump to the **Preview** tab and should say **Print OK** in the bottom right. (If it gives you errors or warnings at this stage, it will probably still let you try to print, but it is not likely to work well. So, it would be better to determine why your model was not able to be sliced properly before you try to print it.)


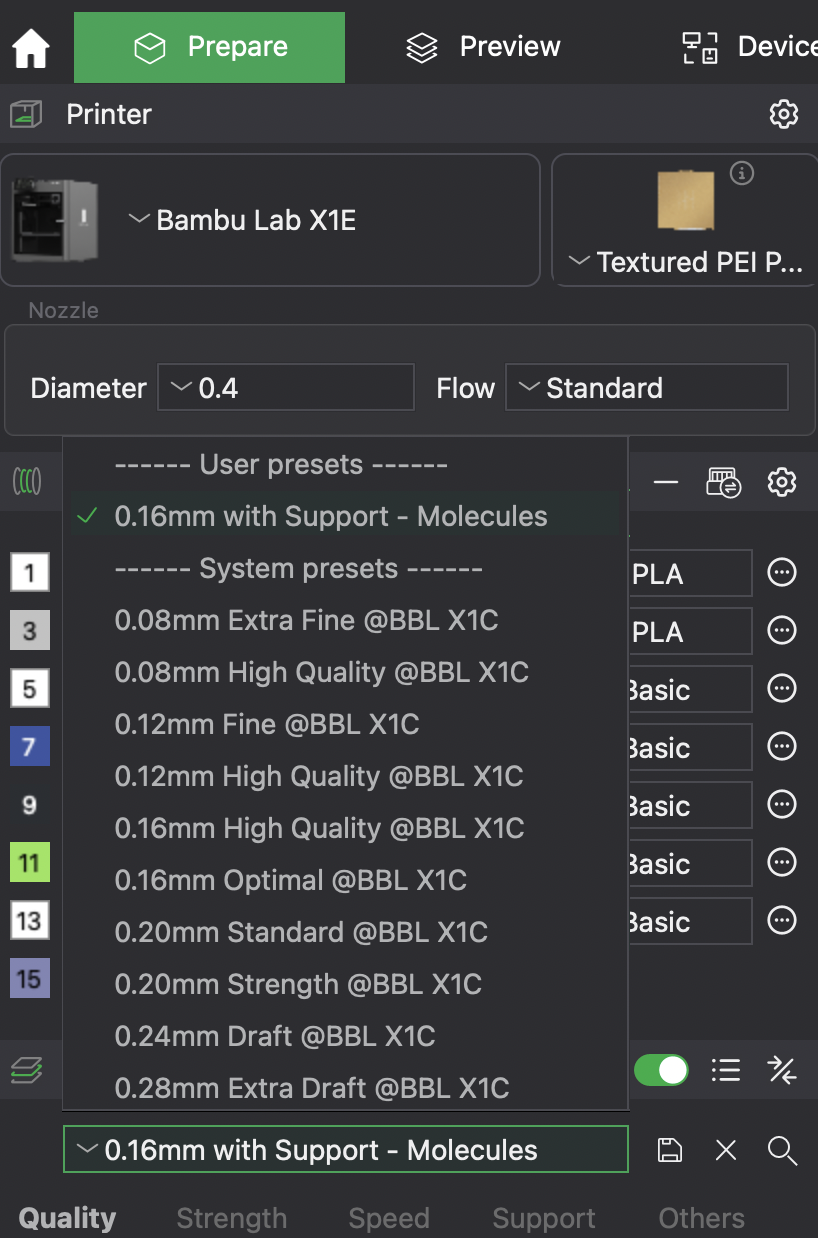


**Figure SF9** How to prepare .stl file to printing in Bambu Studio software.


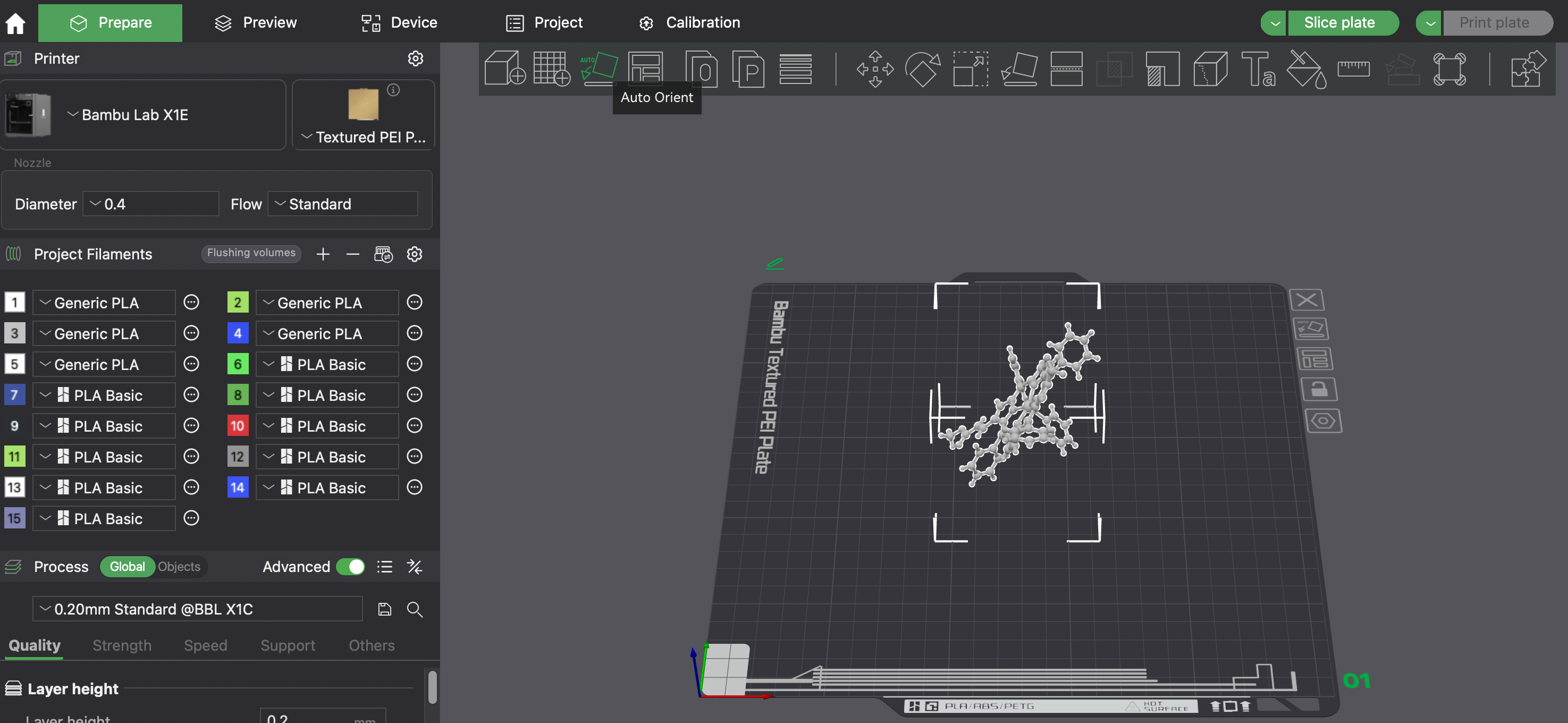


**Figure SF10** How to prepare, slice, and print .stl file to printing in Bambu Studio software.

**S2. Step 2.3: Sending the File to the Bambu 3D printer**

The job can be sent to the Bambu 3D printer by clicking Print Plate (Figure S10 top right). It will automatically try to select the right filament for you, but you can also change which filament is being used for this print by clicking in the box that says PLA under the model and selecting a different slot for it to use. Click Send to start the print. It is recommended to leave the door to the printer ajar while printing to allow the space to ventilate and not build up too much heat, which could cause the extruder to clog.

**S2. Step 2.4: Remove Support and Paint the Atoms**

Once the print has completed, the build plate with the model from the printer and gently bend the plate to remove the model. The built model will contain both the molecule and the additional support that was printed underneath floating sections of the molecule (Figure S11). Carefully break the support pieces off so that just the printed molecule remains. Then, use nail polish to colour the atoms in the molecule according to the CPK colour convention shown in the table below.

**
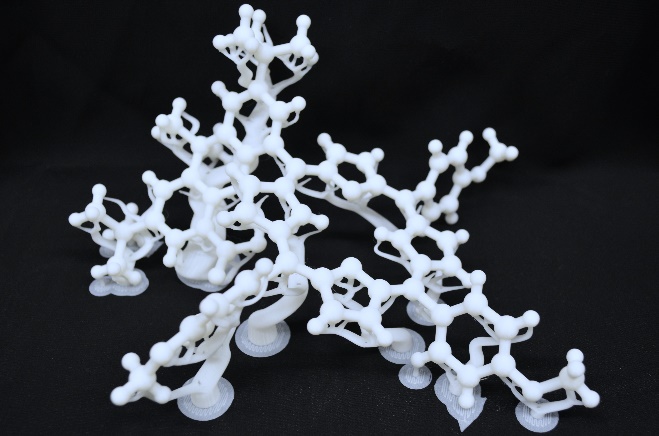

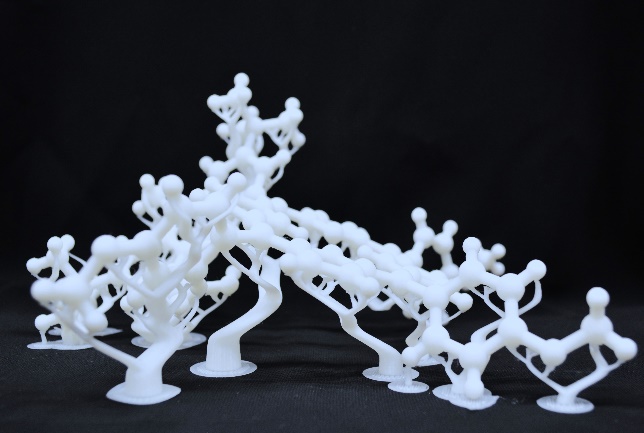
**

**B**

**A**

**Figure SF11** The built model of the molecule with the support. Side view (A), front view(B).

| **Element** | **Colour** |
| --- | --- |
| hydrogen | white |
| carbon | grey |
| nitrogen | dark blue |
| oxygen | red |
| chlorine | green |
| fluorine | light green |
| iron | dark orange |
| palladium* | yellow |
| platinum* | steel blue |
| magnesium* | purple |

**Table S1** Corey, Pauling, and Koltun (CPK) colour convention. *Authors’ choice of colour.

Colour convention of the molecules printed for this study.

**S3. Viewing the Molecule in Nanome VR**

**S3. Step 1. Introduction to the VR Headset and Controllers**

In this module Meta Quest 3 VR headsets and controllers were used. The headsets are standalone devices, and they do not need to be connected to a computer to run. Additionally, the headsets already have the Nanome application loaded on them, which will be used to view molecules.

The two controllers allow the hands to be used in virtual reality. As the handsets are moved around, the the hands of the avatar will move at the same time, and there will be a line from each hand to where it is pointing in VR platform. The primary buttons on the controllers that will be used are; the trigger, the grip, and the joystick. The trigger button (under index finger) can be used to select items or click buttons that one is pointing to. The grip button (under middle finger) can be used to grip items in order to move the molecules around in the VR platrom. The joystick (or thumbstick, under thumb) can be used to cycle through menus (Figure S12).


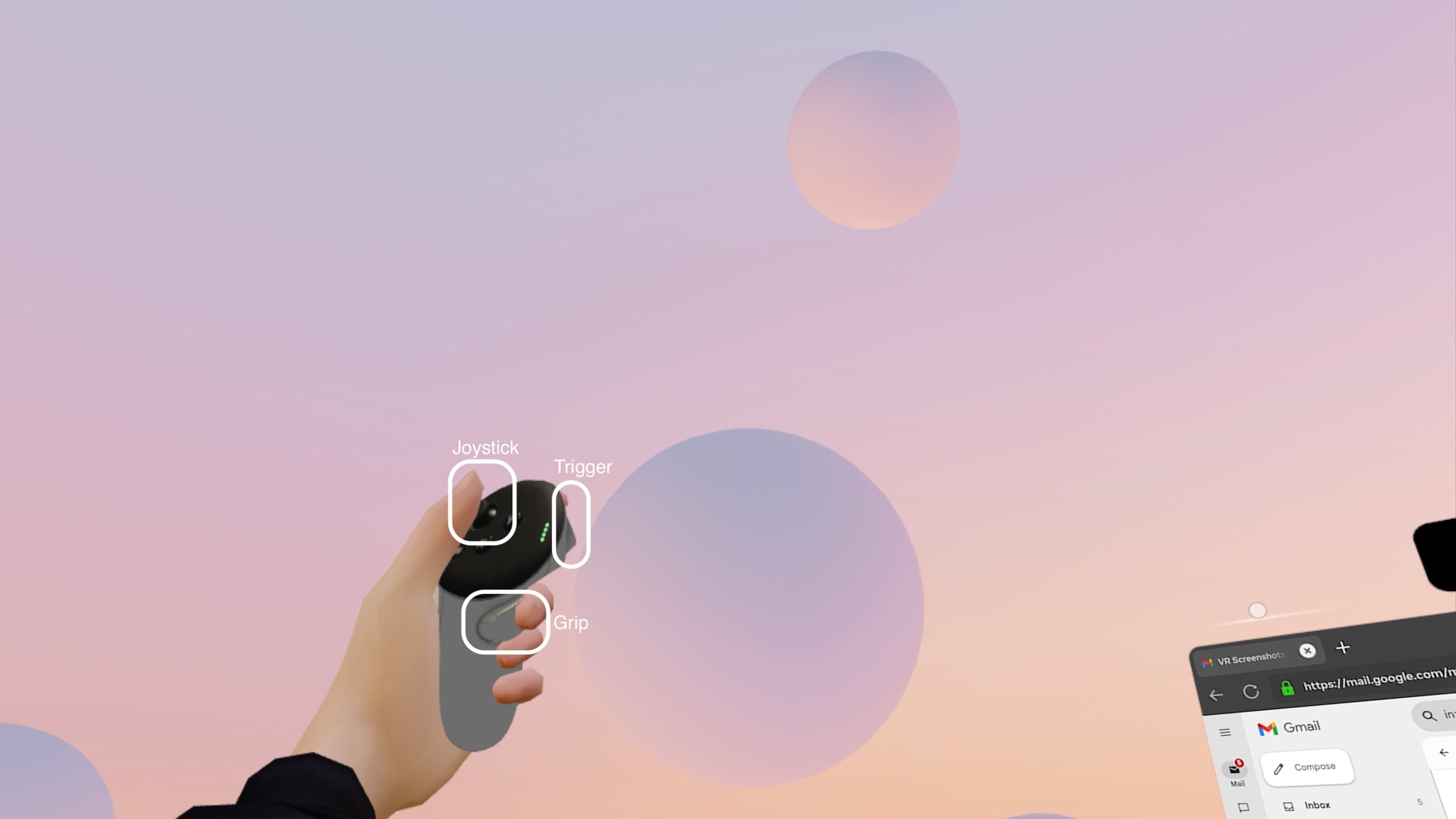


**Figure SF12** The buttons on the controllers.

**S3. Step 2. Opening the Nanome Application using the Virtual Reality Headsets**

After putting on the VR headset, choose from the **Library** (the icon on the right side of the bottom menu bar) the **Nanome** application. The hand controllers need to be used to point and the trigger button to select items (Figure S13).


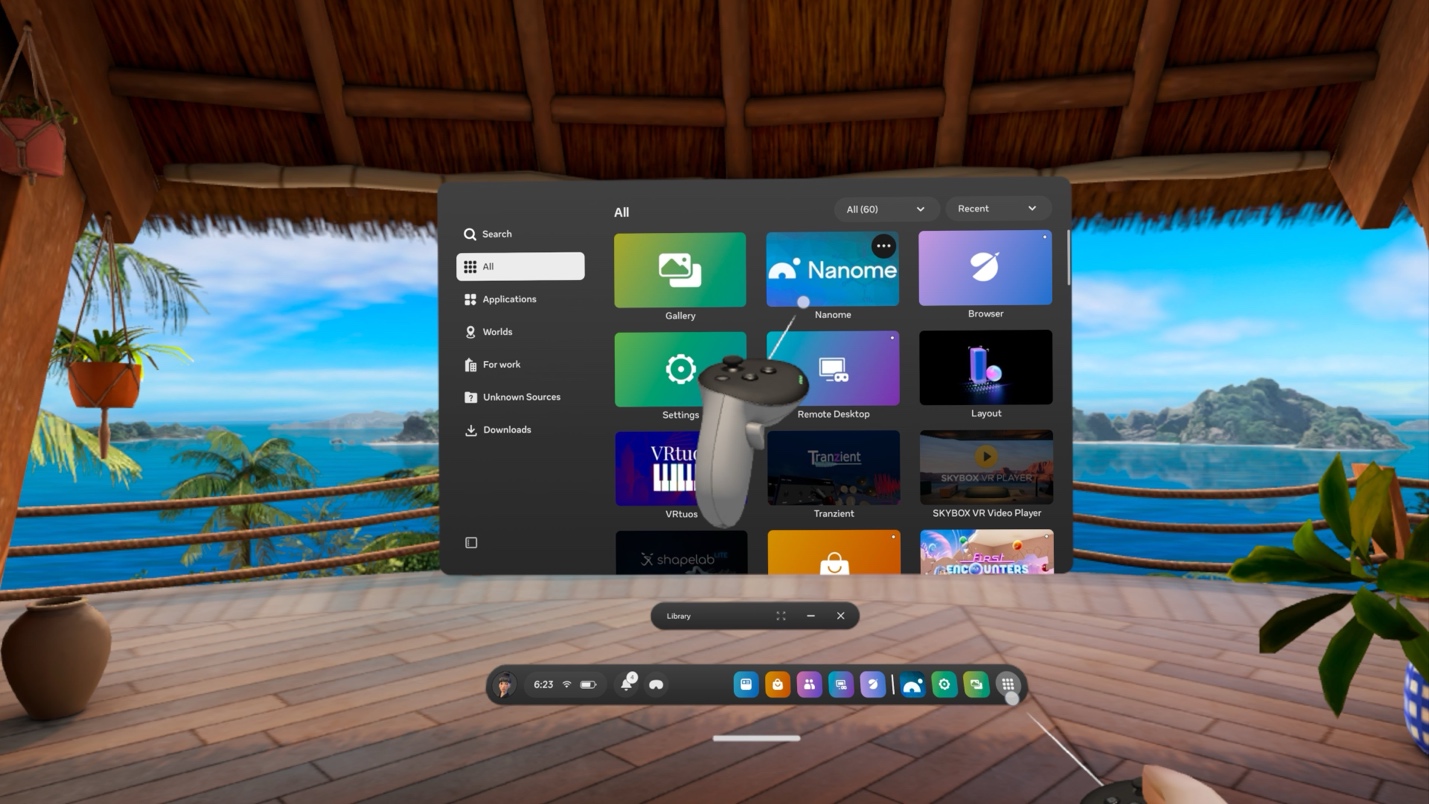


**Figure SF13** How to choose Nanome application from the Library settings using VR headsets.

In the next step, the sign in ] for Nanome will open. the Nanome username and password can be used to log in the site. These are the same credentials created to access the Vault and to upload the .pdb files (S1.1.1, Step 3, Figure S4-S6) which will take the viewer to **the Lobby**. From the Lobby Nanome training materials can be accessed, settings can be changed, and an existing multi-user room could be joined, or a new room can be started (Figure S14). In this space (1) loading, (ii) viewing the molecule of choice, (iii) starting a new room by selecting the **Create Room** button, and (iv) starting a new room by clicking **Start Public Room**. (entering a password is not necessary) can be done. These applications will open a new workspace. If **Quick Drop** option was used (S1.1.1., Figure S5, S6), the molecule of choice should be seen in the 3D in the room as well as the name of the molecule in the **Entry List** on the main window (Figure S15).


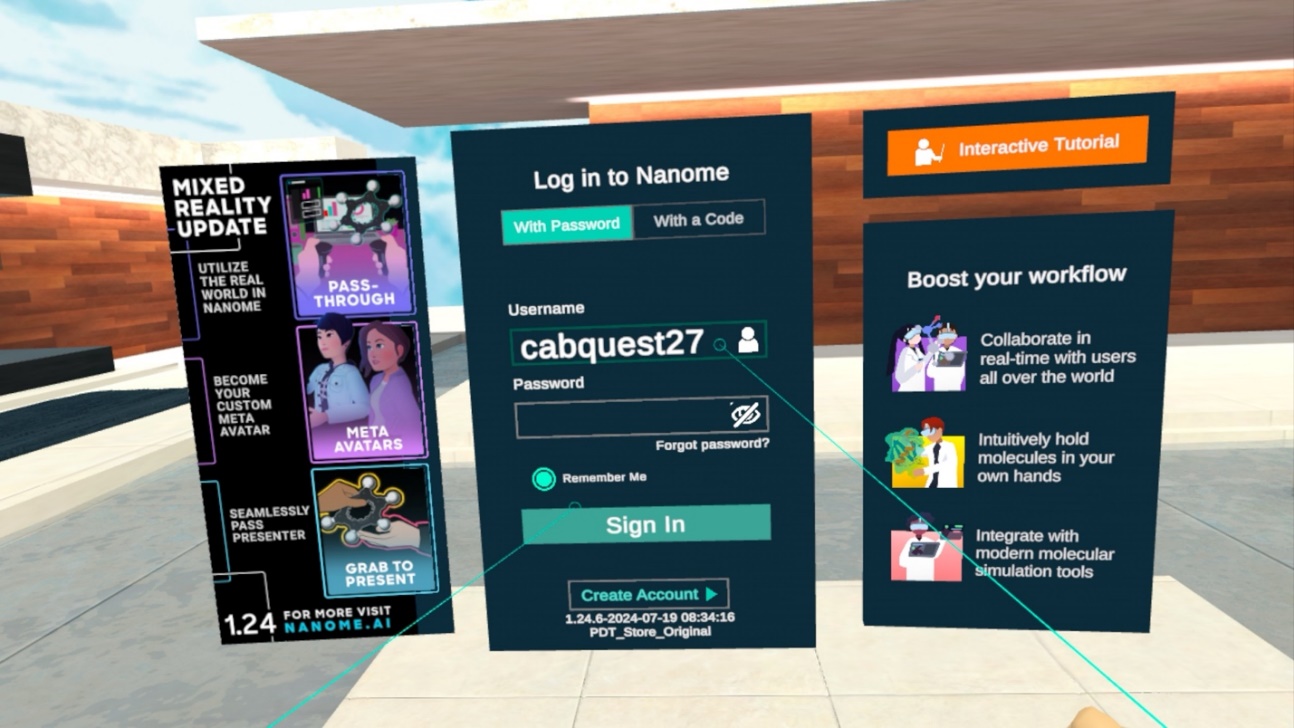


**Figure SF14** How to enter the Nanome username and password.


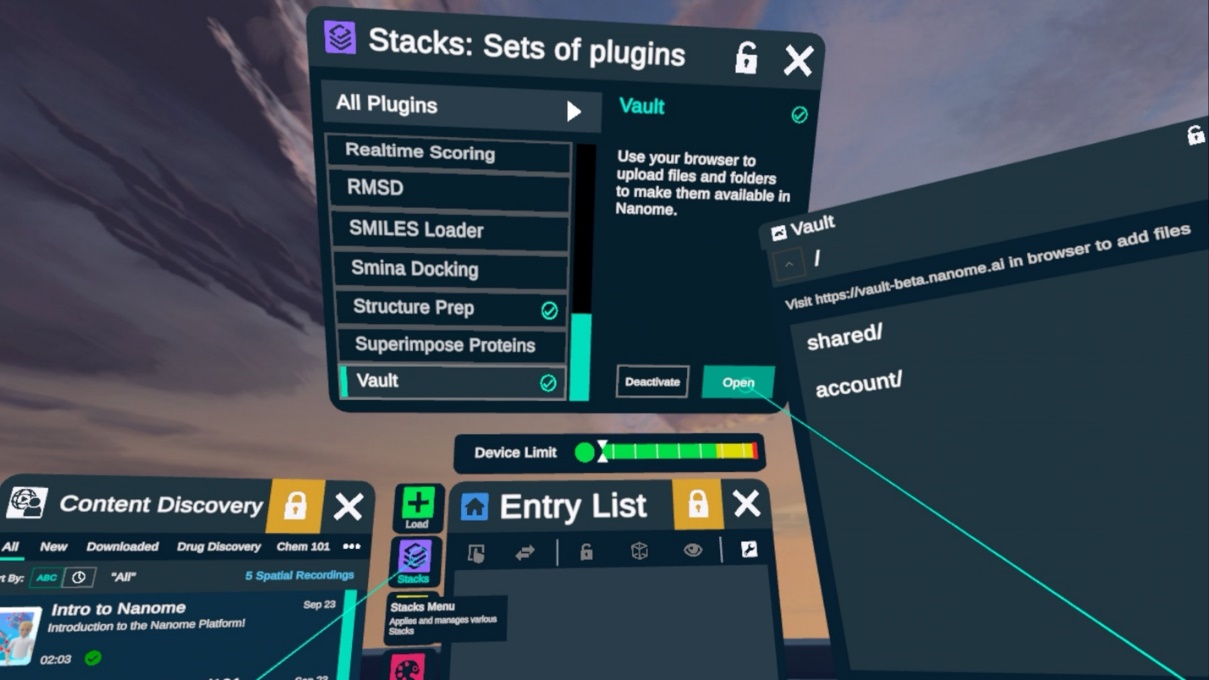


**Figure SF15** How to download .pdf file using Entry List →Vault→Open→Shared→.pdb

**S3. Step 3. Manipulating the Molecule in Nanome VR**

Under the **Entry List** (Figure S15), the choice of the molecule can be selected by using the tricker button on which will then highlight the molecule in the list and move the molecule to the 3D workspace. The Zoom button next to the molecule name can also be used to bring the molecule in front of the viewer. By clicking and holding the grip button on the controller (under middle finger) the molecule can be grabbed the molecule and moved allowing reposition it or look at it from a different angle.

**
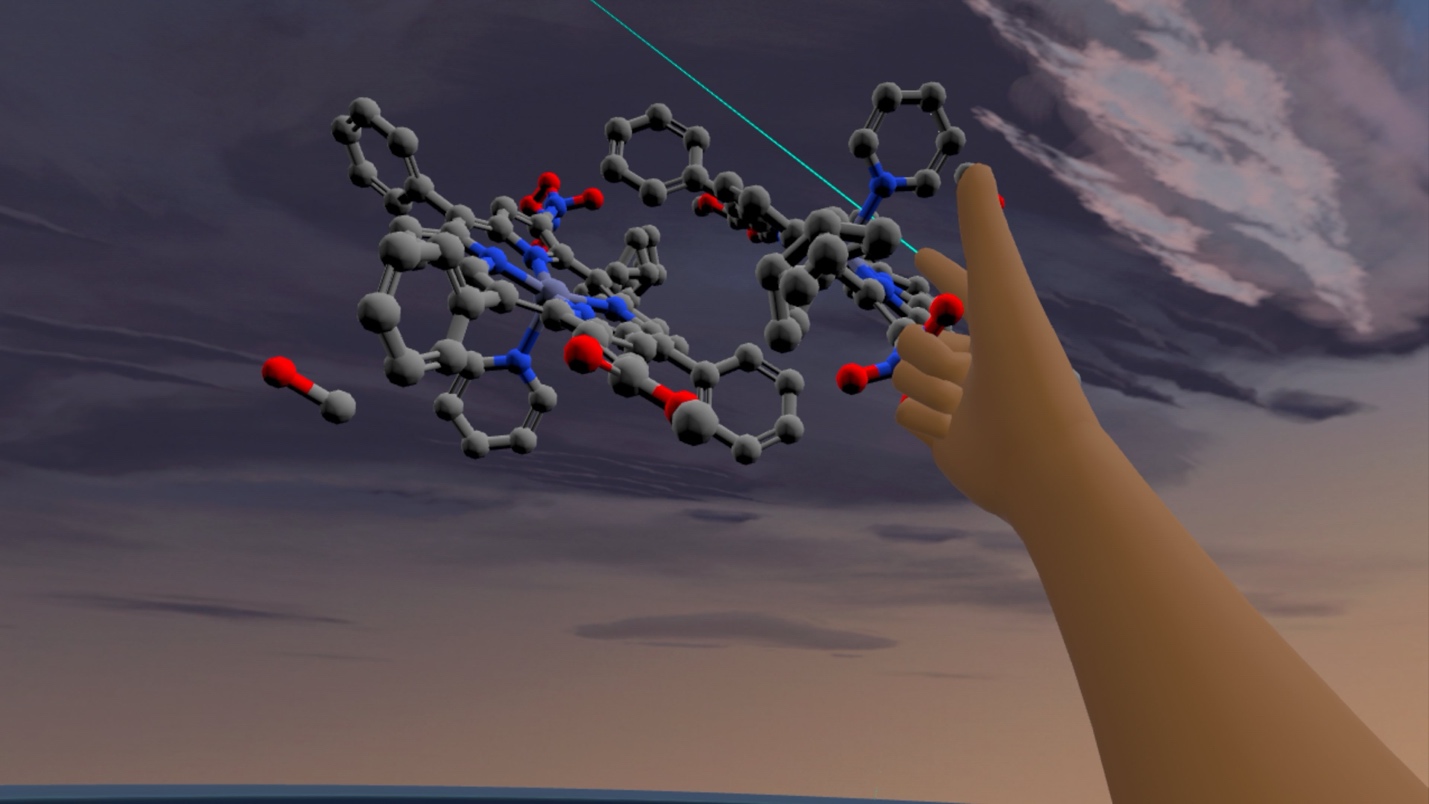
**

**Figure SF16** How to manipulate the molecule in 3D VR platform.

Additionally, the molecule can be grabbed with both virtual hands (using the grip buttons on both controllers simultaneously), shrunk or enlarged by moving the hands closer together or further apart. The molecule can also be brought closer or placed further away from the viewer by grabbing the structure using the trigger button and while the joystick button can be pressed down to move the molecule to backwards and forwards on the virtual 3D space. These same actions can be used to grab and reposition the various windows in Nanome (Figure S17).


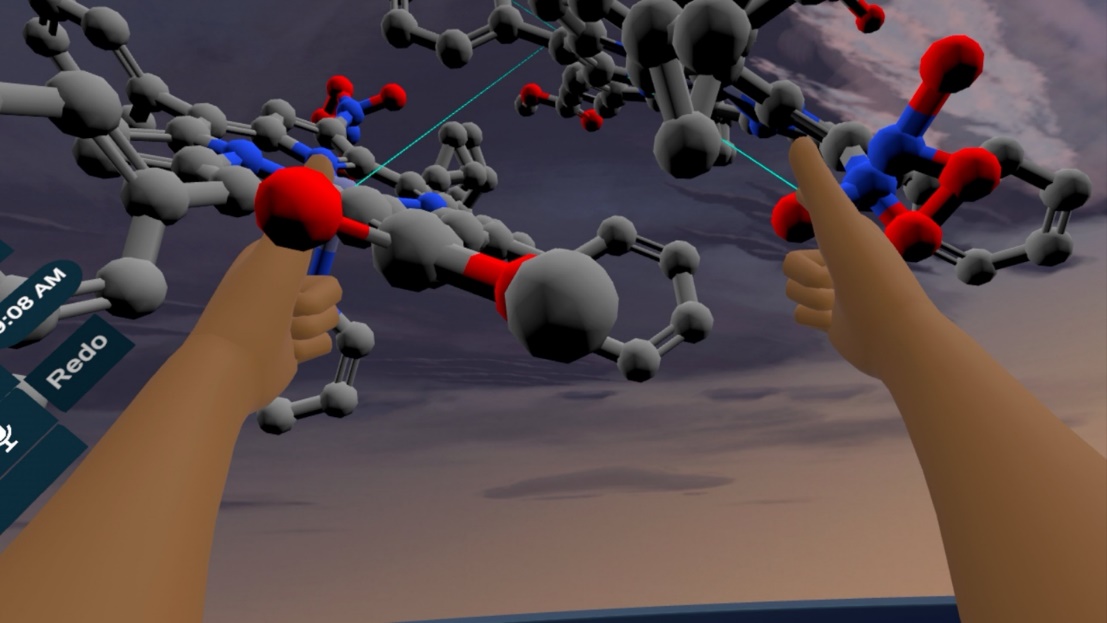


**Figure SF17** How to manipulate shrink, enlarge, move backwards, and forwards the molecule in 3D VR platform.

At any point of this virtual experience the real world behind the molecule can be viewed by (1) using the trigger button to select **Settings** from the menu next to the left forearm of the viewer, (2) selecting the **Mixed Reality** option.


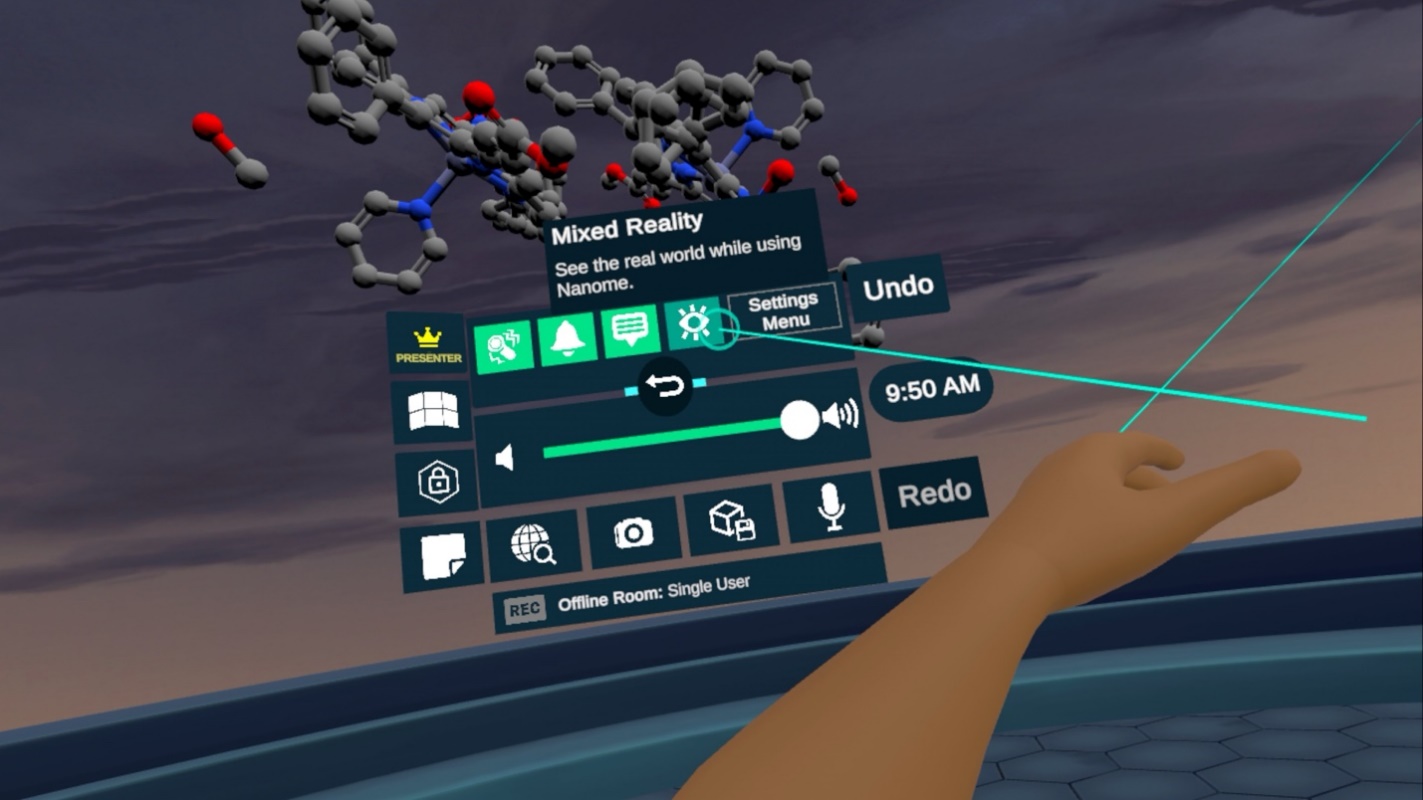


**Figure SF18** How to turn on or off the external video view in 3D Virtual Reality room by using the menu available on the left form arm of the viewer.

**S4. Analyzing the Molecule in Mercury and Nanome VR**

**S4. Step 1: Structural Information**

To obtain structural information in VR, first in Mercury the following steps needs to be followed; **Display > More Information > Structure Information** (Figure SF19).


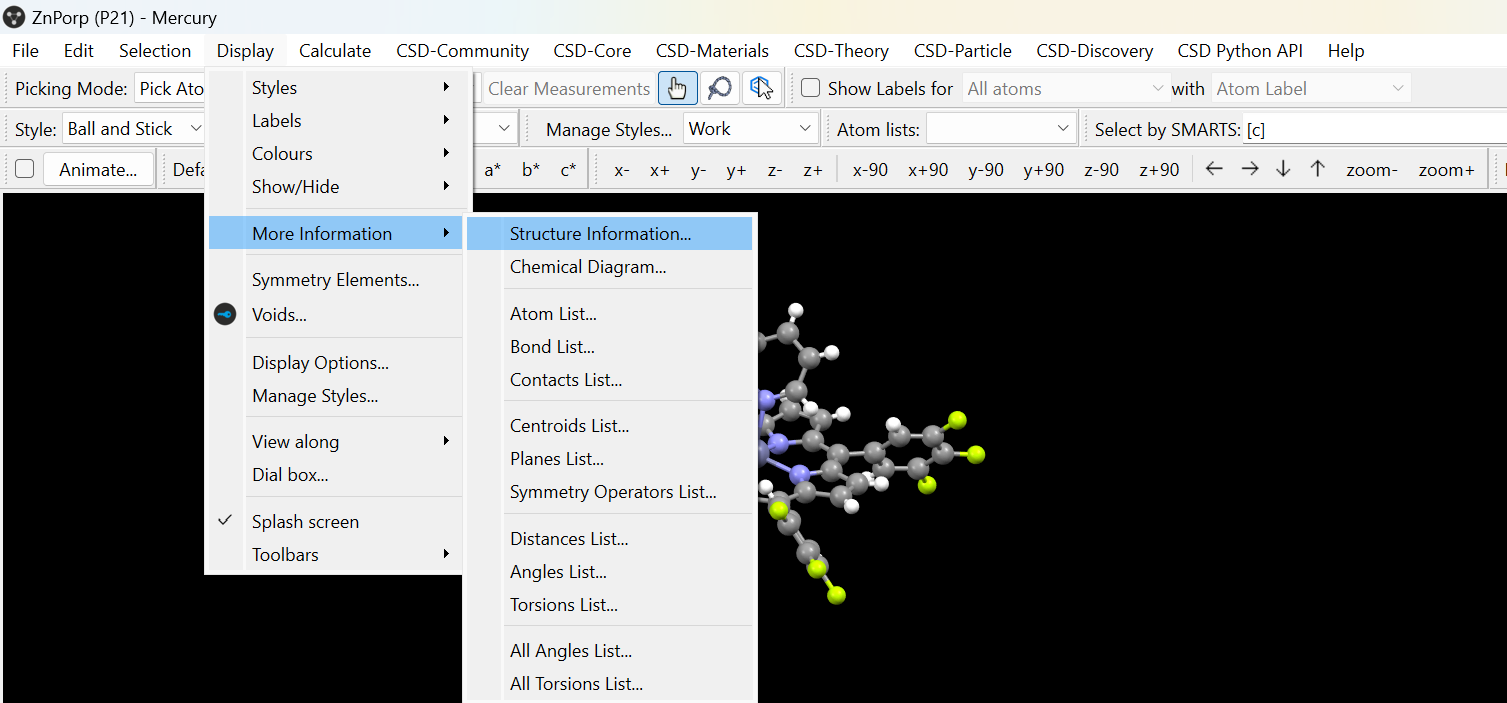


**Figure SF19** How to turn on or off the external video view in 3D Virtual Reality room by using the menu available on the left form arm of the viewer.

The **Structure Information** tab provides information about the space group, the cell lengths and also verifies the molecule’s space groups, such as that it is P2_1_, besides the cell lengths and the angles (Figure S20).


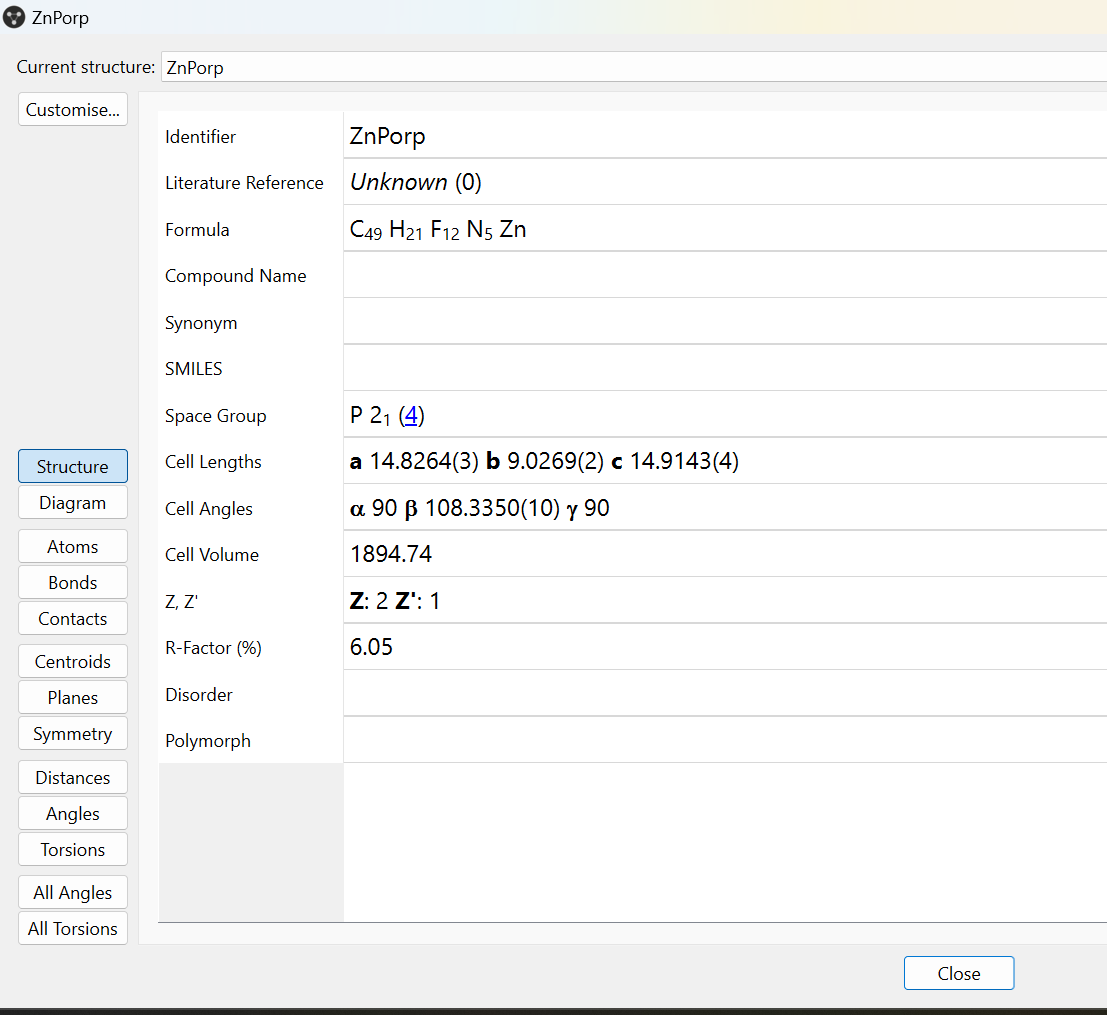


0

**Figure SF20** How to obtain **Structure Information** about the molecule in Mercury using the .cif file.

**S4. Step 2:** **Measuring Bond Lengths**

The bond length of the molecule can be measured in Mercury software by clicking on **Bonds** from the menu on the left of the structural information window (Figure S20). The bond lengths in Å (angstrom) can be found listed under this menu. The atom numbering on the molecule can be accessed through the **Atoms** option**.**


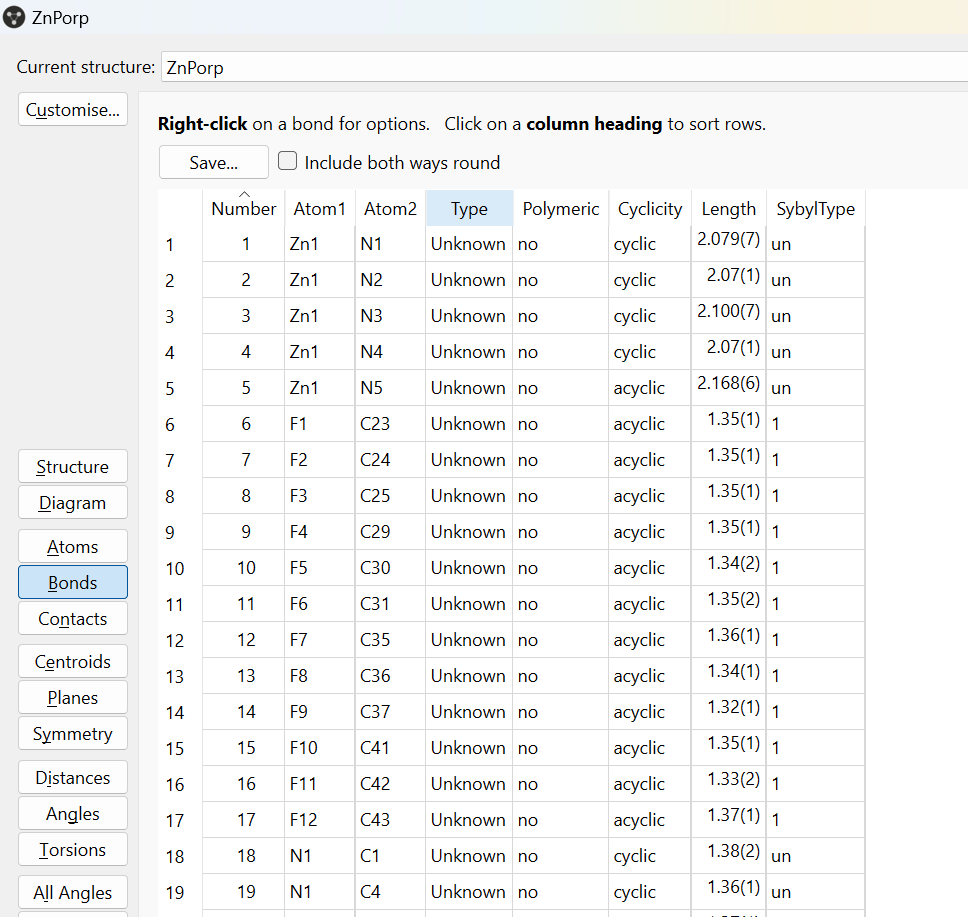


**Figure SF21** How to obtain the bonds lengths of the molecule in Mercury using the .cif file.

**S4. Step 3. Structural Information through in the VR platform-How to measure distances between atoms.**

The structural information about the molecule in VR can be obtained through **Nanome**. The **Modify** window provides the **Tools** menu where multiple options are provided to make manipulations on the molecule. All the options provided through **MEASURE** menu can be accessed by pressing to the left and right on the joystick of the hand controller. This motion will also cycle through the various measuring tools; **MEASURE: Distance**, **MEASURE: Angle**, **MEASURE: Tortional Angle**, **MEASURE: Delete**, etc. For example, to measure the distance between two atoms on the molecule of choice, **MEASURE: Distance** tool should be utilized. After selecting the two atoms whose bond length wished to be measured, simply moving the controller close to the first atom, until it is highlighted in blue, and then clicking the trigger button will highlight the selected atom in yellow. After selecting the second atom, both atoms will be highlighted in green, and the distance between them in angstroms will be displayed (Figure S22).


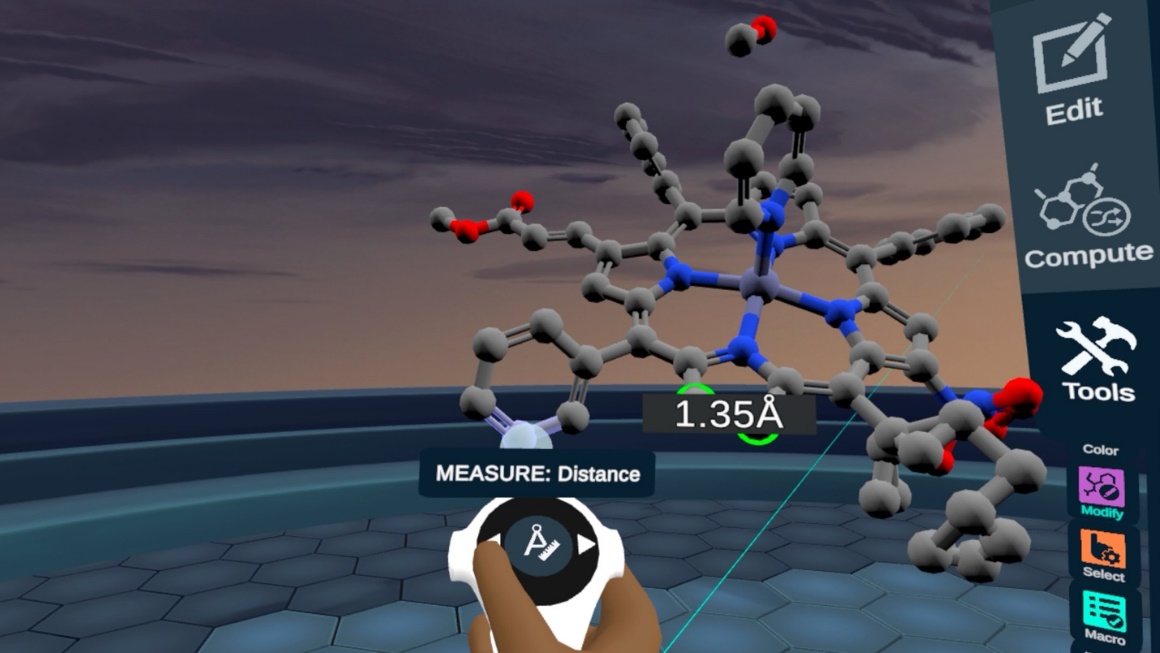


**Figure SF22** How to measure bond lengths between the atoms on the molecule using VR sets in the 3D platform.

**S4. Step 4. Structural Information in the VR platform- How to measure angles.**

The angles of the molecule can be obtained in Mercury software by clicking on **All Angles** from the menu on the left of the structural information window (Figure S23).


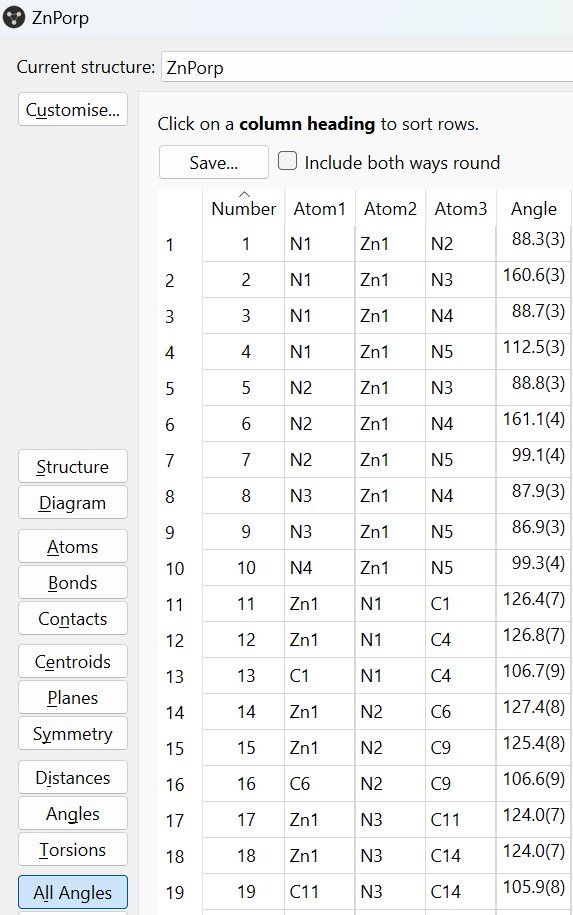


**Figure SF23** How to obtain **All Angles** of the molecule in Mercury using the .cif file.

**S4. Step 5. Structural Information in the VR platform- How to measure angles.**

To measure the angles on the molecule of choice, **MEASURE: Angle** tool should be utilized. After selecting the three atoms whose bond angles wished to be measured, simply moving the controller close to the first atom, until it is highlighted in blue, and then clicking the second and the third atom using the trigger button will highlight these selected atoms. After all atoms are highlighted in green, the angle between them in angstroms will be displayed (Figure S24).


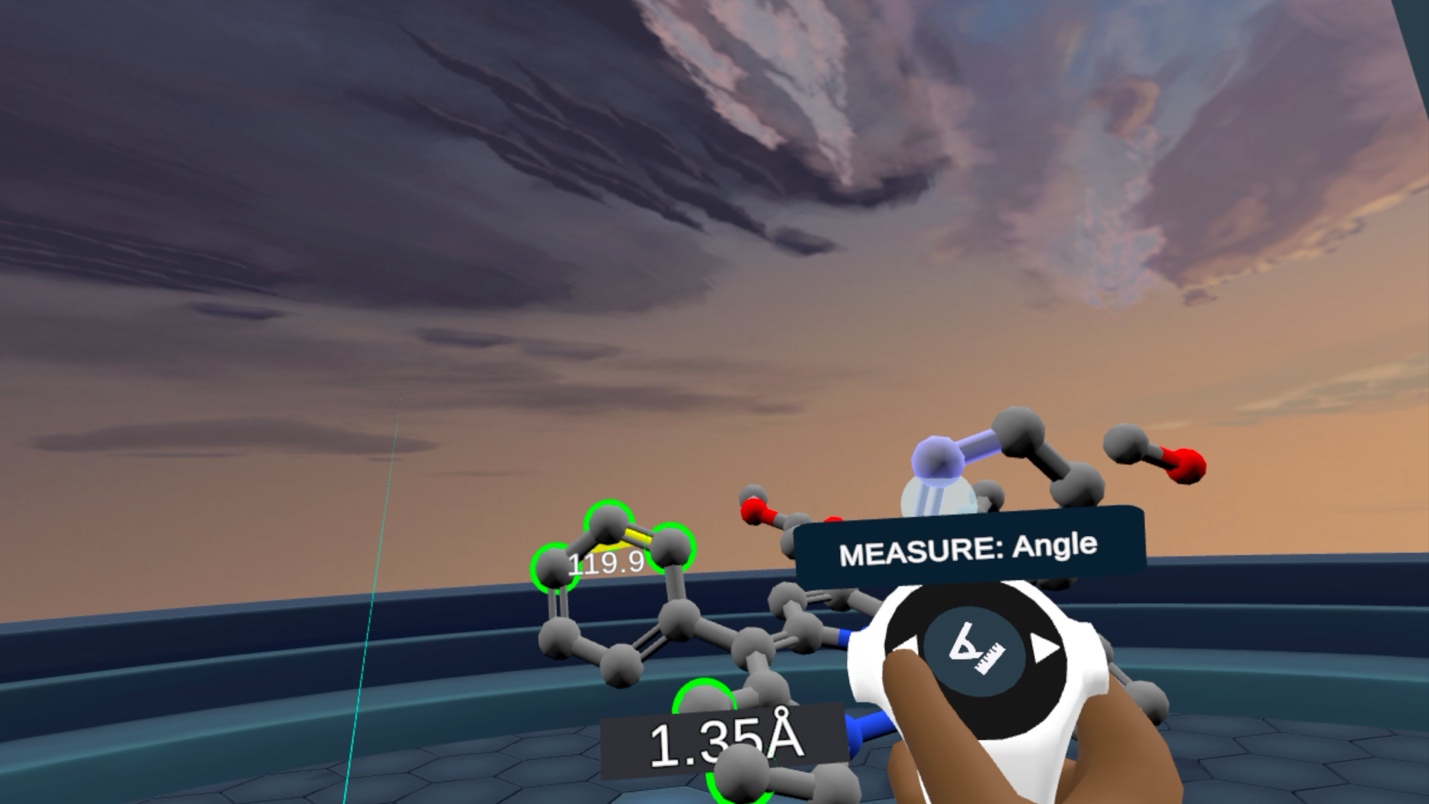


**Figure SF24** How to measure bond angles on the molecule using VR sets in the 3D platform.

**S4. Step 5. Structural Information in the VR platform- How to measure tortional angles.**

The tortional angles of the molecule can be obtained in Mercury software by clicking on **All Torsions** from the menu on the left of the structural information window (Figure S25).


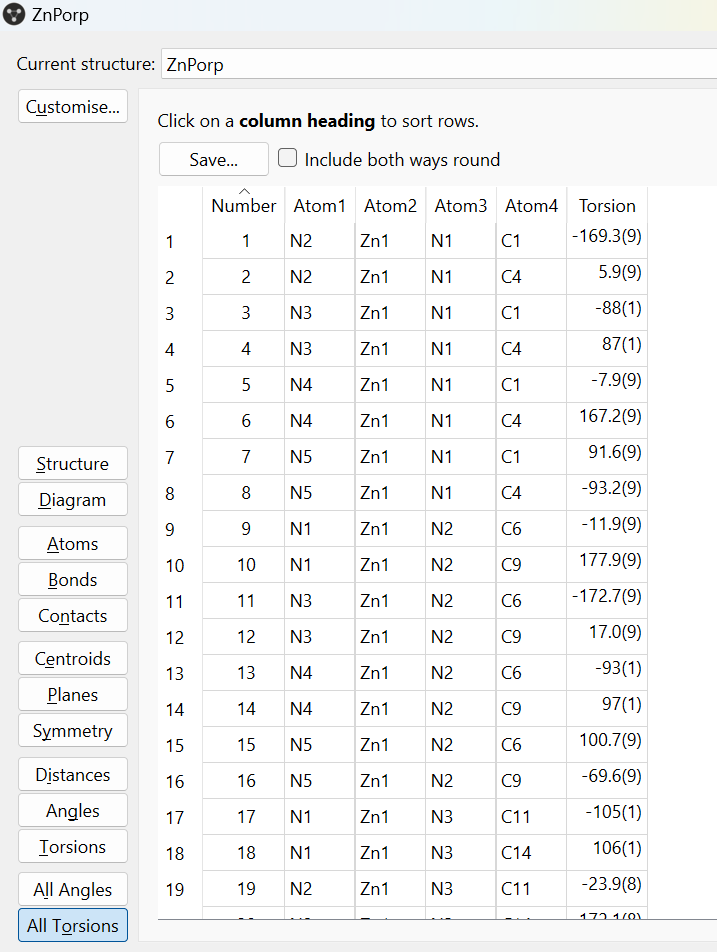


**Figure SF25** How to obtain **All Torsions** of the molecule in Mercury using the .cif file.

To measure the tortional angles on the molecule of choice, **MEASURE: Dihedral** tool should be utilized. After selecting the four atoms whose bond angles wished to be measured, simply moving the controller close to the first atom, until it is highlighted in blue, and then clicking the second, the third, and the forth atom using the trigger button will highlight these selected atoms. After all atoms are highlighted in green, the torsional/dihedral angle in angstroms will be displayed (Figure S26).


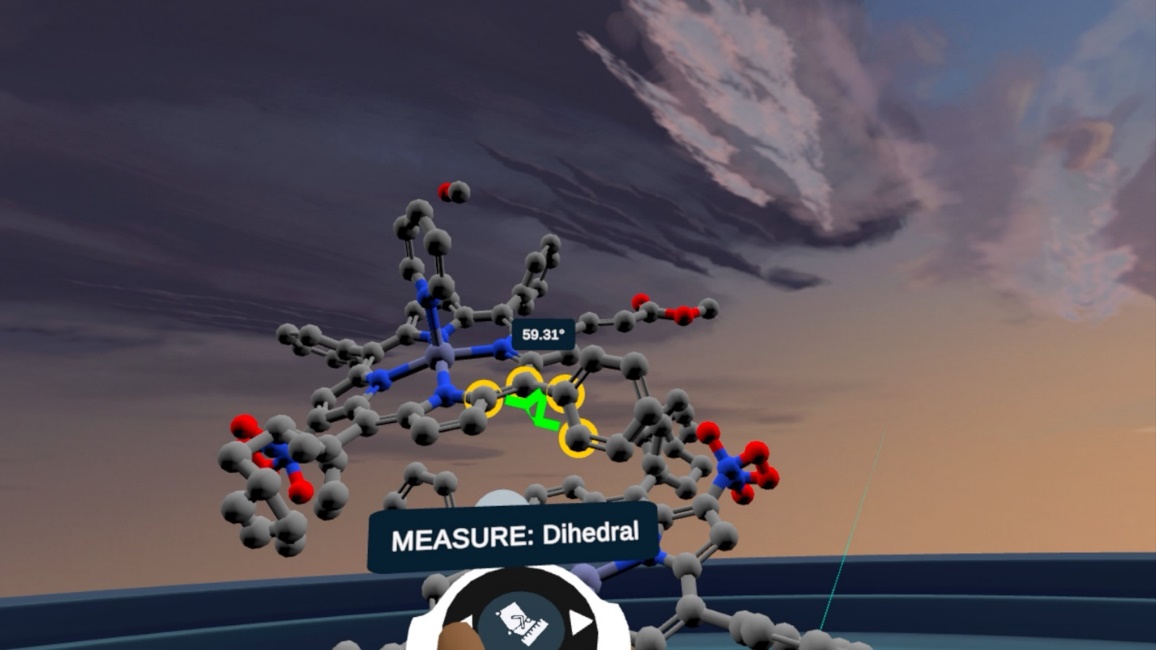


**Figure SF26** How to measure the torsional/dihedral angle on the molecule using VR sets in the 3D platform.

**S4. Step 6. How to Visualize Crystal Packing in the VR platform**

The crystal packing of the molecule can be obtained in Mercury software by (1) selecting C**alculate > Packing/Slicing,** in the **Packing and Slicing** option, (2) checking the box next to **Pack**, and (3) clicking on the **2x2x2** button, (4) clicking on **OK** (Figure S27). Mercury will then show the molecule packed into a crystal structure. To view the packing in VR platform, (1) select **File > Save As** (2) choose the file type from the dropdown menu, (3) select **PDB files**, (4) click **Save**. The same steps in S1. Step 3, Figure S4-S6 can be followed to upload the file using the Nanome Vault and load it into the workspace. The packed molecules can be manipulated, such as measuring angles, bond lengths, tortional angles in the same way as in sections S2, and S3 above (Figure S28).


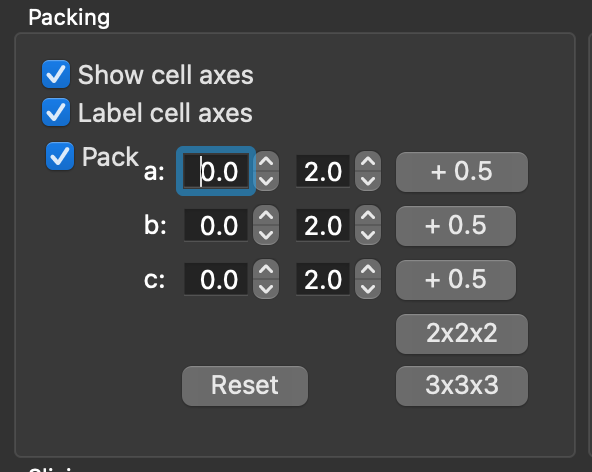


**Figure SF27** How to pack molecules in Mercury.


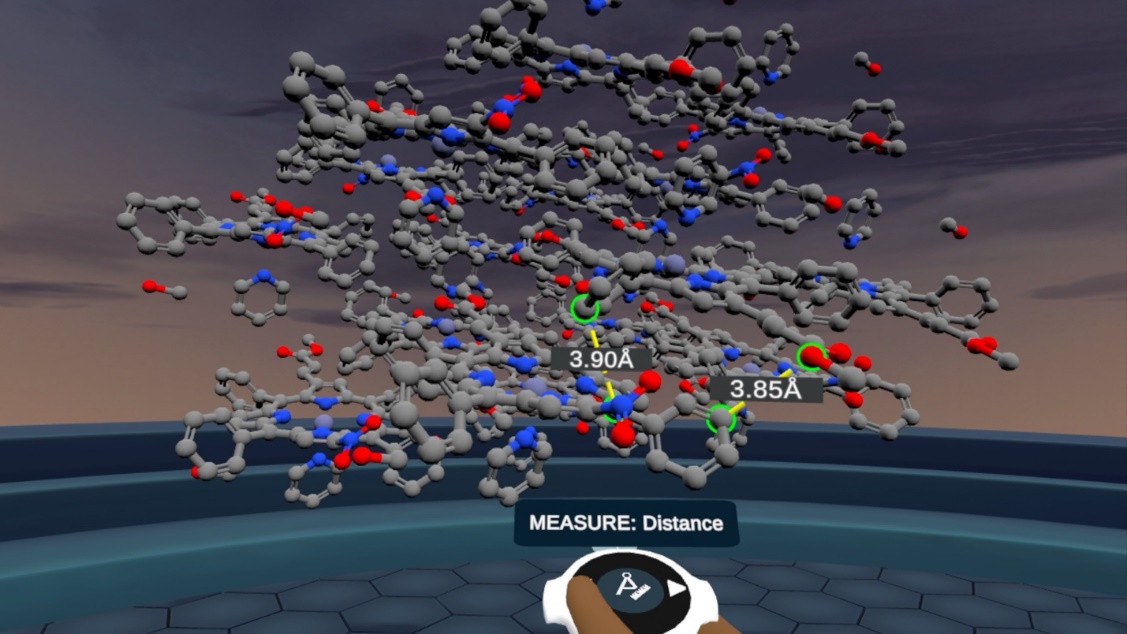


**Figure SF28** Crystal packing view and molecular measurements displayed in the VR platform.

**S4. Step 7. Additional Nanome options in the VR platform- How to delete the measurements done on the molecule.**

To delete measurements in Nanome, the joystick button should be used to cycle through the tools until the **MEASURE: Delete** tool is displayed. Then pointing the controller at the measurement that is wished to delete, and pressing the trigger button will remove the measurement (Figure S27).


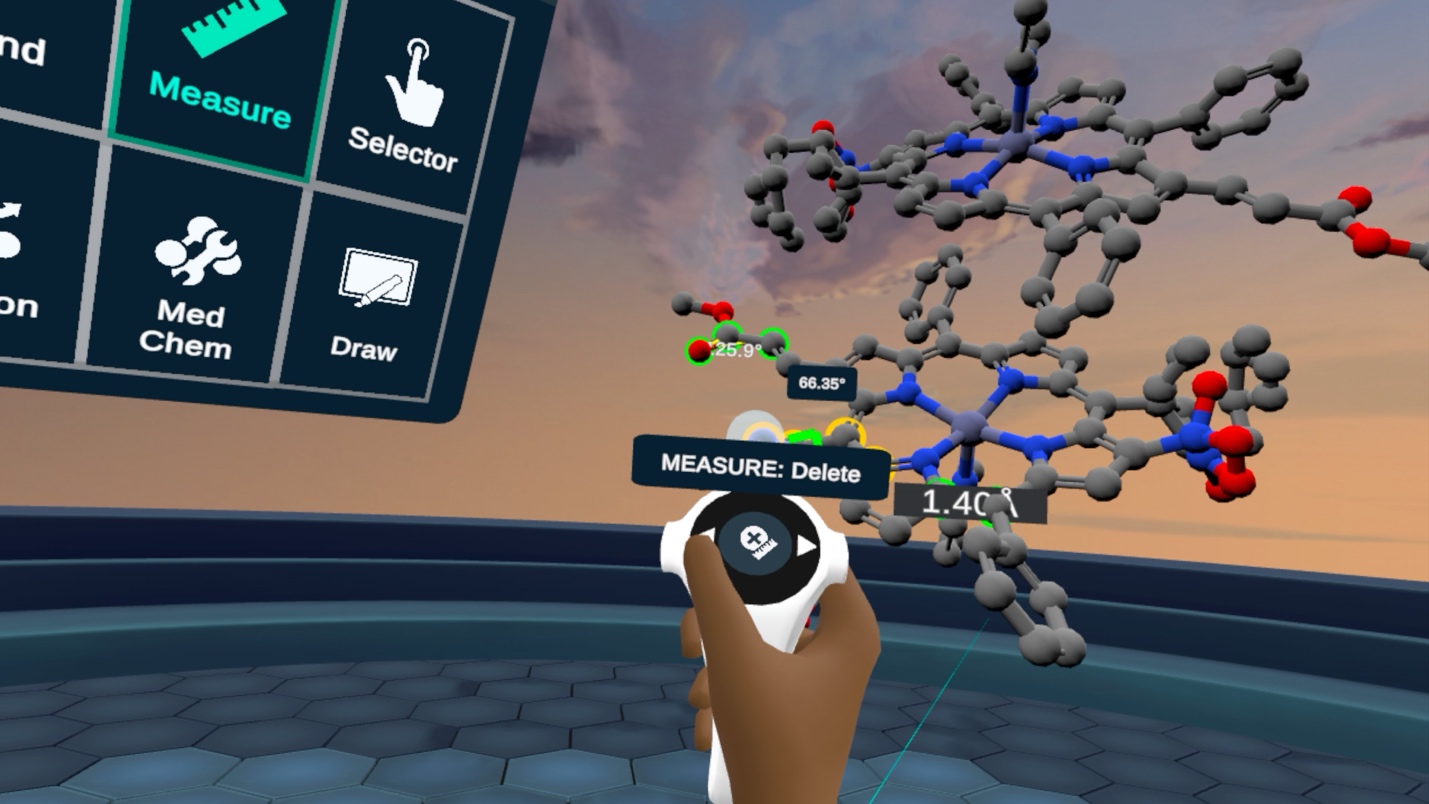


**Figure SF29** How to delete measurements in the 3D platform using the joystick button.

**S4. Step 7. Additional Nanome options in the VR platform- How to take screenshots and email the photos**

~~
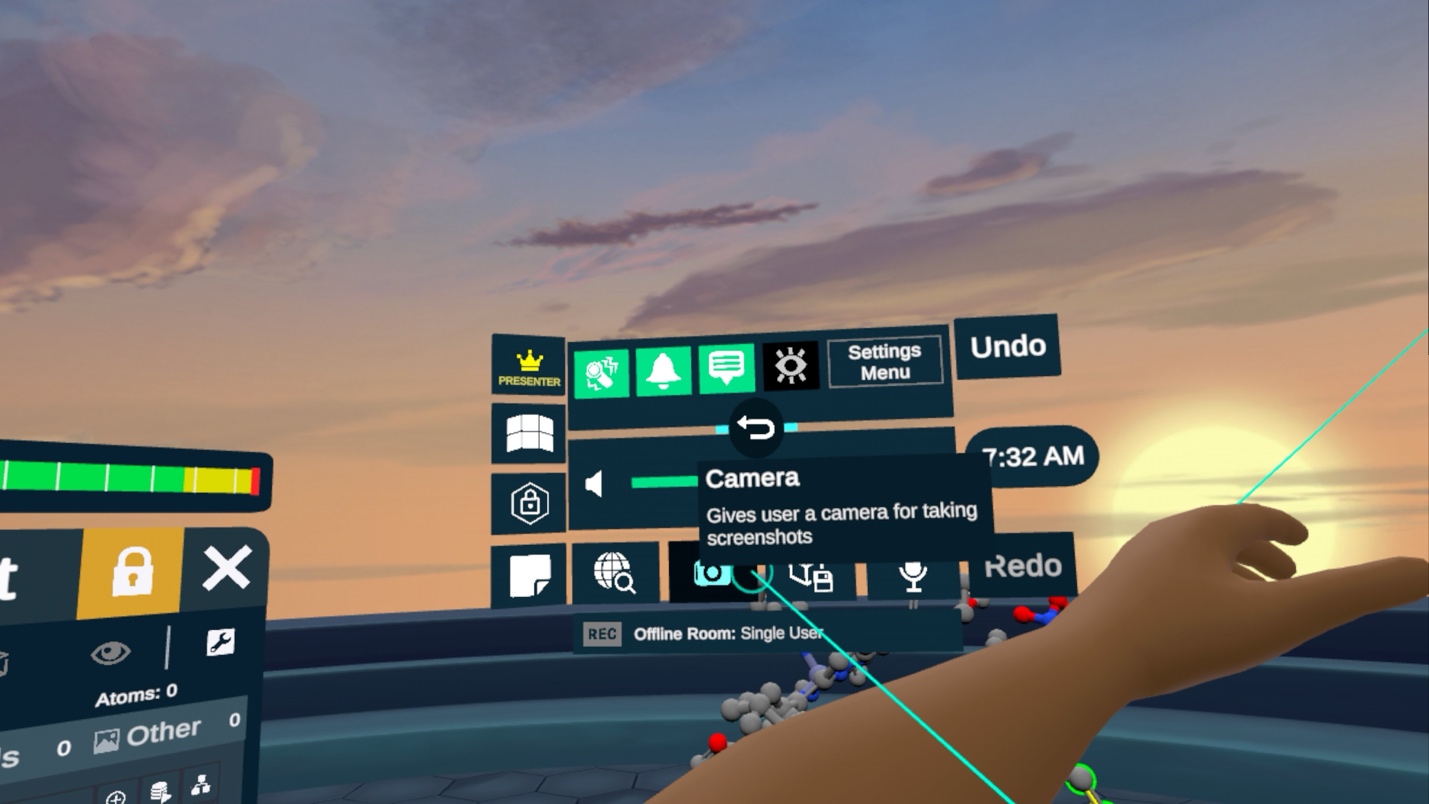
~~Screenshot in **Nanome** can be taken by (1) selecting the **Camera** option from the menu window next to the left forearm of the viewer. This will bring up a window that shows what will be in the screenshot, (2) pressing the Camera button on the right, (3) entering the email address in the **Sharing** window, and (4) pressing the **Send** button (Figures SF28).

**Figure SF30** How to take screenshots in the 3D platform using the trigger button.


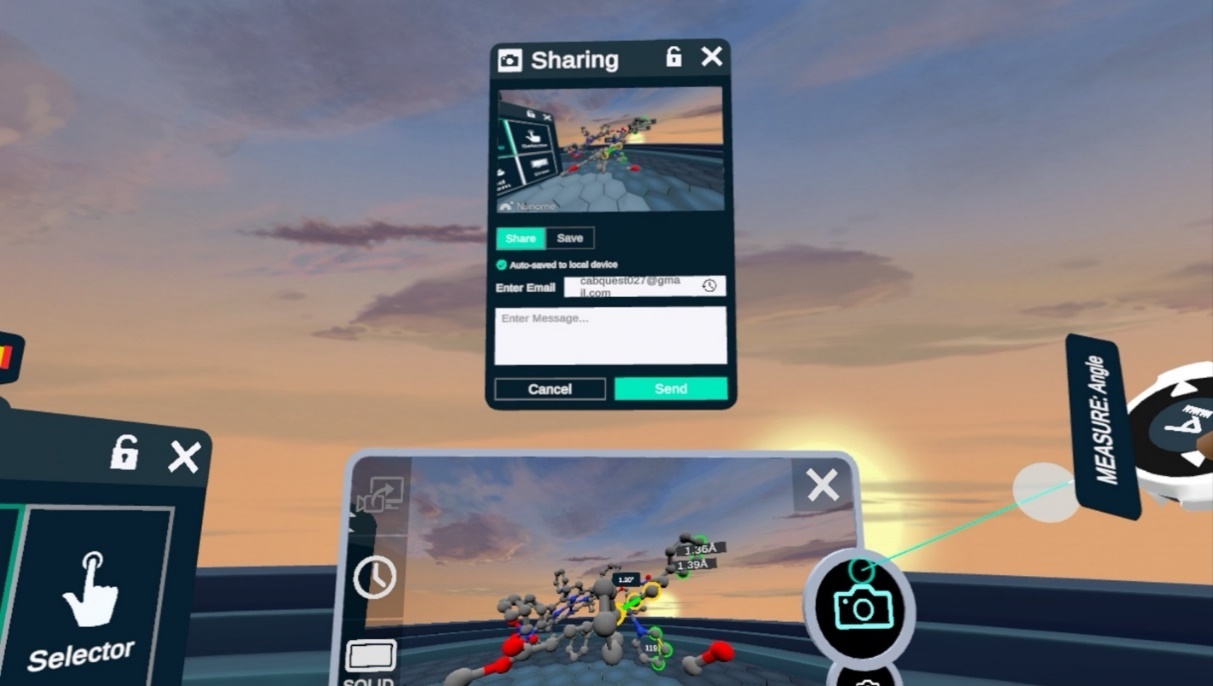
**Figure SF31** How to email screenshots of the molecule in the 3D platform using the trigger button.

**S5. Survey Introduction for Undergraduate Study: “From Crystal Structure to 3D Printing to Virtual reality (VR) in the Class.**

Participants of this study were undergraduate students (over 18 years of age) at Harvard College. All students took the survey through the Qualtrics platform (https://harvard.az1.qualtrics.com/jfe/form/SV_6l0FZxDiniGkcV8) after the conclusion of the module.

All students were given printed versions of the procedure as presented in this publication and were asked to complete the module. Each student was equipped with:

- A laptop with internet connection (for CCDC access) and Mercury software (for CIF viewing, file conversion, and export).
- Access to the Bambu 3-D printer.
- Nail polishes (Sally Hansen brand) including all colours necessary for the metals (magnesium – purple; platinum – dark blue; palladium – yellow; iron – orange) and nonmetals (carbon – gray; nitrogen – blue; oxygen – red; fluorine – light green; chlorine – neon green) included in this module.
- A Meta Quest 3 virtual reality headset with Nanome software.

Students were given independent structure assignments but were allowed to work collaboratively with their peers to problem-solve and overcome challenges. Teaching staff provided support only, when necessary, as requested by the students.

Students were given Crystal Structure Identifiers (CSD Refcodes) including JUGYIX (CSD Refcode) – Fe porphyrin (Brown *et al.*, 2023), JUGYET (CSD Refcode)–Mg porphyrin, GOPZEU (CSD Refcode) – Pt porphyrin (Dash *et al.*, 2024), (Brown *et al.*, 2023), and CEPHEO (CSD Refcode)–Pd porphyrin (Chrisp, *et al.*, 2022) (Table S2).

**Table S2** The list of CCDC CSD Reference codes.

| **Crystal Structure Identifiers (CSD Refcodes)** | **Publication** | **Structure** |
| --- | --- | --- |
| GOPZEU | Dash et al., 2024 | 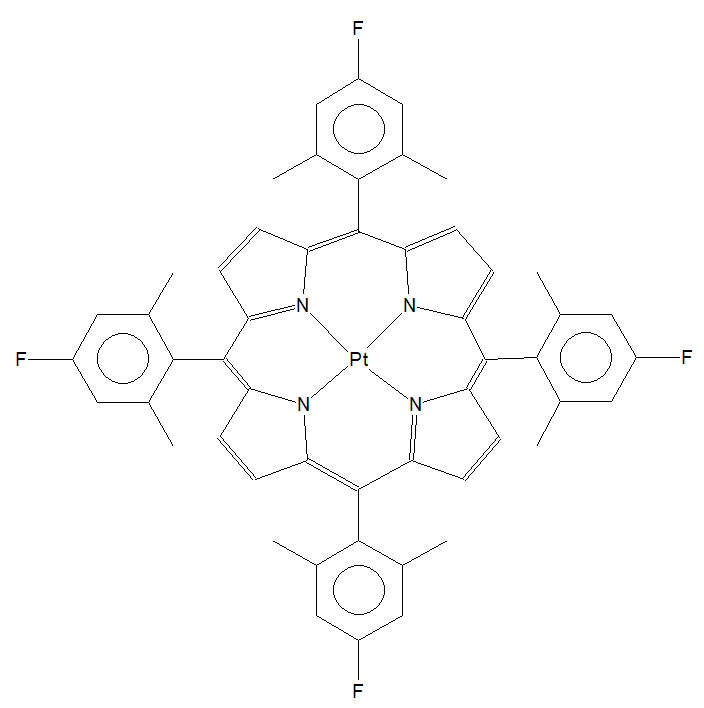 |
| JUGYET | Brown et al., 2023 | 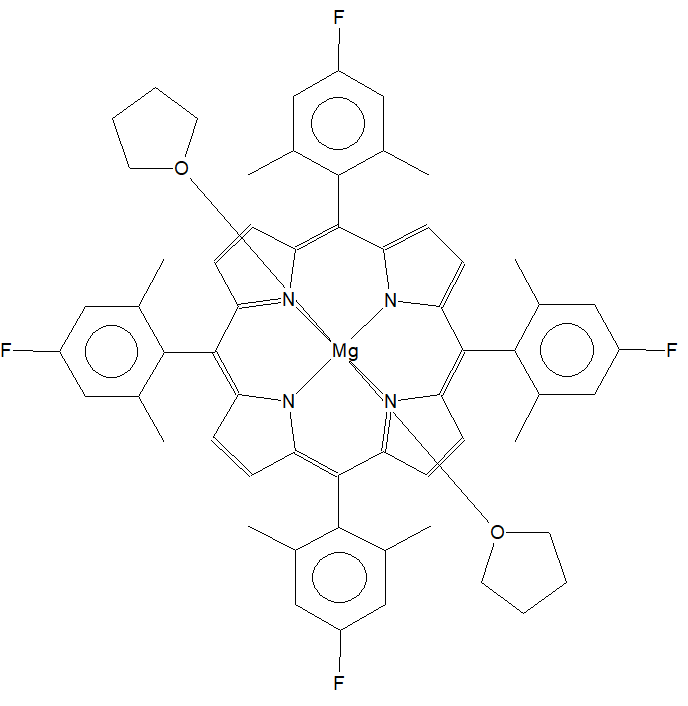 |
| JUGYIX | Brown et al., 2023 | 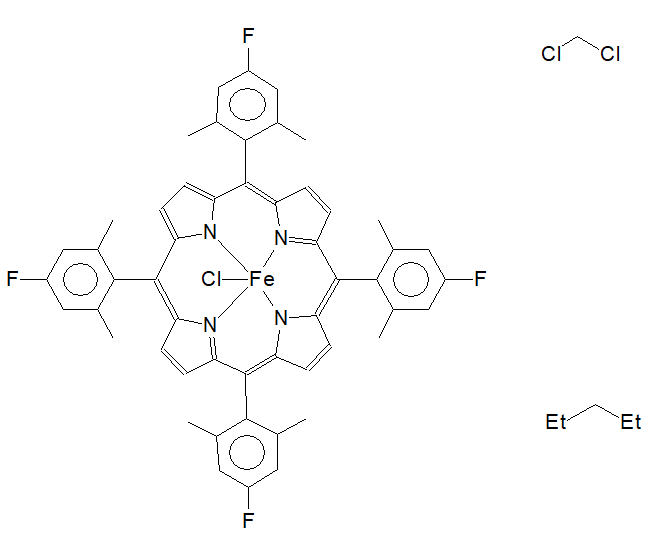 |
| CEPHEO | Crisp et al., 2022 | 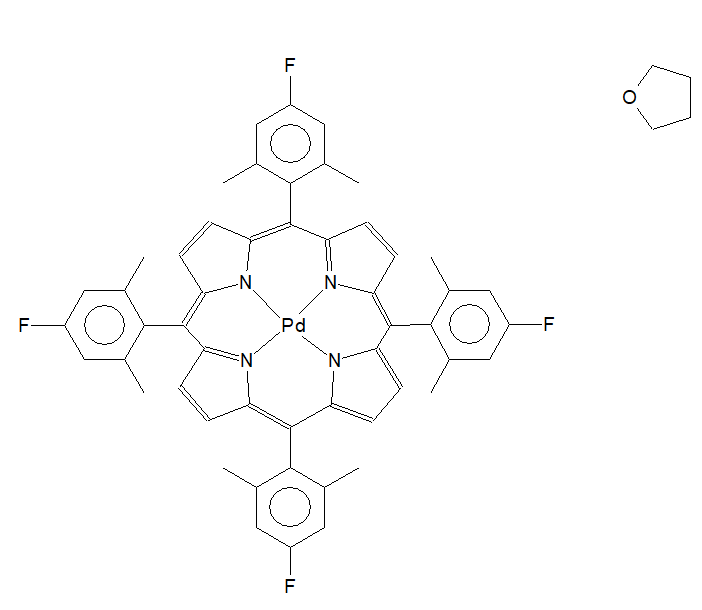 |
| WEKKEG | Chou et al., 2022 | 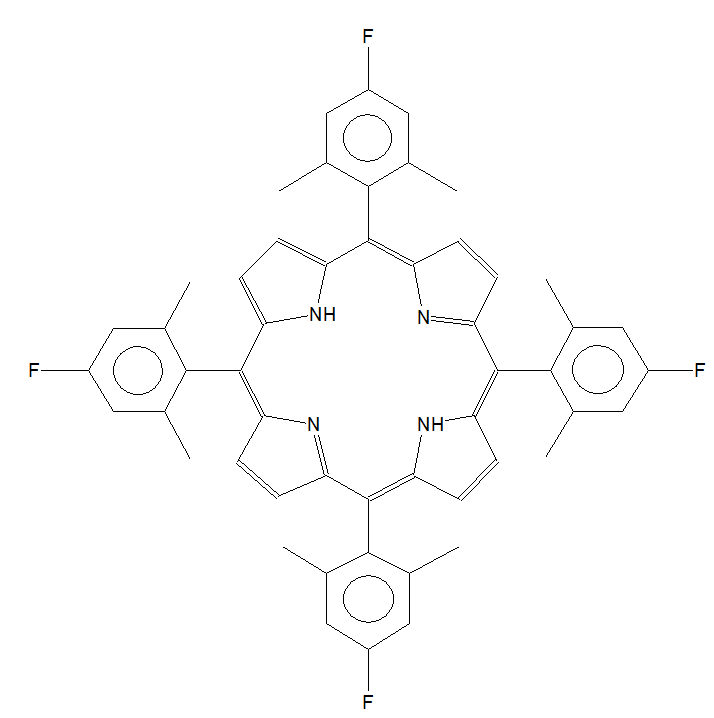 |

Recodes of the porphyrins studies in this study.

**S6. Survey Questions (with results; %, fraction of total) for Undergraduate Study: “From Crystal Structure to 3-D Printing to Virtual Reality in the Classroom”**

**S6.1 Demographics**

1. **Are you over 18 years of age?**

Yes (100%, 15/15) No (0%, 0/15)

1. **What year of study have you *completed* at Harvard College?**

I’ve completed my first (freshman) year. (60%, 9/15)

I’ve completed my second (sophomore) year. (20%, 3/15)

I’ve completed my third (junior) year. (20%, 3/15)

I’ve completed my fourth (senior) year. (0%, 0/15)

1. **How would you categorize your concentration?**

Chemistry concentration. (67%, 10/15)

A STEM concentration (but not chemistry). (33%, 5/15)

A non-STEM concentration. (0%, 0/15)

1. **What is your concentration?**

Chemistry (53%, 8/15)

Molecular and Cellular Biology (13%, 2/15)

Chemical and Physical Biology (7%, 1/15)

Chemistry and Physics (7%, 1/15)

Neuroscience (7%, 1/15)

Integrative Biology (7%, 1/15)

Bioengineering (7%, 1/15)

1. **What type of computer system are you using?**

Windows (40%, 6/15) Mac (47%, 7/15) Other (13%, 2/15)

1. **Please rank the amount of experience, previous to this module, you have with the following topics on a scale of 1 to 5 (1 – no experience, 2 – I’ve heard of it, but have no direct experience, 3 – some experience, 4 – used this before, but am not independently proficient, 5 – quite experienced).**
   1. **Using the Cambridge Structural Database to retrieve crystal structures.**

1 (67%, 10/15) 2 (13%, 2/15) 3 (13%, 2/15) 4 (7%, 1/15) 5 (0%, 0/15)

- 1. **Viewing and manipulating crystal structures.**

1 (60%, 9/15) 2 (7%, 1/15) 3 (13%, 2/15) 4 (20%, 3/15) 5 (0%, 0/15)

- 1. **3D Printing**

1 (47%, 7/15) 2 (27%, 4/15) 3 (27%, 4/15) 4 (0%, 0/15) 5 (0%, 0/15)

- 1. **Virtual Reality**

1 (20%, 3/15) 2 (47%, 7/15) 3 (27%, 4/15) 4 (7%, 1/15) 5 (0%, 0/15)

**S6.2. Usability**

1. **Finding the crystal structure from CCDC was…**

Difficult (0%, 0/15) Somewhat difficult (33%, 5/15) Not difficult (67%, 10/15)

1. **Converting the file to a 3D-printable format was…**

Difficult (13%, 2/15) Somewhat difficult (33%, 5/15) Not difficult (53%, 8/15)

1. **Printing a 3D structure of the molecule was…**

Difficult (13%, 2/15) Somewhat difficult (27%, 4/15) Not difficult (60%, 9/15)

1. **Identifying the identity of atoms on the printed structures was…**

Difficult (7%, 1/15) Somewhat difficult (13%, 2/15) Not difficult (80%, 12/15)

1. **Visualizing the 3D structure of the molecule in the VR platform was…**

Difficult (27%, 4/15) Somewhat difficult (27%, 4/15) Not difficult (47%, 7/15)

1. **Identifying the bond lengths on the molecule using VR set was…**

Difficult (13%, 2/15) Somewhat difficult (20%, 3/15) Not difficult (67%, 10/15)

1. **Identifying the angles of the bonds on the molecule using VR set was…**

Difficult (7%, 1/15) Somewhat difficult (27%, 4/15) Not difficult (67%, 10/15)

1. **Please describe what you found most challenging in this module.**

“I found orienting myself in VR most difficult.”

“Identifying the atoms.”

“Navigating the VR user interfaces.”

“Using the controller without being able to use it.”

“Giving the presenting rights to the right person.”

“Getting used to VR.”

“Clicking on atoms in the model.”

“Learning how to use the VR. Once you got the hang of it, then it became efficient.”

“I think there was little difficulty in getting acquainted with the VR at first but it became quite easy with some practice.”

“Getting the internet to let us load the molecules.”

“Our molecule did not load.”

“I found VR part to be hardest.”

“Figuring out how to load the molecules on VR.”

“The Mercury structure to the 3D model was probably most challenging.”

“Probably figuring out how to use the controls and the joystick. But I got the hang of it.”

1. **Please describe what you found the easiest in this module.**

“Downloading the structure file was the easiest.”

“Identifying the angles and distance.”

“Visualizing/understanding how the molecules composition”

“Everything else was easy, especially measuring!”

“Moving”

“Transferring presenter.”

“How easy the molecule was to visualize in a 3D structure.”

“I thought using the tools was quite easy.”

“Playing around in the virtual reality sandbox was so fun!”

“Visualizing and moving the molecule.”

“The first part was easier, prior to using the VR headset.”

“I thought once we got the molecule loaded, figuring out the bond lengths and angles was pretty simple.”

“I found the VR pretty easy and fun!”

“Finding the bond lengths were the easiest.”

1. **Did you experience any dizziness or disorientation when using the VR headset?**

Yes (13%, 2/15) No (87%, 13/15)

**S6.3. Learning**

1. **Printing the 3D structure of the molecule helped me understand the properties (e.g. geometry, bonding, angles, coordination environment, packing) of the molecule.**

Yes (100%, 15/15) No (0%, 0/15)

1. **Using the VR set helped me understand the properties (geometry, bonding, angles, coordination environment, packing) of the molecule.**

Yes (93%, 14/15) No (7%, 1/15)

1. **Did you find this exercise helpful in learning chemistry?**

Yes (93%, 14/15) No (7%, 1/15)

1. **How would you rank this exercise in your chemistry learning/understanding, on a scale of 1 to 5 (1 – this significantly hindered my understanding of chemistry, 2 – this hindered my understanding more than it helped my understanding of chemistry, 3 – this neither hindered nor helped my understanding of chemistry, 4 – this helped me understand chemistry concepts somewhat, 5 – this really helped me understand certain chemistry concepts)?**

1 (0%, 0/15) 2 (0%, 0/15) 3 (27%, 4/15) 4 (60%, 9/15) 5 (13%, 2/15)

1. **What was the CSD Reference Code of the structure you were assigned?**

JUGYIX (33%, 5/15)

GOPZEU (27%, 4/15)

JUGYET (20%, 3/15)

CEPHEO (20%, 3/15)

1. **Given your structure, please use your 3D model and VR experience to define the coordination geometry around the metal.**

I have no metal Square Planar Square Pyramidal Octahedral

Correct responses (based on answer to question Learning 5): (87%, 13/15)

Incorrect responses (based on answer to question Learning 5): (13%, 2/15)

1. **Given your structure, please use your 3D model and VR experience to select all non-metal elements present in your molecule. Select all non-metals that you identify.**

Hydrogen(H) Carbon(C) Nitrogen(N) Oxygen(O) Fluorine(F) Chlorine(Cl)

For a “correct” response, respondents had to select *all* elements present, and no additional elements. Partially correct answers were counted as “incorrect.”

Correct responses (based on answer to question Learning 5): (60%, 9/15)

Incorrect responses (based on answer to question Learning 5): (40%, 6/15)

1. **Given your structure, please use your 3D model and VR experience to select all metal elements present in your molecule. Select all metals that you identify.**

No metal Iron(Fe) Magnesium(Mg) Palladium(Pd) Platinum(Pt)

Correct responses (based on answer to question Learning 5): (93%, 14/15)

Incorrect responses (based on answer to question Learning 5): (7%, 1/15)

**S6.4. Engagement**

1. **Which method of looking at the crystal structure did you find most engaging?**

On my computer screen within Mercury (0%, 0/15)

As a 3D printed model (20%, 3/15)

In the virtual reality headset (80%, 12/15)

1. **Which method of looking at the crystal structure did you find most intuitive (natural to understand)?**

On my computer screen within Mercury (7%, 1/15)

As a 3D printed model (67%, 10/15)

In the virtual reality headset (27%, 4/15)

1. **If you were asked to identify how molecules pack within a crystal structure, which would be your preferred method to visualize this?**

On my computer screen within Mercury (27%, 4/15)

As a 3D printed model (40%, 6/15)

In the virtual reality headset (33%, 5/15)

1. **If you were asked to identify bond angles within a molecule (e.g. which angles are close to 90°) , which would be your preferred method to visualize this?**

On my computer screen within Mercury (27%, 4/15)

As a 3D printed model (7%, 1/15)

In the virtual reality headset (67%, 10/15)

1. **If you were asked to identify bond lengths within a molecule, which would be your preferred method to visualize this?**

On my computer screen within Mercury (27%, 4/15)

As a 3D printed model (7%, 1/15)

In the virtual reality headset (67%, 10/15)

1. **If you were asked to identify coordination geometry around a metal within a molecule (e.g. square planar, square pyramidal, octahedral), which would be your preferred method to visualize this?**

On my computer screen within Mercury (40%, 6/15)

As a 3D printed model (40%, 6/15)

In the virtual reality headset (20%, 3/15)

**S7. Survey Results for Undergraduate Study: “From Crystal Structure to 3-D Printing to Virtual Reality in the Classroom”**

**
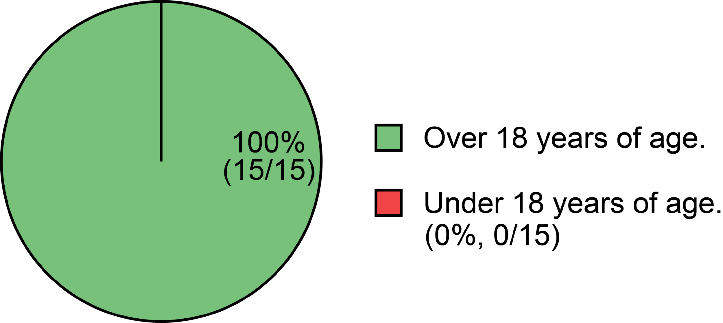
S7.1. Demographics Figures**

**Figure SF32** Pie chart showing results of Demographics Question 1 – “*Are you over 18 years of age*?”


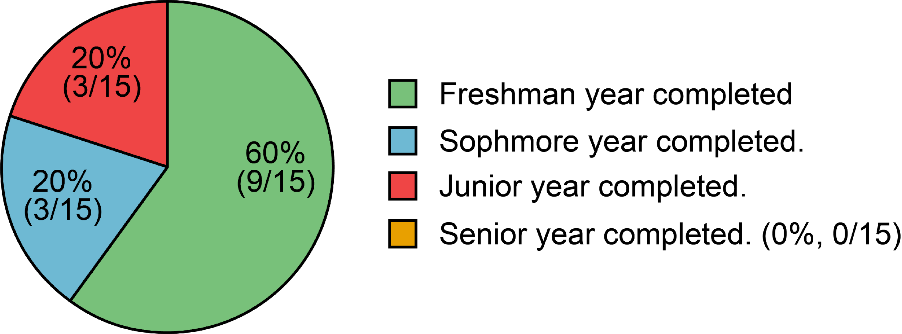


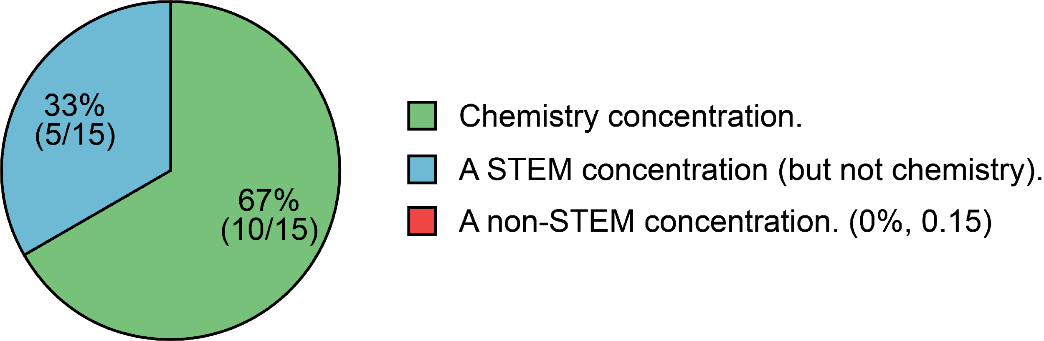
**Figure SF33** Pie chart showing results of Demographics Question 2 – “*What year of study have you completed at Harvard College*?.”

**Figure SF34** Pie chart showing results of Demographics Question 3 – *“How would you categorize your concentration?”*


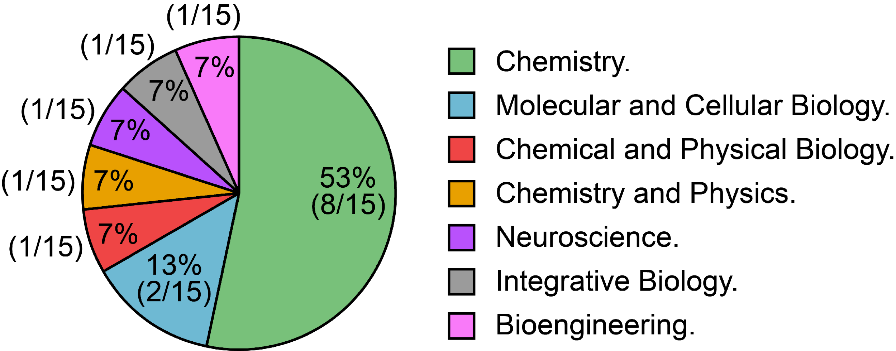


**Figure SF35** Pie chart showing results of Demographics Question 4 – *“What is your concentration?”*


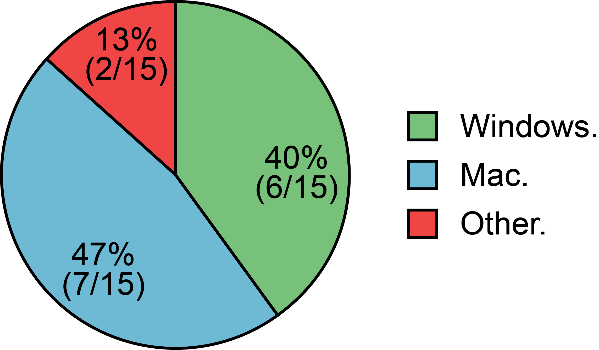


**Figure SF36** Pie chart showing results of Demographics Question 5 – *“What type of computer system are you using?”*


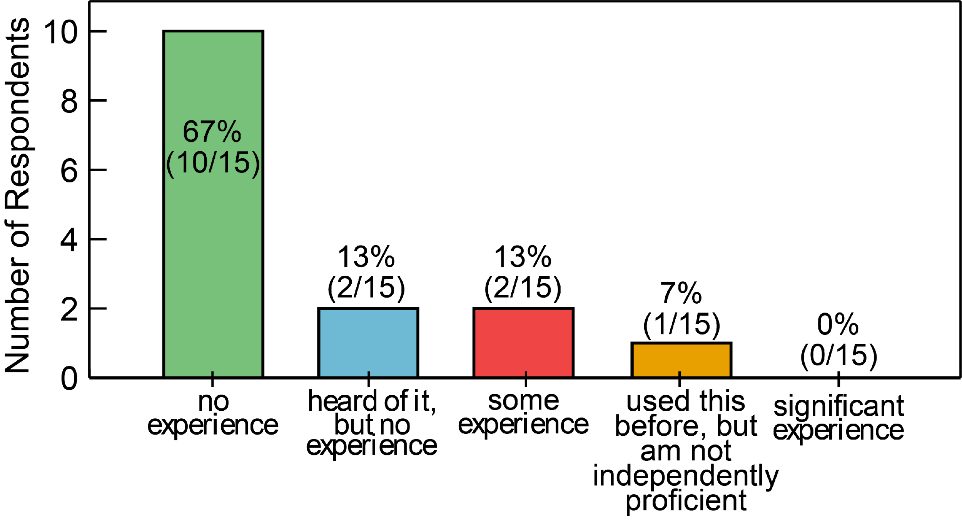


**Figure SF37** Bar graph showing results of Demographics Question 6a – “*Please rank the amount of experience, previous to this module, you have with the following topic on a scale of 1 to 5: Using the Cambridge Structural Database to retrieve crystal structures.”*


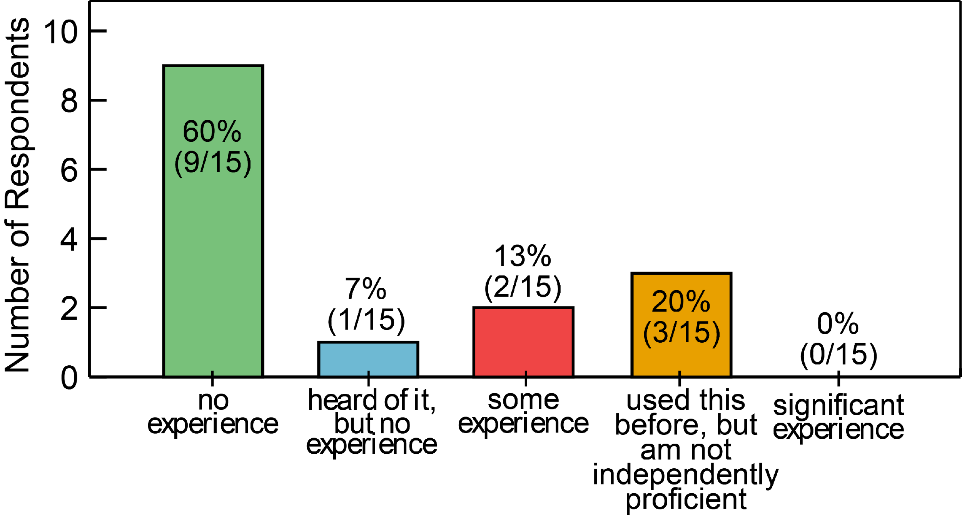


**Figure SF38** Bar graph showing results of Demographics Question 6b – *“Please rank the amount of experience, previous to this module, you have with the following topic on a scale of 1 to 5: Viewing and manipulating crystal structures.”*


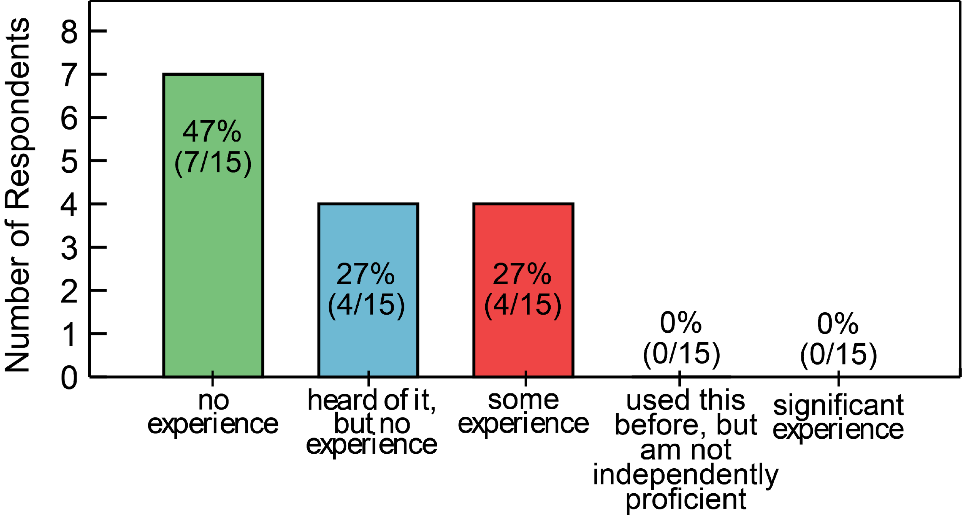


**Figure SF39** Bar graph showing results of Demographics Question 6c – *“Please rank the amount of experience, previous to this module, you have with the following topic on a scale of 1 to 5: 3-D printing.”*

**
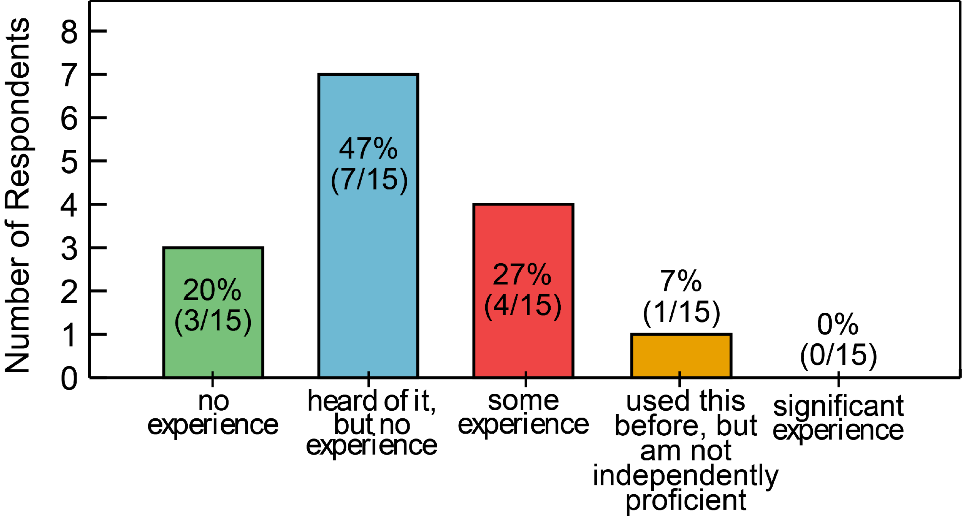
**

**Figure SF40** Bar graph showing results of Demographics Question 6d – *“Please rank the amount of experience, previous to this module, you have with the following topic on a scale of 1 to 5: Virtual reality.”*

**S7.2.**
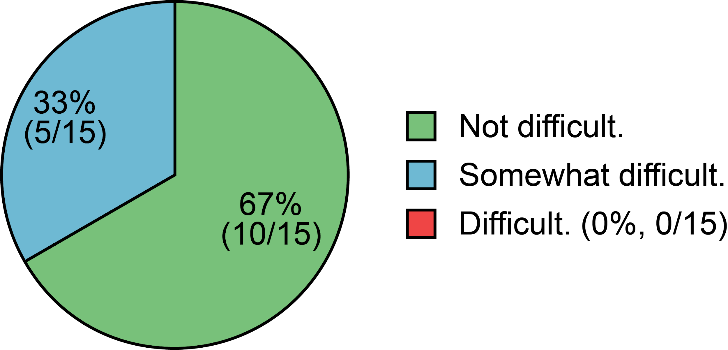
**Usability Figures**

**Figure SF41** Pie chart showing results of Usability Prompt 1 – “*Finding the crystal structure from CCDC was…”*

**
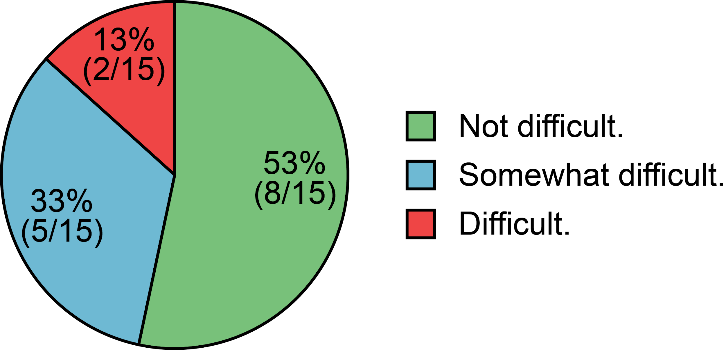
**

**Figure SF42** Pie chart showing results of Usability Prompt 2 – *“Converting the file to a 3D-printable format was…”*


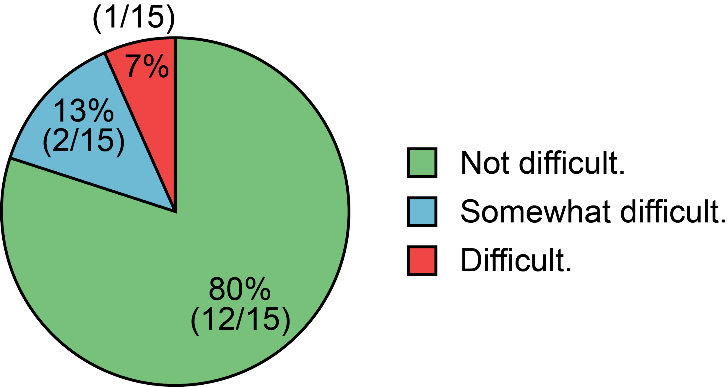


**
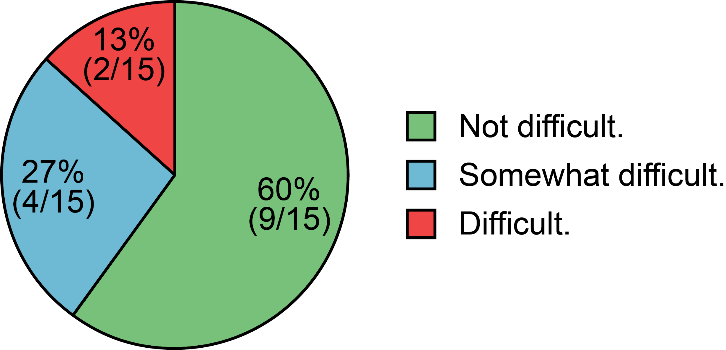
Figure SF43** Pie chart showing results of Usability Prompt 3 – *“Printing a 3D structure of the molecule was…”*

**Figure SF44** Pie chart showing results of Usability Prompt 4 – *“Identifying the identity of atoms on the printed structures was…”.*


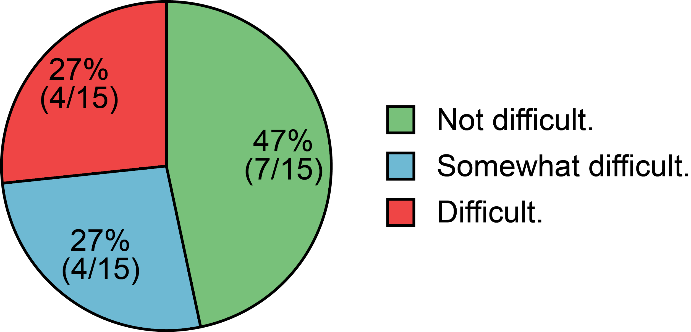


**Figure SF45** Pie chart showing results of Usability Prompt 5 – *“Visualizing the 3D structure of the molecule in the VR platform was…”*.


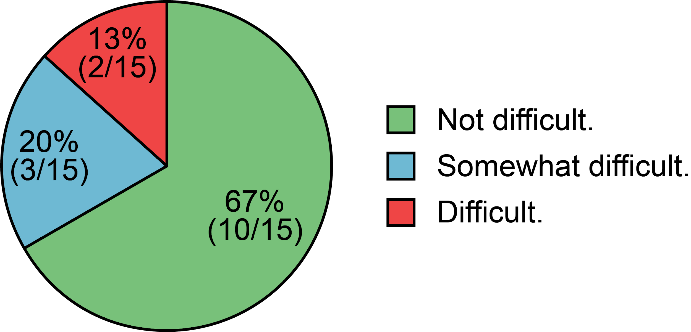


**Figure SF46** Pie chart showing results of Usability Prompt 6 – *“Identifying the bond lengths on the molecule using VR set was…”*


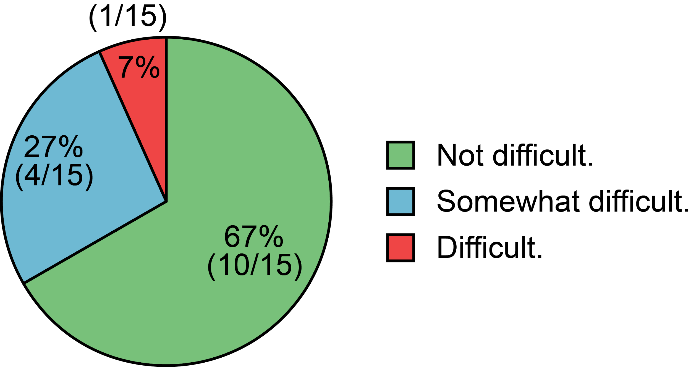


**Figure SF47** Pie chart showing results of Usability Prompt 7 – *“Identifying the angles of the bonds on the molecule using VR set was…”*


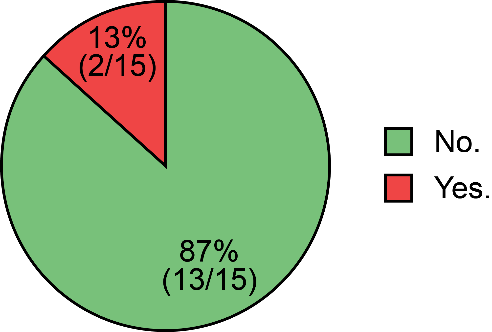


**Figure SF48** Pie chart showing results of Usability Question 10 – “*Did you experience any dizziness or disorientation when using the VR headset?”*

**S7.3. Learning Figures**

**
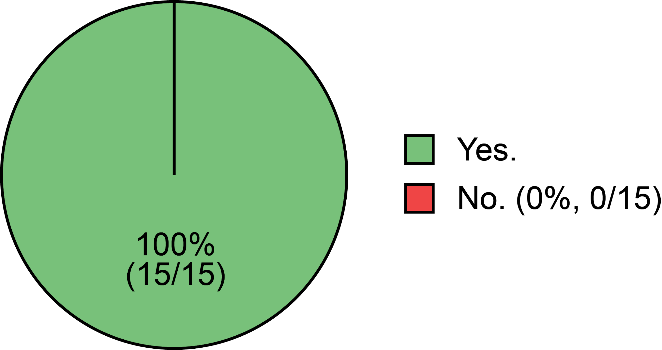
**


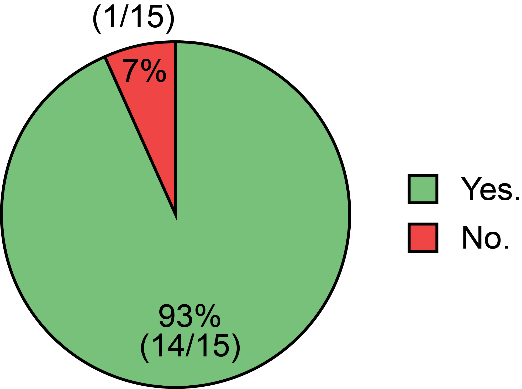
**Figure SF49** Pie chart showing results of Learning Prompt 1 – *“Printing the 3D structure of the molecule helped me understand the properties (e.g. geometry, bonding, angles, coordination environment, packing) of the molecule.”*

**Figure SF50** Pie chart showing results of Learning Prompt 2 – *“Using the VR set helped me understand the properties (geometry, bonding, angles, coordination environment, packing) of the molecule.”*


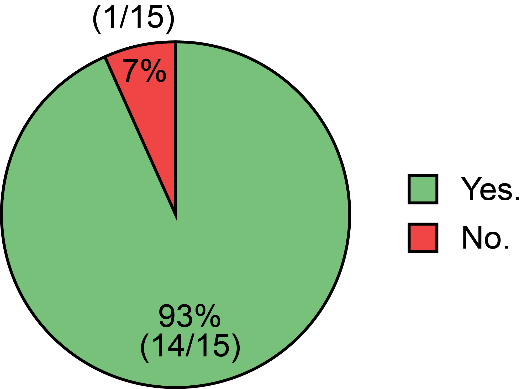


**Figure SF51** Pie chart showing results of Learning Question 3 – *“Did you find this exercise helpful in learning chemistry?”*


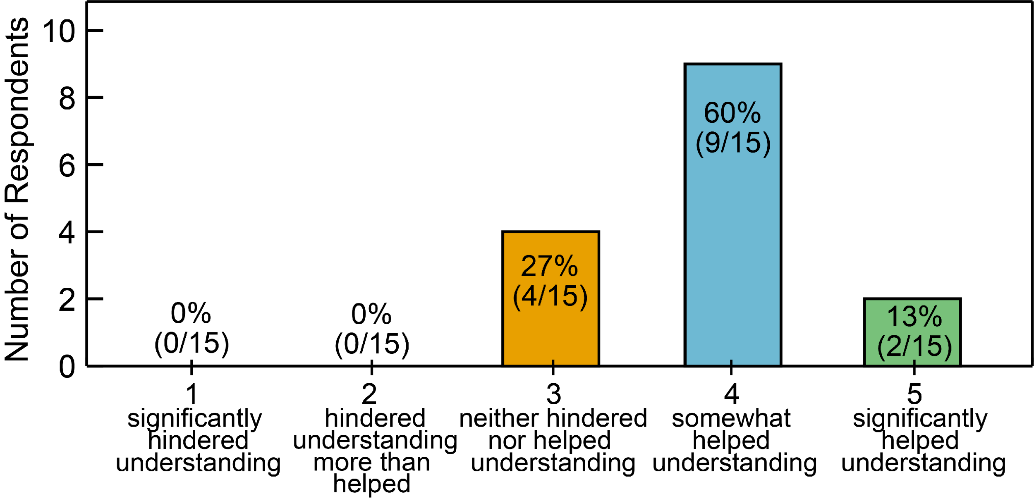


**Figure SF52** Bar graph showing results of Learning Question 4 – *“How would you rank this exercise in your chemistry learning/understanding, on a scale of 1 to 5?”*

**
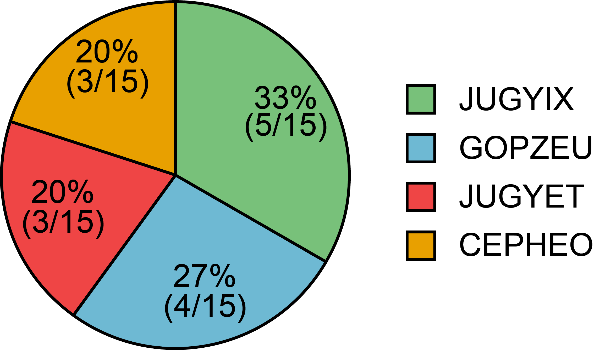
**

**Figure SF53** Pie chart showing breakdown of CSD reference code assignments, indicated by Learning Question 5 – *“What was the CSD Reference Code of the structure you were assigned?”*


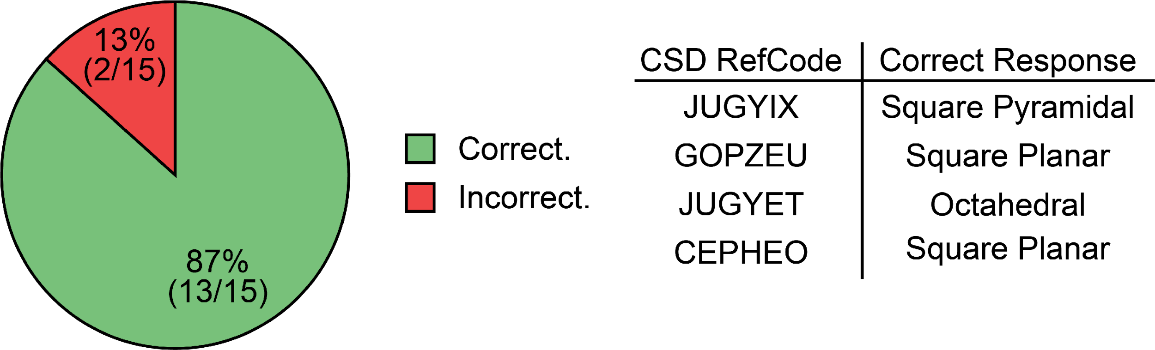


**Figure SF54** Pie chart showing breakdown of correct/incorrect coordination geometry assignments, indicated by the results of Learning Question 6 – *“Given your structure, please use your 3-D model and VR experience to define the coordination geometry around the metal”* – paired with the information from Learning Question 5 about CSD Reference Code assignments.

**
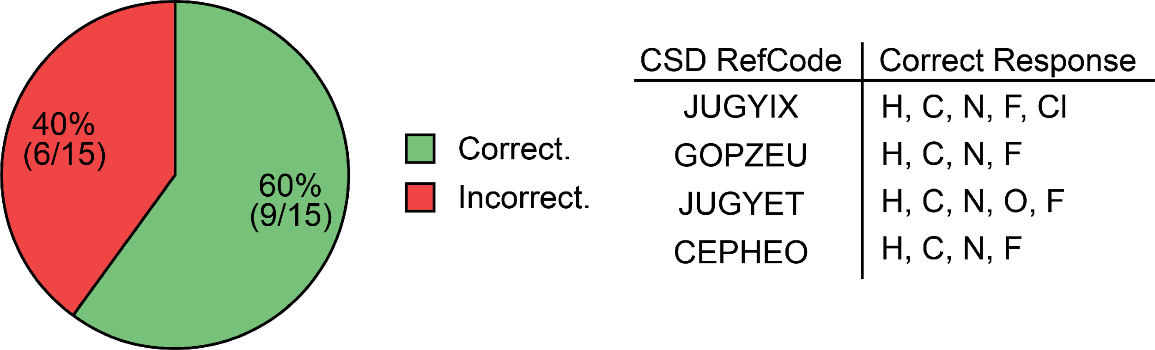
**

**Figure SF55** Pie chart showing breakdown of correct/incorrect non-metal element assignments, indicated by the results of Learning Question 7 – “*Given your structure, please use your 3D model and VR experience to select all non-metal elements present in your molecule”* – paired with the information from Learning Question 5 about CSD Reference Code assignments. For a “correct” response, respondents had to select *all* elements present, and no additional elements. Partially correct answers were counted as “incorrect.”


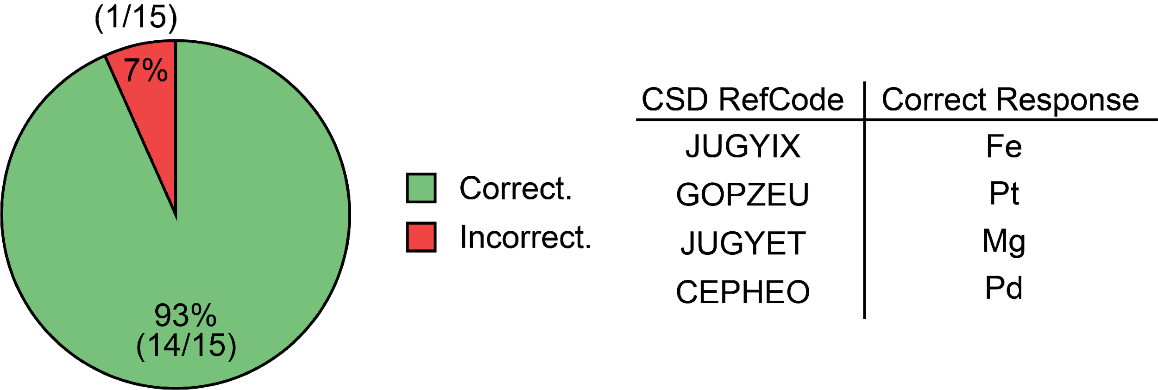


**Figure SF56** Pie chart showing breakdown of correct/incorrect metal element assignments, indicated by the results of Learning Question 8 – *“Given your structure, please use your 3D model and VR experience to select all metal elements present in your molecule”* – paired with the information from Learning Question 5 about CSD Reference Code assignments.

**S7.4. Engagement Figures**

**
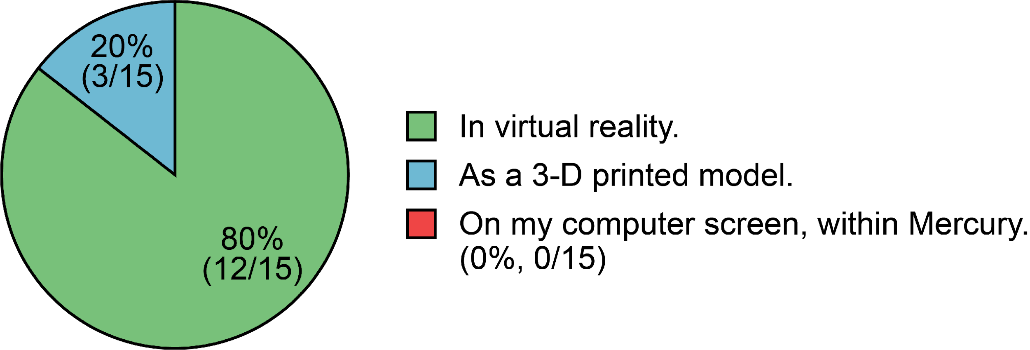
**


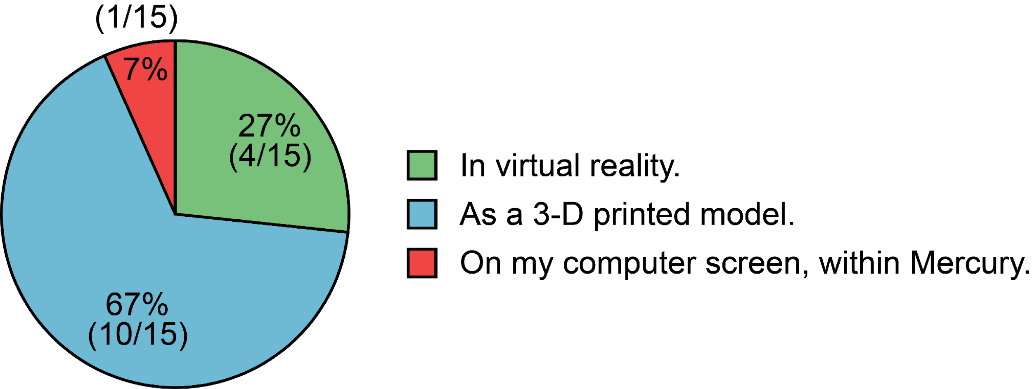
**Figure FS57** Pie chart showing results of Engagement Question 1 – *“Which method of looking at the crystal structure did you find most engaging?”*

**Figure SF58** Pie chart showing results of Engagement Question 2 – *“Which method of looking at the crystal structure did you find most intuitive (natural to understand)?”*


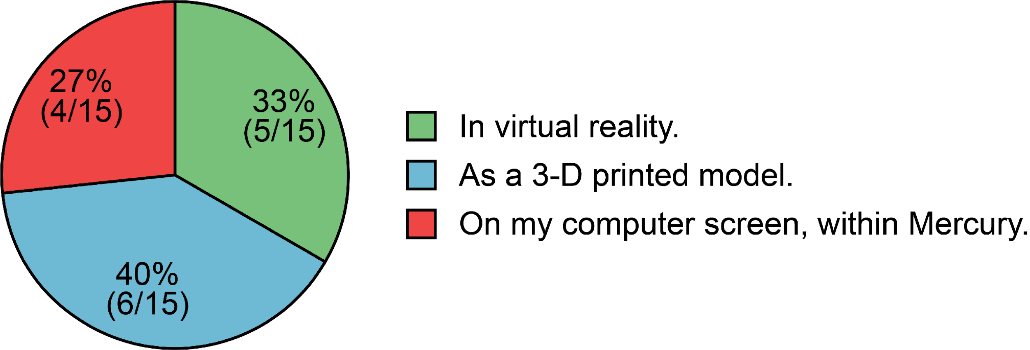


**Figure SF59** Pie chart showing results of Engagement Question 3 – *“If you were asked to identify how molecules pack within a crystal structure, which would be your preferred method to visualize this?*


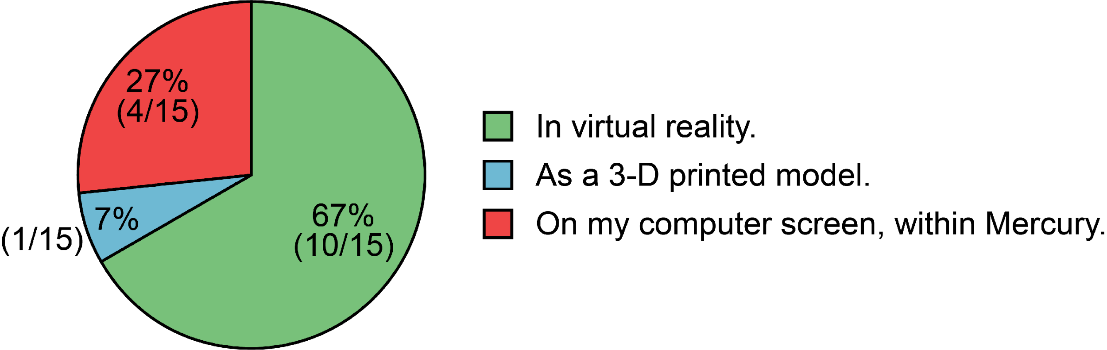


**Figure SF60** Pie chart showing results of Engagement Question 4 – *“If you were asked to identify bond angles within a molecule (e.g. which angles are close to 90°), which would be your preferred method to visualize this?”*


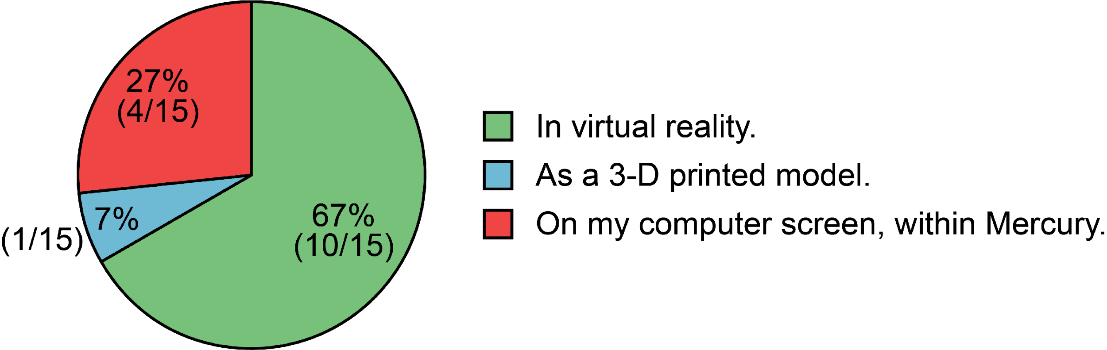


**Figure SF61** Pie chart showing results of Engagement Question 5 – *“If you were asked to identify bond lengths within a molecule, which would be your preferred method to visualize this.?*”


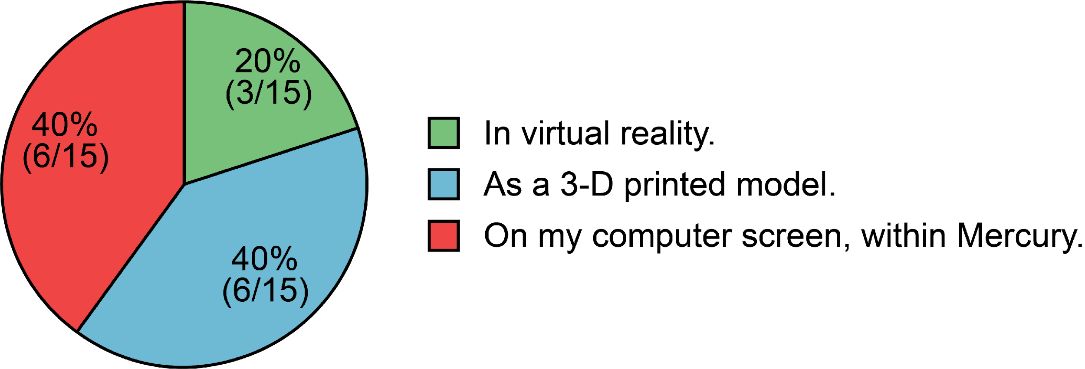


**Figure SF62** Pie chart showing results of Engagement Question 6 – “*If you were asked to identify coordination geometry around a metal within a molecule (e.g. square planar, square pyramidal, octahedral), which would be your preferred method to visualize this?”*

1. Appendix A. The survey questions


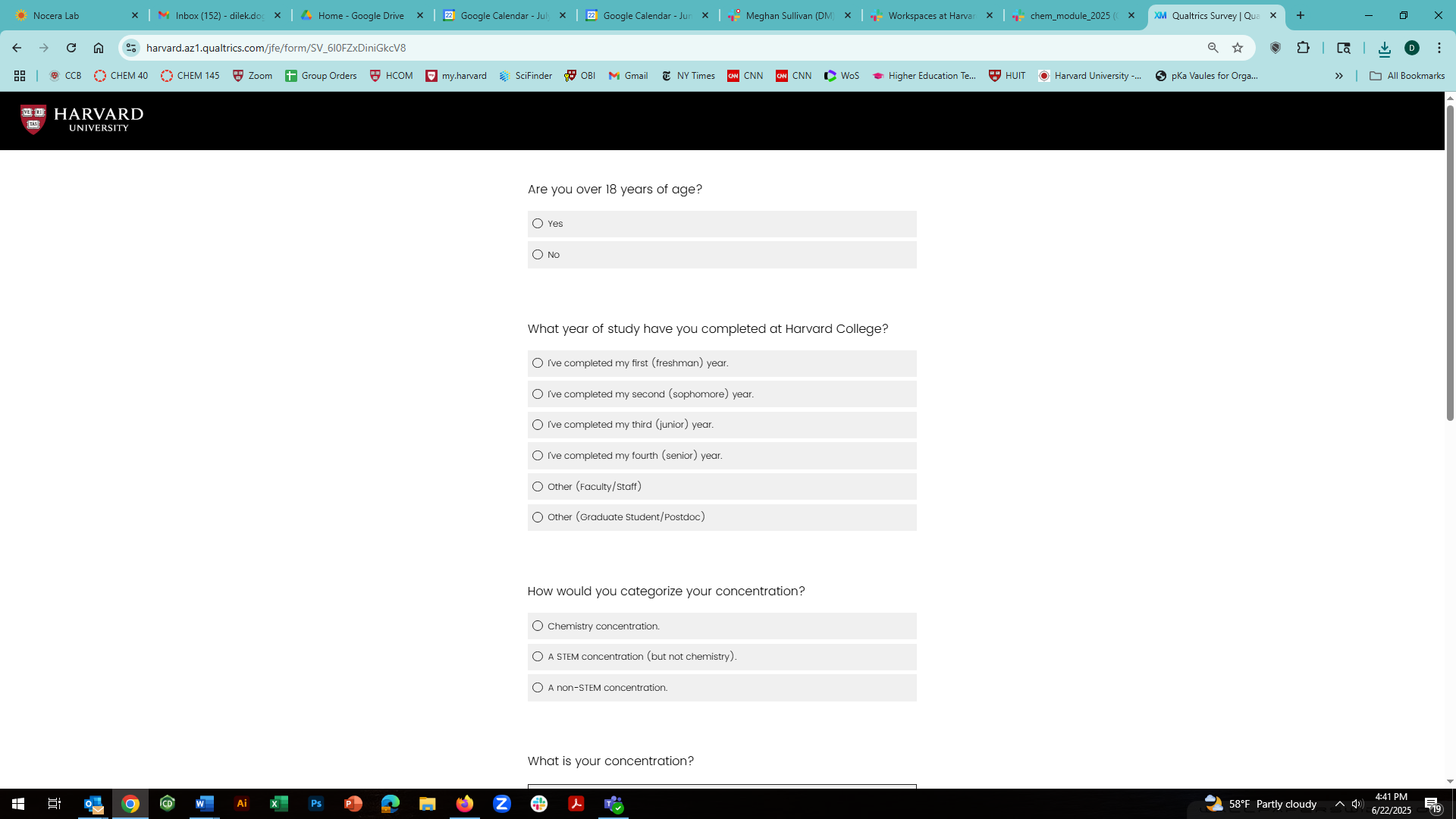


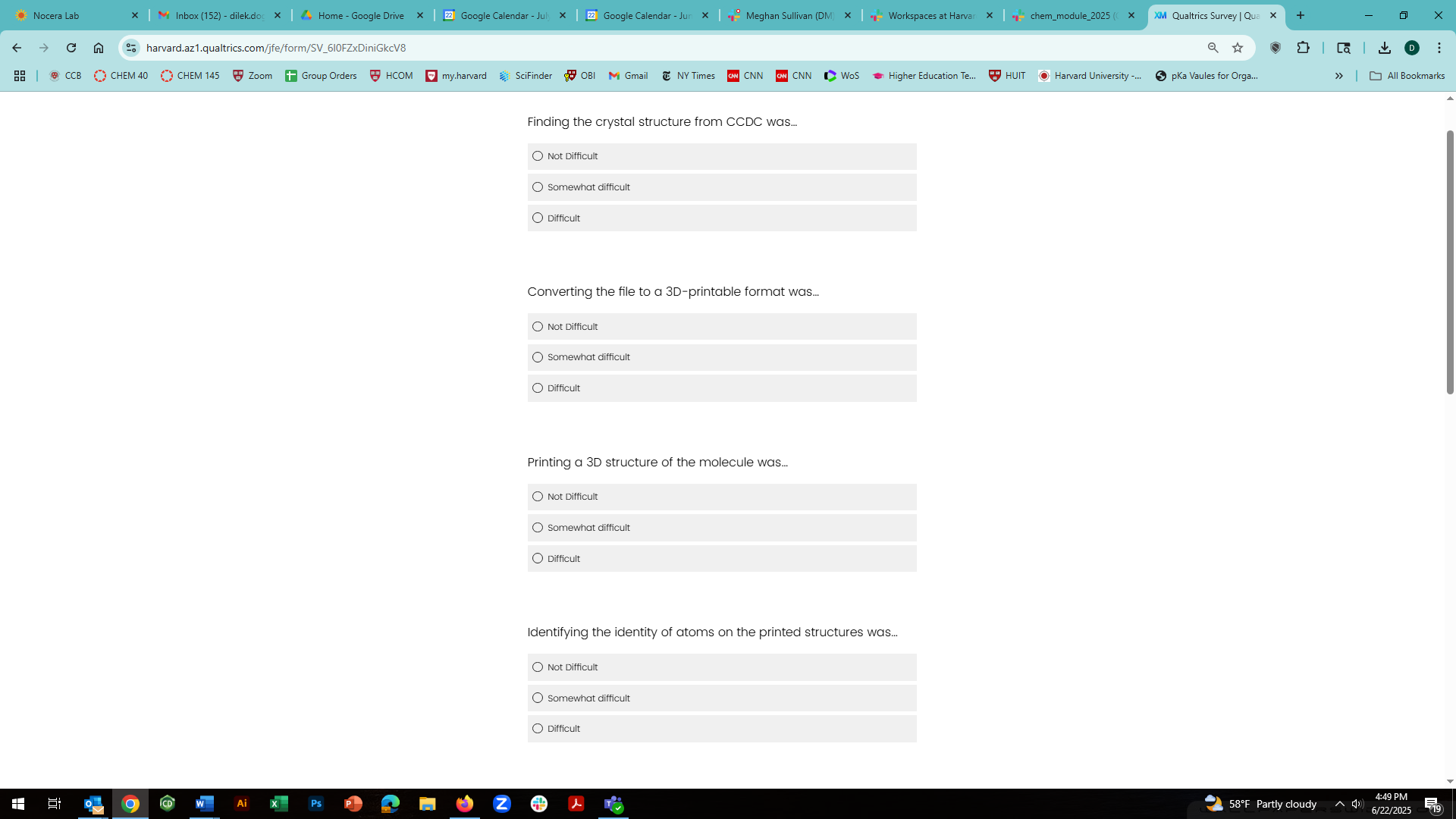


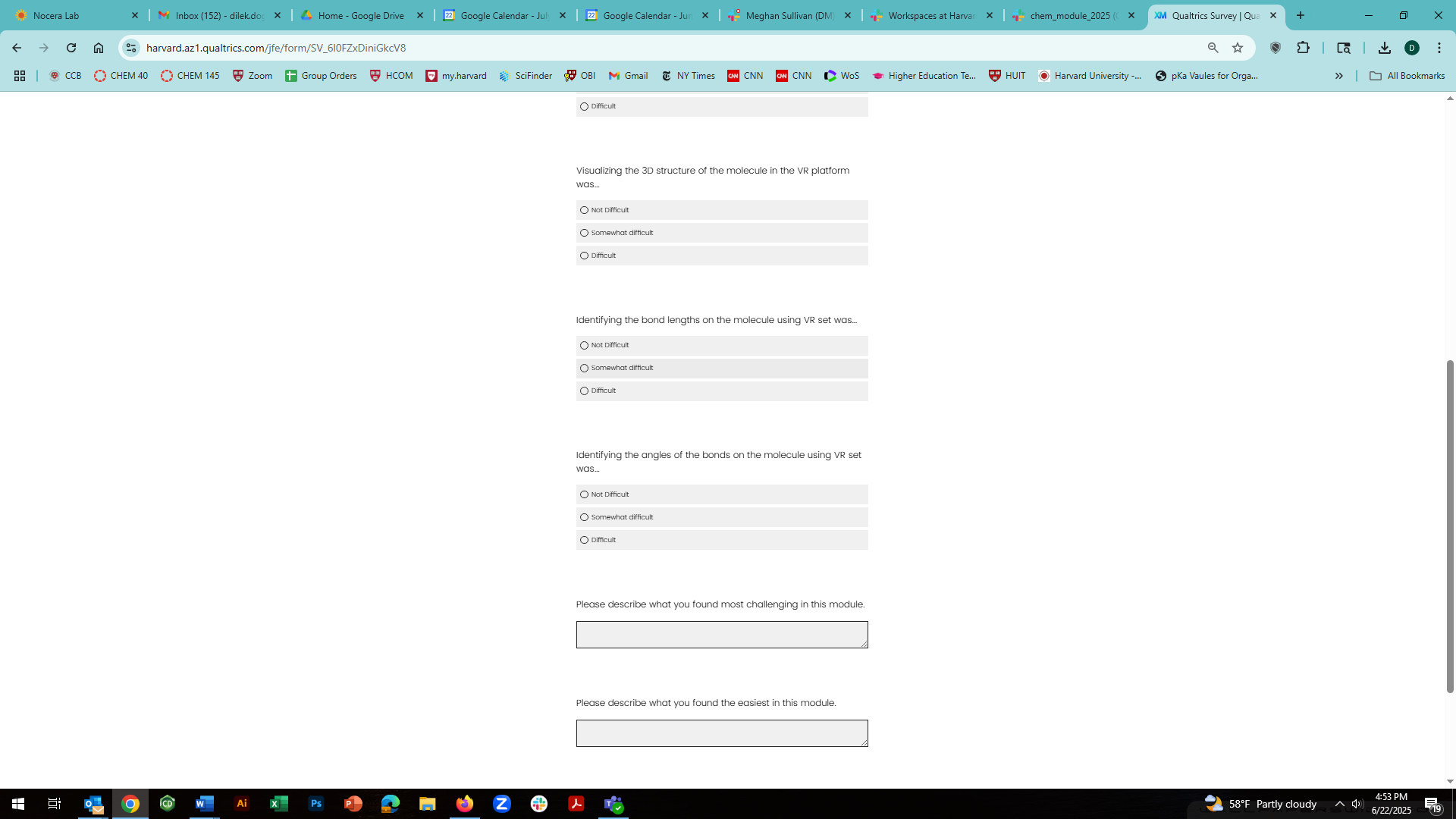


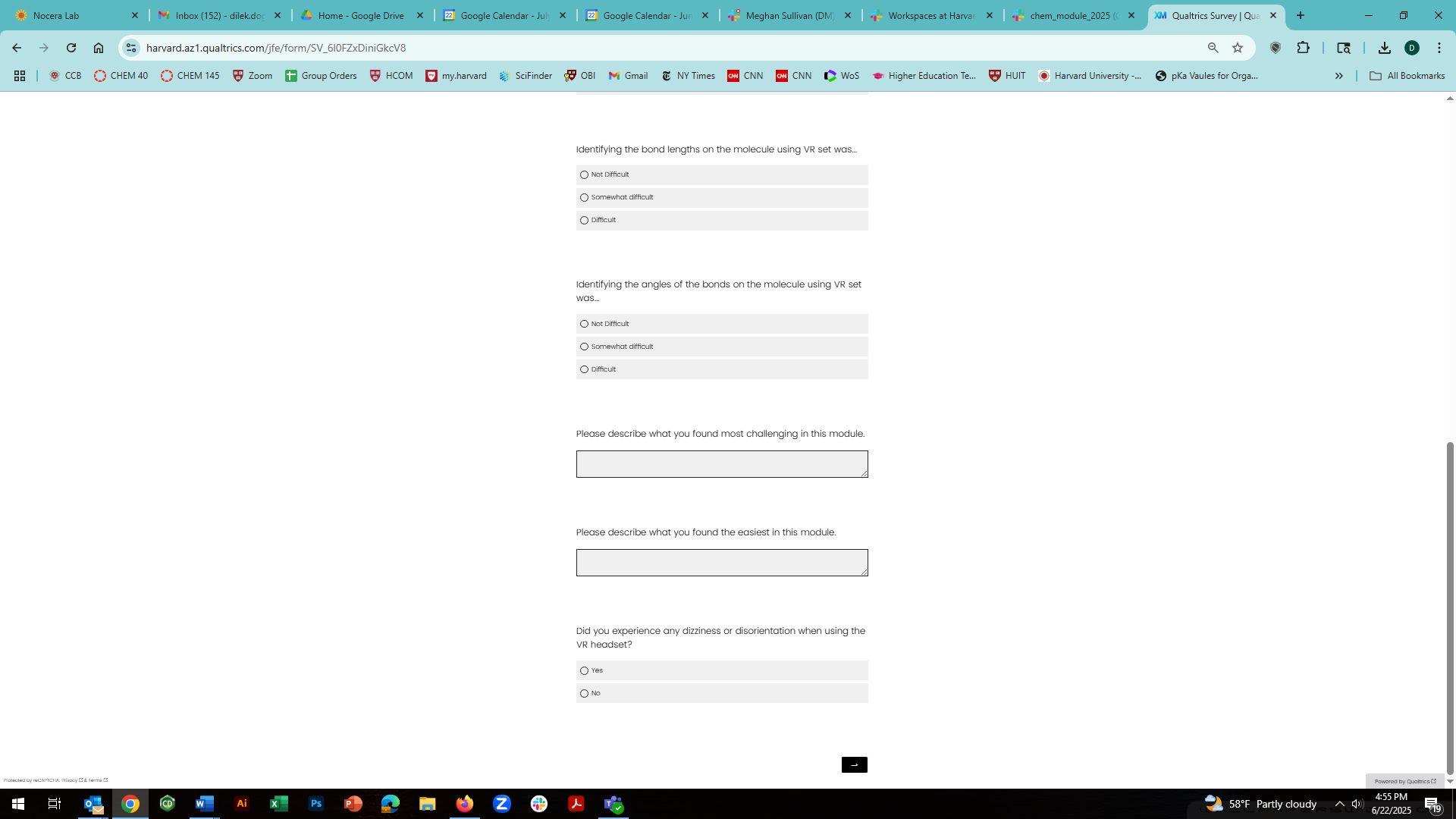


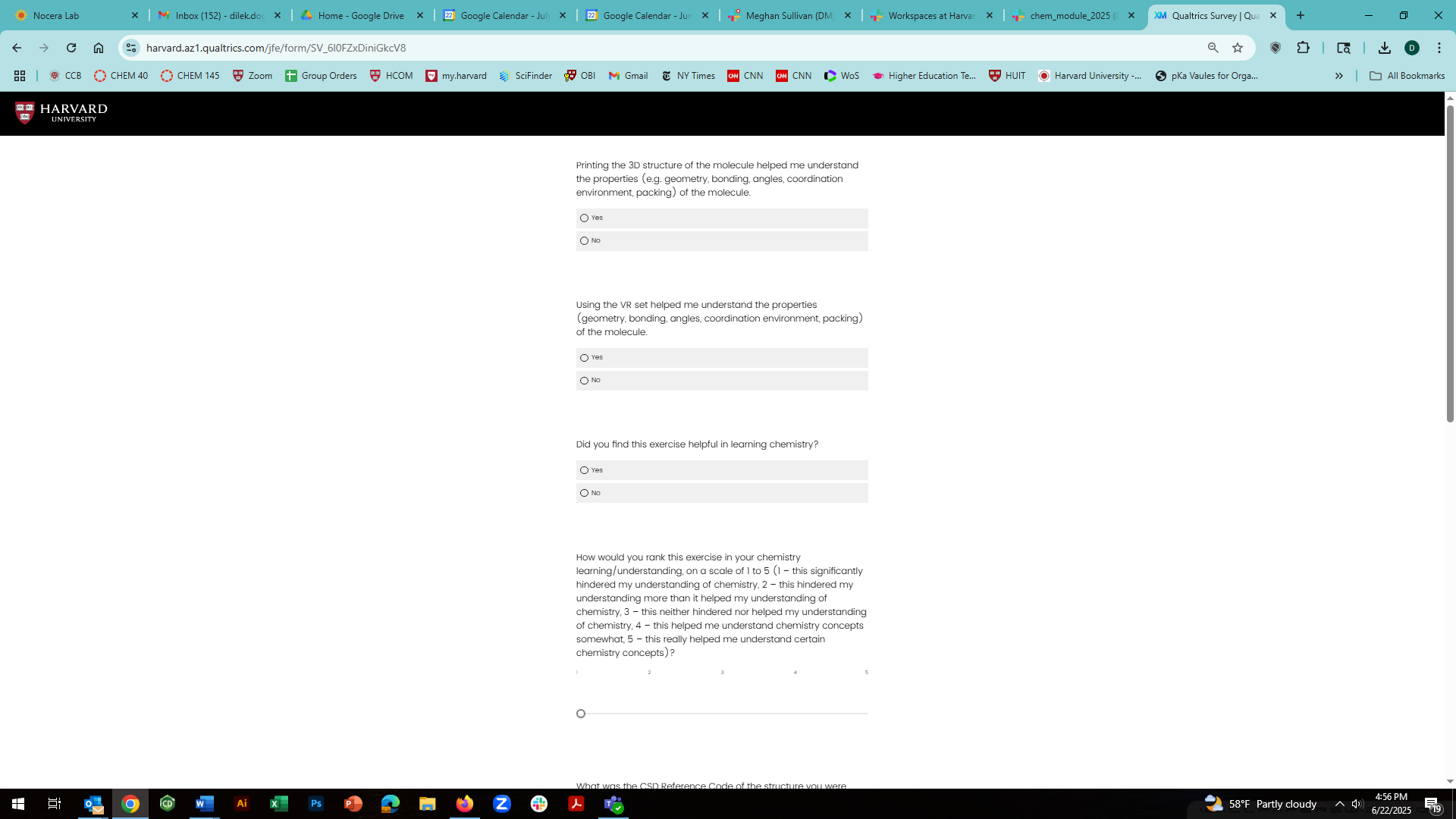


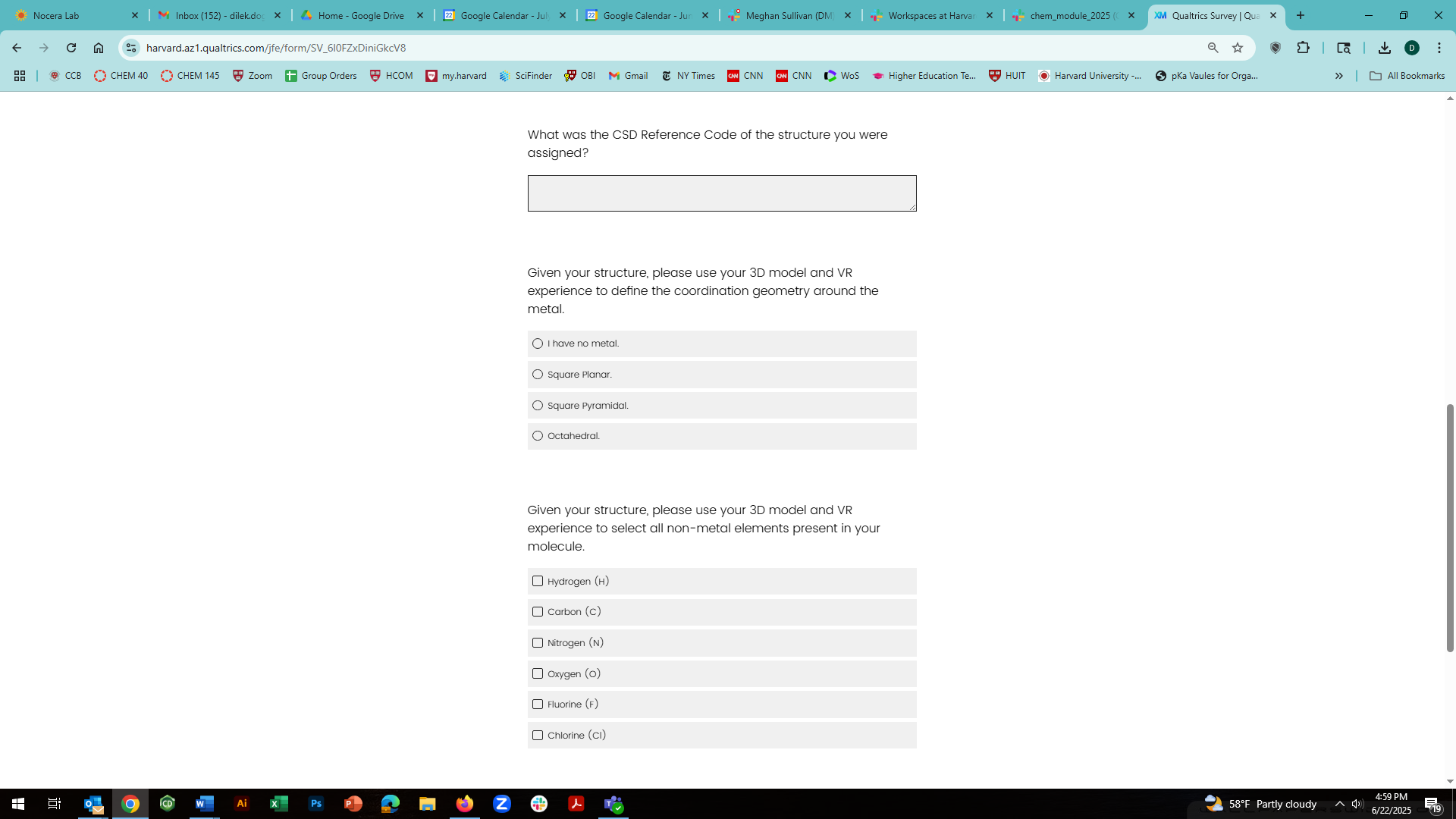


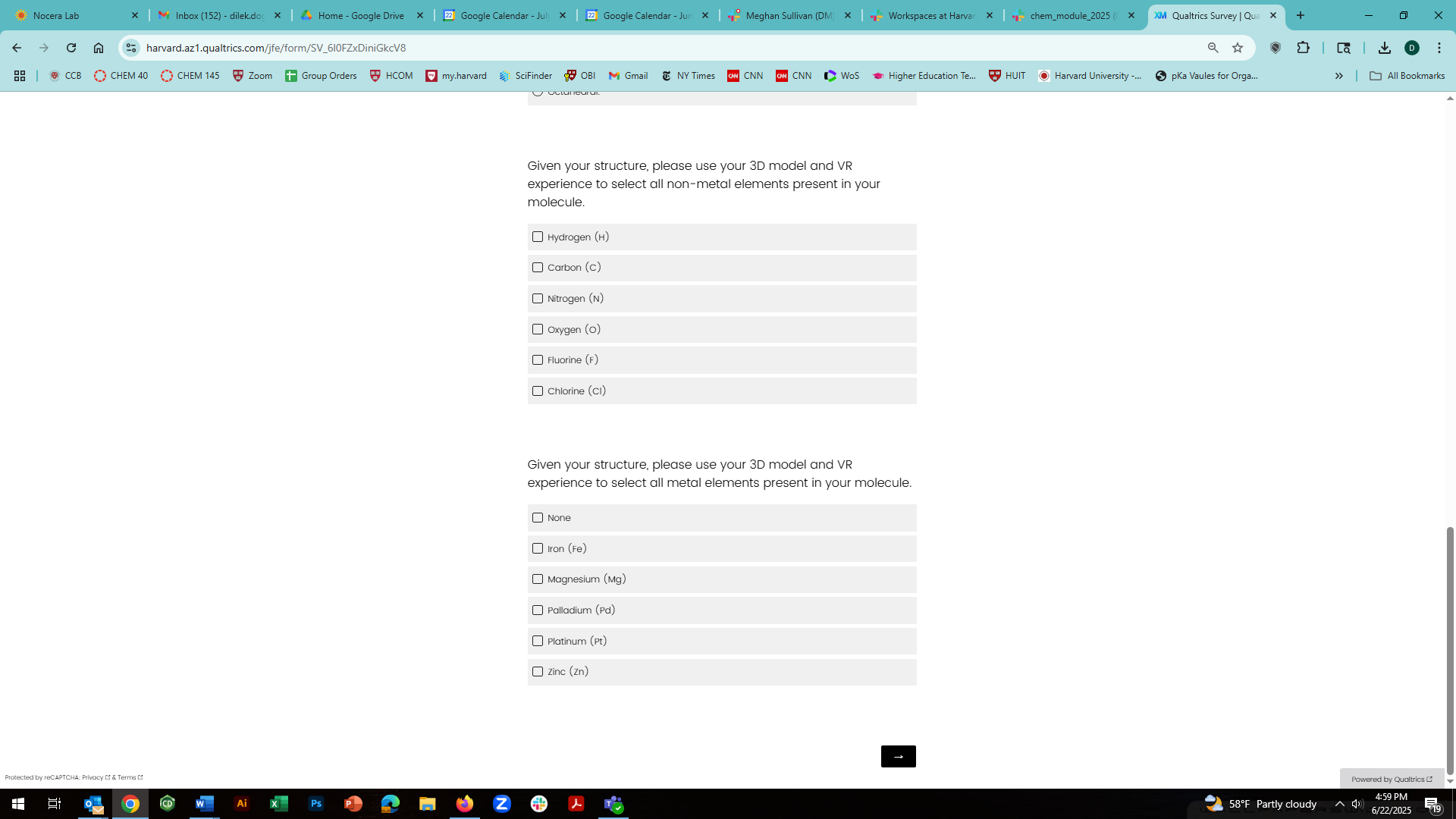


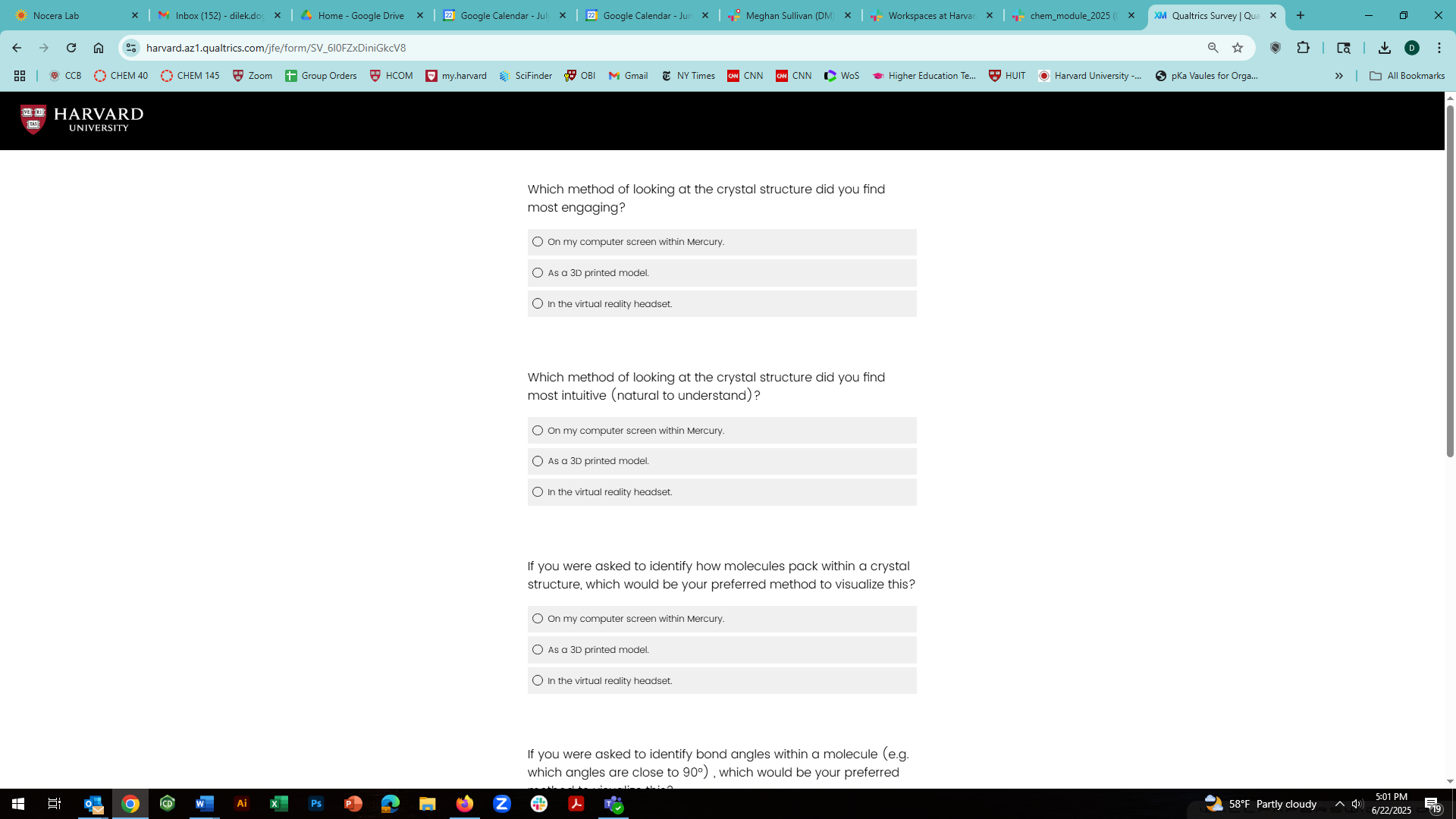


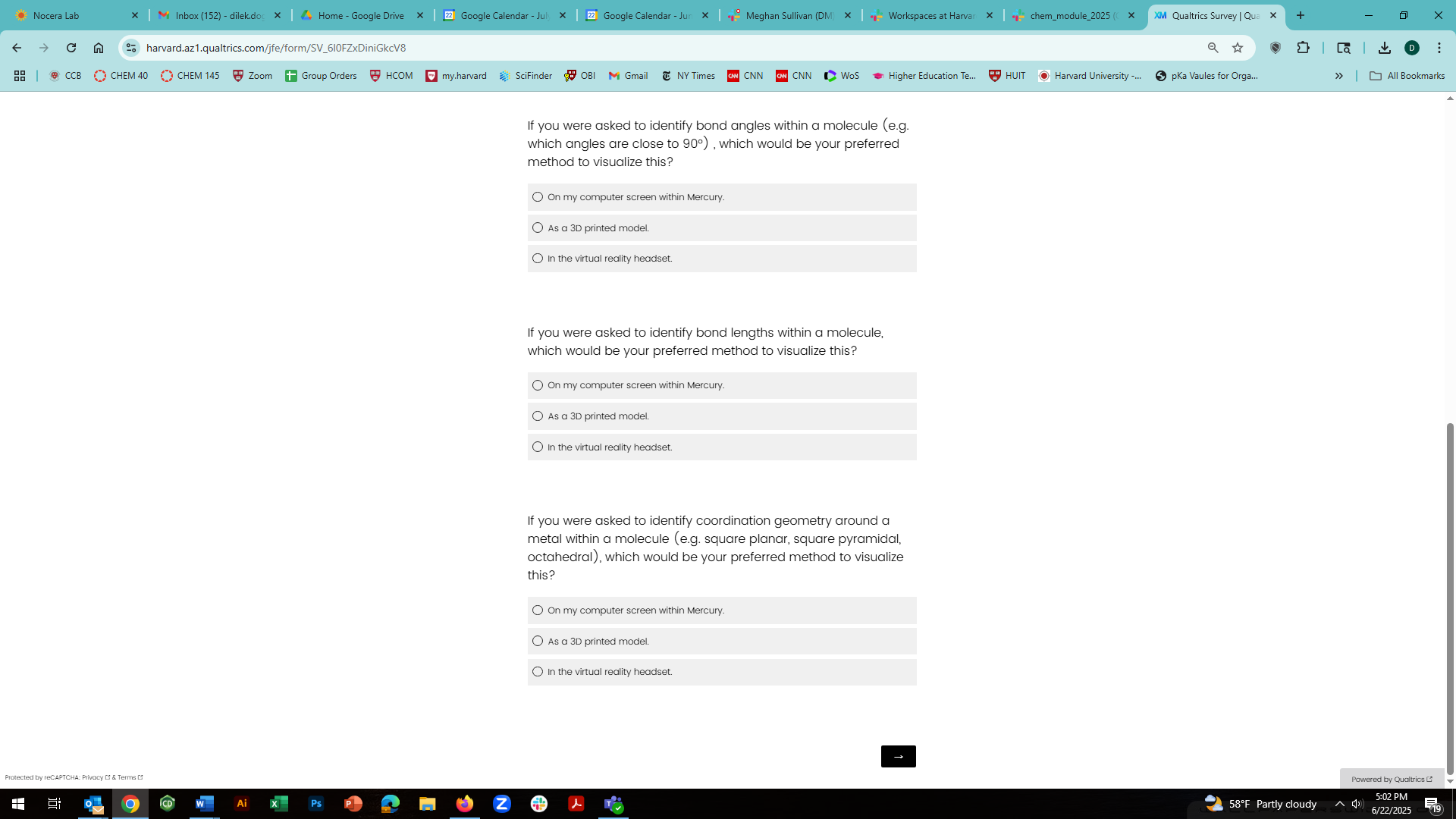


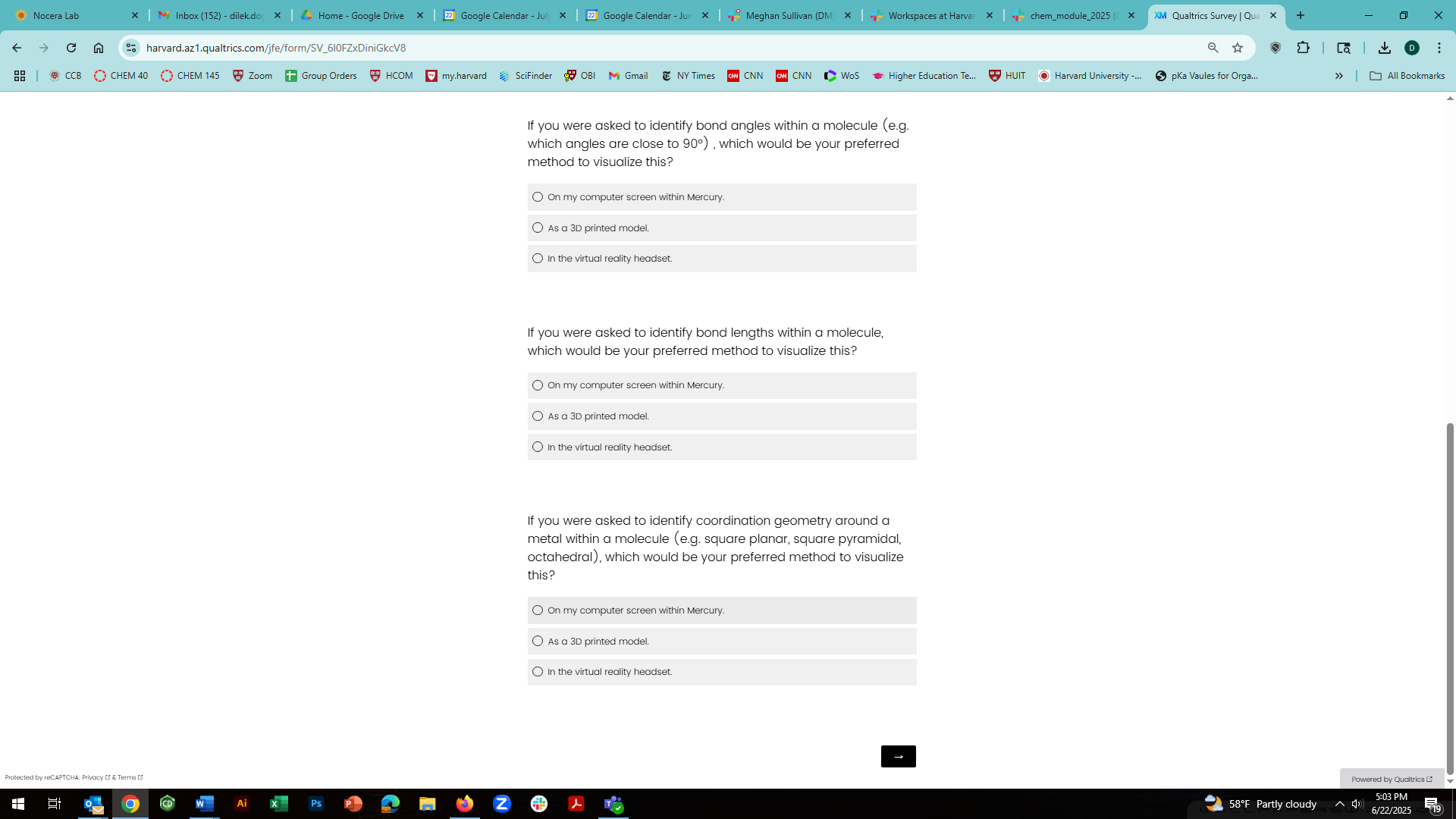


1. Appendix B Bambu Studio Settings and Results

To determine settings that would allow 3D printing of porphyrin molecules, a trial-and-error approach was taken, making modifications to settings between print attempts to address issues that were encountered. For example, in order to address lack of bed adhesion, raft layers were added, and the print speed was reduced; and in order to address difficulties in removing supports after the print completed, the support parameters were changed to add more space between the support and the model and to reduce the size and quantity of supports. The settings shown in Table S3 represent a set of values that was found to work for the molecules that were being printed in this specific module, but likely would need further modifications for other types of molecules and could be optimized to improve printing speed, if needed.

**Table S3** Bambu Studio Settings and Results for 3D print

| Setting | Initial Value | Modified Value | Setting | Initial Value | Modified Value |
| --- | --- | --- | --- | --- | --- |
| Speed Settings (mm/s) |  |  | Support Settings (mm) |  |  |
| Initial layer | 50 | 30 | Enable Support | False | True |
| Initial layer infill | 105 | 50 | Style | Default | Tree Slim |
| Outer wall | 200 | 100 | Threshold angle (deg) | 25 | 20 |
| Inner wall | 300 | 200 | On build plate only | False | True |
| Sparse infill | 330 | 150 | Raft layers | 0 | 2 |
| Internal solid infill | 300 | 200 | Top Z distance | 0.16 | 0.2 |
| Top surface | 200 | 100 | Support/object xy distance | 0.35 | 0.7 |
| Overhang speed |  |  | Max bridge length | 0 | 2 |
| 10% | 60 | 40 | Branch distance | 5 | 10 |
| 25% | 30 | 15 | Branch angle (deg) | 45 | 60 |
| 50% | 10 | 5 |  |  |  |
| 75% | 10 | 5 |  |  |  |
| 100% | 10 | 5 |  |  |  |
| Travel speed | 500 | 200 |  |  |  |

Summary of changes made to 3D printing settings for printing porphyrin molecules, starting from the Bambu Studio preset 0.16mm Optimal @BBL X1C.

Using the above settings, Table S4 below shows the print time and approximate cost in materials to print each of the four molecules that were used in this module. While in this case each molecule was printed in a separate job, the total print time could be reduced by combining several molecules into the same job, provided they were small enough to fit side by side on the build plate of the 3D printer.

**Table S4** Summary of 3D Print Times and Cost

| Molecule (CSD Refcode) | Print Time (h:mm) | Filament Weight (g) | Model Weight (w/o Support, g) | Approximate Cost (USD) |
| --- | --- | --- | --- | --- |
| Fe porphyrin (JUGYIX) | 1:33 | 12.34 | 5.14 | 0.25 |
| Mg porphyrin (JUGYET) | 2:11 | 16.80 | 8.00 | 0.34 |
| Pd porphyrin (CEPHEO) | 1:24 | 11.15 | 5.01 | 0.22 |
| Pt porphyrin (GOPZEU) | 1:12 | 9.04 | 5.04 | 0.18 |

Summary of print times and cost

**References**

Brown, C., Campbell, B. M., Chen, T., Darkwa, R. K., Kim, G., Kranchalk, D. J., Lamport, H., Le, C. M-D., Lu, J., McKnight, G. N., Nagelj, N., Seshadri, N. S., Reynolds, K. G., Zheng, S-L. & Dogutan. D. K. (2023). *J. Porphyrins Phthalocyanines.* **27**, 1650–1658.

Crisp, W., Fagan-Avery, S. A., Campbell, B. M., Morphet, D. R., Reynolds, K. G., Kudisch, B., Gonzalez, M. I., Zheng, S-H., Dogutan, D. K., & Nocera, D. G. (2022). *Inorg. Chem. Comm*. **146**, 109999 (1–5).

Chou, P., Kim. L., Marzouk, M. S., Sun, R., Hartnett, A. C., Dogutan, D. K., Zheng, S-H & Nocera, D. G. (2022). *ACS Omega*. **7**, 8988–8994.

Dash, Z. S., Huang, R. Q., Kimber, A. N., Olubajo, O. T., Polk, M., Rancu, O. P., Zhang, L. L., Fu, J., Nagelj, N., Reynolds, K. G., Zheng, S.-H. & Dogutan. D. K. (2024). Acta Crystallographica. Section C. **80**, 85–90.

TOC


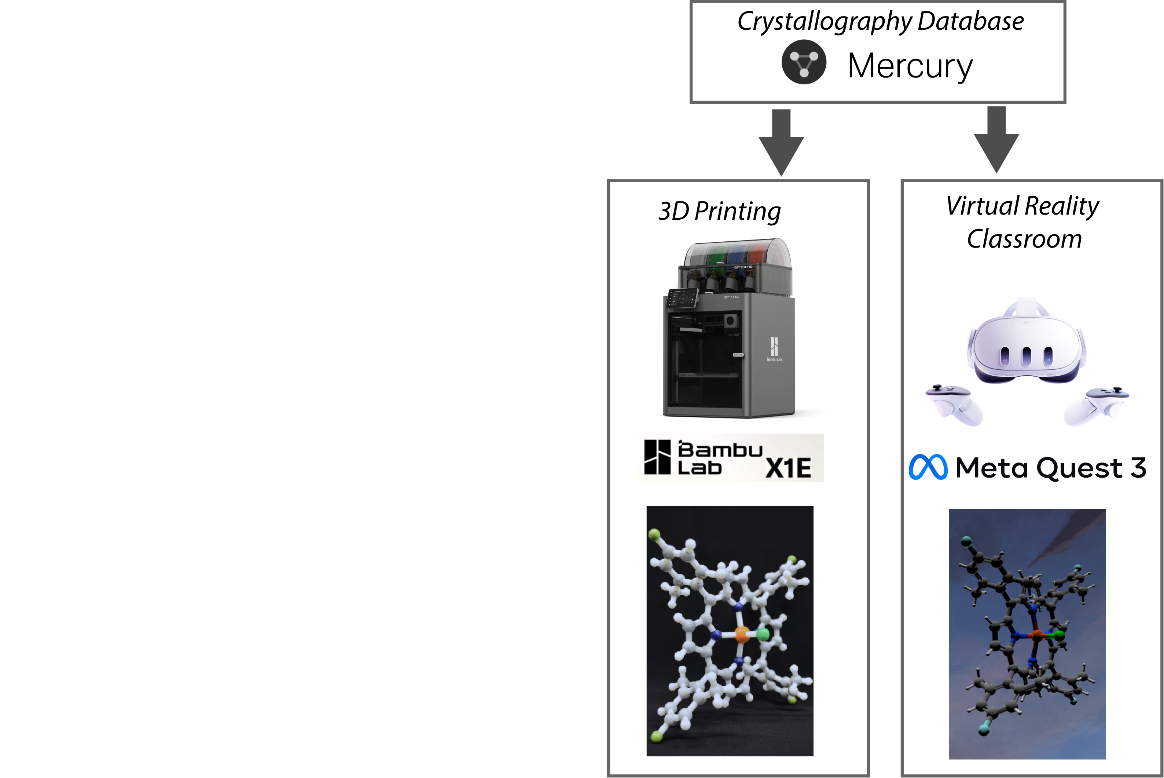

Supplement: Supplementary file 1 [file e-81-00889-sup2.docx]
